# Thymosin beta 4 as an Alzheimer disease intervention target identified using human brain organoids

**DOI:** 10.1016/j.stemcr.2025.102601

**Published:** 2025-08-14

**Authors:** Peng-Ming Zeng, Xin-Yao Sun, Yang Li, Wen-di Wu, Jing Huang, Dong-Dong Cao, Pin-jue Qian, Xiang-Chun Ju, Zhen-Ge Luo

**Affiliations:** 1 School of Life Science and Technology & State Key Laboratory of Advanced Medical Materials and Devices, ShanghaiTech University, Shanghai 201210, China; 2 Institute of Neuroscience, Center for Excellence in Brain Science and Intelligence Technology, Chinese Academy of Sciences, Shanghai 200031, China; 3 Okinawa Institute of Science and Technology Graduate University, Okinawa 904-0495, Japan

**Keywords:** cerebral organoids, thymosin beta 4, Alzheimer disease, amyloid precursor protein, disease intervention

## Abstract

The developmental origin of Alzheimer disease (AD) has been proposed but is arguably debated. Here, we developed cerebral organoids from induced pluripotent stem cells (iPSCs) with mutations in amyloid precursor protein (APP) associated with familial AD (fAD) and analyzed the dynamic changes of cellular states. We found that mature neurons induced in fAD organoids markedly decreased compared to that of health control, accompanied with increased cell senescence and β-amyloid (Aβ) production. Interestingly, the expression level of the gene *TMSB4X* that encodes thymosin beta 4 (Tβ4) significantly decreased both in fAD organoids’ neurons and AD patients’ excitatory neurons. Remarkably, the neurodevelopmental deficits and Aβ formation in fAD organoids were rescued by treatment with Tβ4. The beneficial effects of Tβ4 were also revealed in 5xfAD model mice. Thus, this study has identified Tβ4 as a neuroprotective factor that may mitigate altered neurogenesis and AD pathology, highlighting a potential for disease intervention.

## Highlights

• fAD organoids show increased Aβ, reduced neurons, and elevated apoptosis
• Τβ4 is downregulated in neurons of fAD organoids and excitatory neurons of patients
• Tβ4 treatment alleviates AD pathology in fAD organoids
• AAV-TMSB4X injection alleviates AD pathology in 5xFAD mice

## Graphical abstract

**Figure:**
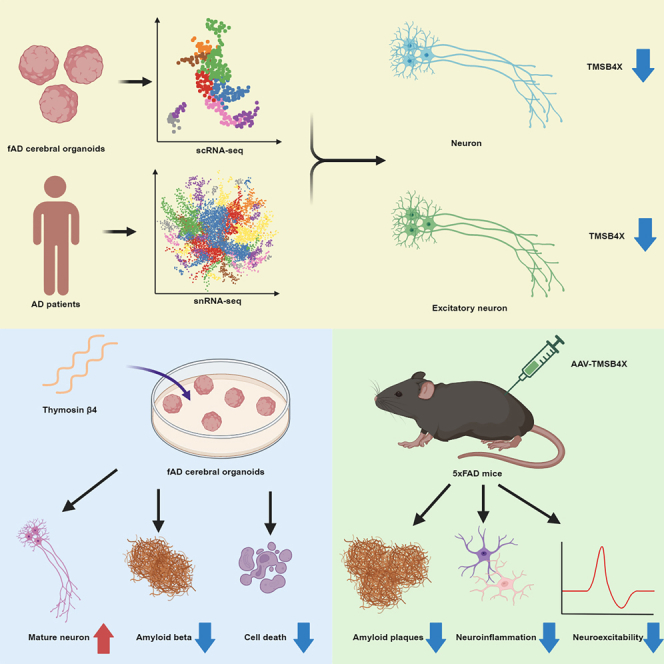

## Teaser

In this article, Luo and colleagues show that *TMSB4X*, which is downregulated in fAD organoid neurons and AD patients’ excitatory neurons, acts as a neuroprotective factor. Treatment with Tβ4, the product of *TMSB4X*, prevents cell death and attenuates AD pathology in fAD organoids. The therapeutic intervention role of Tβ4 is also confirmed in AD model mice.

## Introduction

Alzheimer disease (AD) is characterized by a range of cellular pathologies, including accumulation of β-amyloid peptide (Aβ), formation of neurofibrillary tangles (NFT), increased glial activation, and synaptic and neuronal loss (Hardy and Selkoe, 2002; Sato et al., 2018). In support of Aβ hypothesis, mutations in genes encoding amyloid precursor protein (APP) or subunits of γ-secretase, which cleaves APP to generate Aβ, have been found to be closely associated with AD occurrence and progression (Israel et al., 2012; Muratore et al., 2014).

It has been shown that long before the onset of AD pathology and behavior impairments, defects in brain structures and functions have been observed in AD patients (Busche and Konnerth, 2016; Crews et al., 2010). The earlier onset of neurogenesis defects preceding the core AD pathology including plaque and tangle formation has also been found in animal models harboring fAD mutations (Hamilton et al., 2010; Wen et al., 2004). Interestingly, treatment with Aβ has been shown to affect proliferation, differentiation, and survival of cultured human and rodent neural progenitor cells (Haughey et al., 2002). Thus, it is conceivable that future treatments to intervene the progression of AD may take effect when administered to risk people at early stages.

Identification of molecular targets for disease modification has relied on the analysis of human postmortem brain samples and animal models. However, the knowledge obtained using mouse models remain limited due to their developmental discrepancies compared with humans. The recently developed brain organoid model derived from induced pluripotent stem cells (iPSCs) has provided an opportunity to access the developmental process of human brain and model diseases *in vitro*, lifting the ethical issues related with limited accessibility of live human brains (Giandomenico and Lancaster, 2017). For instance, brain organoids from familial AD (fAD) iPSCs have shown AD-specific molecular features and pathology, as well as cell fate changes (Vanova et al., 2023; Zhao et al., 2020). However, these studies only focused on the description of the phenotypes in AD cerebral organoids but did not conduct further studies on specific pathogenic mechanisms, let alone the identification of intervention targets.

In this study, we generated fAD cerebral organoids from iPSCs with APP gene duplication and APPV717I mutation. The fAD cerebral organoids exhibited an increase in the level of Aβ, and alterations in cell types, as well as cell-type-specific transcriptomic changes. Furthermore, we compared the differential expression genes (DEGs) detected in fAD cerebral organoids with the single-nucleus RNA sequencing (snRNA-seq) data from AD patients and found a set of genes that showed similar tendency. We focused on functional studies on *TMSB4X*, which showed decrease both in neurons of fAD cerebral organoids and excitatory neurons of AD patients. The defects of neurogenesis and increase of Aβ in fAD cerebral organoids can be rescued by treatment with thymosin beta 4 (Tβ4), the protein product of *TMSB4X*. Overexpression of *TMSB4X* in neurons via AAV-TMSB4X rescued the pathological changes and mitigated neuronal hyper-excitability in 5xFAD mice. Thus, the fAD organoids developed in this study have shown early onset of neurogenesis defects and provided a platform for target screening and testing.

## Results

### Generation and verification of fAD cerebral organoids

**Figure 1:**
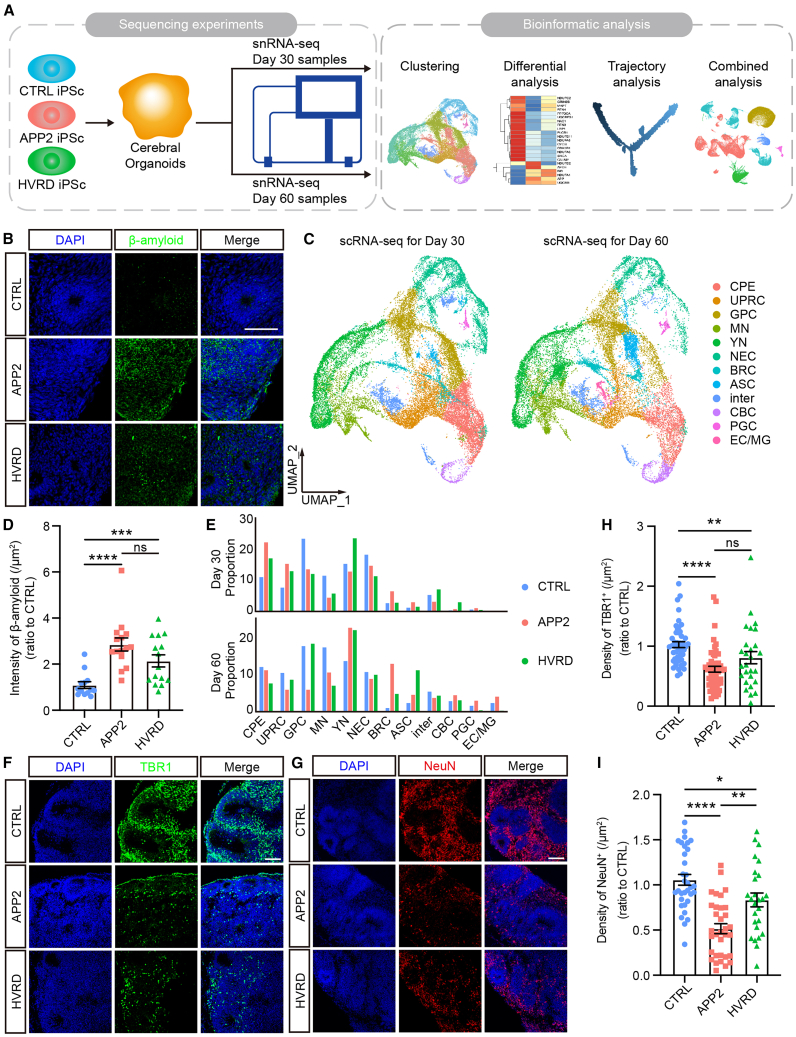
The cell composition difference between fAD and control cerebral organoids (A) Schematic representation of the iPSCs and organoids used in this study, sequencing experiments, and downstream bioinformatics analysis. (B) Immunofluorescence for β-amyloid in D90 cerebral organoids. Scale bars, 100 μm. (C) UMAP visualization of 12 major cell types isolated from D30 and D60 cerebral organoids. CPE, choroid plexus epithelial; UPRC, unfolded-protein-response-related cell; GPC, glia progenitor cell; MN, mature neuron; YN, young neuron; NEC, neuroepithelial cell; BRC, BMP-related cell; ASC, astrocyte; inter, intermediate; CBC, Cilia-bearing cell; PGC, proteoglycan-expressing cell; EC/MG, endothelia cell/microglia. (D) Quantification of the intensity of β-amyloid in D90 cerebral organoids. Data are presented as mean ± SEM of at least 13 organoids per group from four independent experiments. Mann-Whitney test. ^∗∗∗^*p* < 0.001, ^∗∗∗∗^*p* < 0.0001. (E) The proportions of major cell types in fAD and control cerebral organoids at D30 (up) and D60 (down). (F) Immunofluorescence for sixth layer neuron marker TBR1 in D30 cerebral organoids. Scale bars, 100 μm. (G) Immunofluorescence for mature neuron marker NeuN in D60 cerebral organoids. Scale bars, 100 μm. (H and I) Quantification of the density of TBR1-positive cells in D30 organoids (H) or the density of NeuN-positive cells in D60 organoids (I). Data are presented as mean ± SEM of at least nine organoids (three fields per organoid) per group from at least three independent experiments. The value of the control group was normalized as 1.0. Mann-Whitney test. ^∗^*p* < 0.05, ^∗∗^*p* < 0.01, ^∗∗∗∗^*p* < 0.0001. See also Figures S1 and S2 and Table S1.

Three iPSCs cell lines including two fAD iPSCs and one control iPSC were used in single-cell RNA sequencing (scRNA-seq) (Table S1). The two fAD iPSCs carry *APP* gene duplication mutation (APP2) and *APPV717I* single-site mutation (HVRD), respectively, which are positively associated with AD pathology caused by increased Aβ due to higher APP level or increased processing (Israel et al., 2012; Muratore et al., 2014). The cerebral organoids were generated using the protocol as described in previous studies (Lancaster and Knoblich, 2014) and then analyzed for cell composition using scRNA-seq and immunostaining at different days (D30, D60, or D90) (Figure 1A). As expected, abundant neural progenitors (NPs) were present in D30 organoids as reflected from positive signals of stem cell marker SOX2, radial glia marker PAX6, and intermediate progenitor marker TBR2 (Figure S1A), and neurons gradually became mature later on as indicated by signals for pan-neuronal marker MAP2, mature neuron marker NeuN, and cortical layer markers TBR1 and CTIP2 (Figure S1B). We also determined levels of Aβ and found that the fAD organoid exhibited marked increase in the intensity of Aβ at D90 (Figures 1B and 1D). For the soluble Aβ, the ratio of Aβ1-42/Aβ1-40 significantly decreased in the medium of fAD organoids at D60 (Figures S1C–S1E). This finding aligns with prior studies documenting altered Aβ1-42/Αβ1-40 ratios in the cerebrospinal fluid of AD patients (Delaby et al., 2022). Thus, the fAD cerebral organoids exhibited AD-related changes after maturation.

The cell composition and heterogeneity were also revealed by scRNA-seq, which showed the presence of 12 cell types, after dimensionality reduction and cell-type clustering (Figure 1C). The annotation for these clusters was based on the expression of their marker genes and Gene Ontology (GO) terms enriched (Figure S2A; see methods). The limited *GAD1* expression and pronounced *SLC17A7* expression (Figure S2B) indicated the preference for generating excitatory neurons. It appeared that D60 organoids contained more mature neurons than D30 organoids (Figures 1C and 1E), and the proportion of several cell types between the control and fAD organoids displayed different distribution (Figure 1E). Interestingly, both APP2 and HVRD organoids showed reduction of mature neurons (MN) at 30 and 60 days and increase of young neurons (YN), BMP-related cells (BRC), and astrocytes (ASC) at 60 days, suggesting an impairment in neuronal maturation (Figure 1E). The expression of sixth layer neuron (*TBR1*) and mature neuron markers (NeuN/*RBFOX3*) showed marked reduction in both of the fAD cerebral organoids compared to the control (Figures S2C and S2D). In line with this notion, immunostaining results showed that TBR1 signal in D30 organoids or NeuN signals in D60 organoids decreased in both APP2 and HVRD organoids (Figures 1F–1I).

### Cell-type-specific transcriptomic changes in fAD cerebral organoids

**Figure 2:**
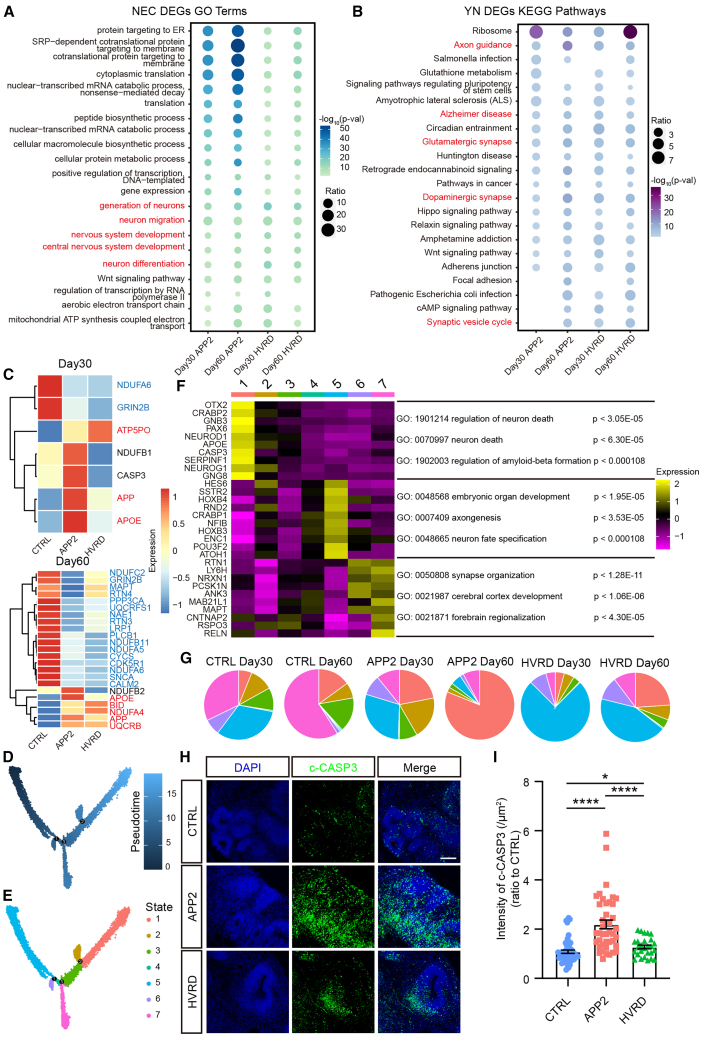
Enrichment of neuronal clusters with AD-like features in fAD cerebral organoids (A) The top enriched GO terms in neuroepithelial cells of each fAD cerebral organoid. (B) The top enriched KEGG pathways in young neurons of each fAD cerebral organoid. (C) Heatmap for the expression of differential expressed genes, enriched in Alzheimer’s disease pathway, in young neurons at 30 and 60 days, respectively. The red color means that the gene exhibits a consistent upregulation in its differential expression within APP2 and HVRD organoids. The blue color means that the gene exhibits a consistent downregulation within APP2 and HVRD organoids. (D and E) Differentiation pathways labeled by pseudotime (D) or cell states (E) in neurons (young neurons and mature neurons). (F) Heatmap showing the top 10 marker genes in state 1, 5, and 7 cells in neurons with specific GO terms listed. (G) The proportions of neuronal states in fAD and control cerebral organoids. (H) Immunofluorescence for apoptosis signal (c-CASP3) in D60 cerebral organoids. Scale bars, 100 μm. (I) Quantification of the intensity of c-CASP3 in D60 cerebral organoids. Data are presented as mean ± SEM of at least nine organoids (three fields per organoid) per group from at least three independent experiments, with the value of control group normalized as 1.0. Mann-Whitney test. ^∗^*p* < 0.05, ^∗∗∗∗^*p* < 0.0001. See also Figure S3.

Then we compared transcriptome profiles of all cell types between fAD and control organoids at D30 and D60, respectively (Figure S2E). Notably, more DEGs were observed only in a certain cell type, and only limited numbers of DEGs were present across multiple cell types, indicating the cell-type-specific differences in fAD organoids (Figure S2F). Next, we analyzed biological functions of these DEGs in various cell types using the GO and Kyoto Encyclopedia of Genes and Genomes (KEGG) analysis. We found that DEGs in neuroepithelial cells (NECs), which represent neural stem cell populations contributing to early neurogenesis, were enriched with terms including “generation of neurons” and “neuron differentiation” (Figure 2A). Meanwhile, the DEGs in YN were enriched with “axon guidance” and “glutamatergic synapse” pathways, which are important for synapse formation in neurons, especially excitatory neurons (Figure 2B). These results agree with the fact that fAD cerebral organoids displayed neuronal maturation defects as shown in a previous study (Ghatak et al., 2021). Interestingly, the DEGs in YN were also enriched with “Alzheimer disease” pathway, suggesting that molecular features of AD already emerged in neurons at early differentiation stage (Figure 2B). We then analyzed the expression of these DEGs enriched in the “Alzheimer disease” pathway and defined them as AD-related DEGs (Figure 2C). With the increase of the culture time, the D60 fAD organoids showed more AD-related DEGs than that at D30 (Figure 2C). Both the *APP* and *APOE* genes, which are associated with a high risk of AD as well as aging and apoptosis in neurons (Wang et al., 2014; Zhao et al., 2022), were found to be highly expressed in the neurons of D30 and D60 fAD organoids. Notably, genes encoding mitochondrial membrane proteins related to aerobic electron transport chain, including *NDUFA4*, *NDUFA6*, and *NDUFB11*, were observed in D60 fAD organoids (Figure 2C). These results indicate the activation of neuronal apoptosis and mitochondrial dysfunction in early differentiated neurons in fAD organoids.

The presence of apoptotic state of differentiated neurons in fAD organoids was also confirmed by pseudotime trajectory analysis, which showed two branched neuronal differentiation pathways comprised of seven cell states, with states 1 and 7 representing two distinct terminal states (Figures 2D and 2E). GO analysis showed that genes associated with neuronal fate specification and organ development were highly expressed in the original state (state 5), genes related with Aβ formation and cell death were highly expressed in state 1, and genes associated with synapse organization and brain regionalization were highly expressed in state 7 (Figure 2F). Interestingly, fAD organoids contained more neurons at state 1 and less neurons at state 5 at D60 (Figure 2G). In line with this notion, both the signal of cleaved caspase3 (c-CASP3) and TUNEL were increased in fAD organoids (Figures 2H, 2I, S3A, and S3B). The destination to cell death state may be due to the retardation of neuronal maturation. These results imply that the accumulation of AD-like molecular and cellular features may be originated from early brain developmental stages.

**Figure 3:**
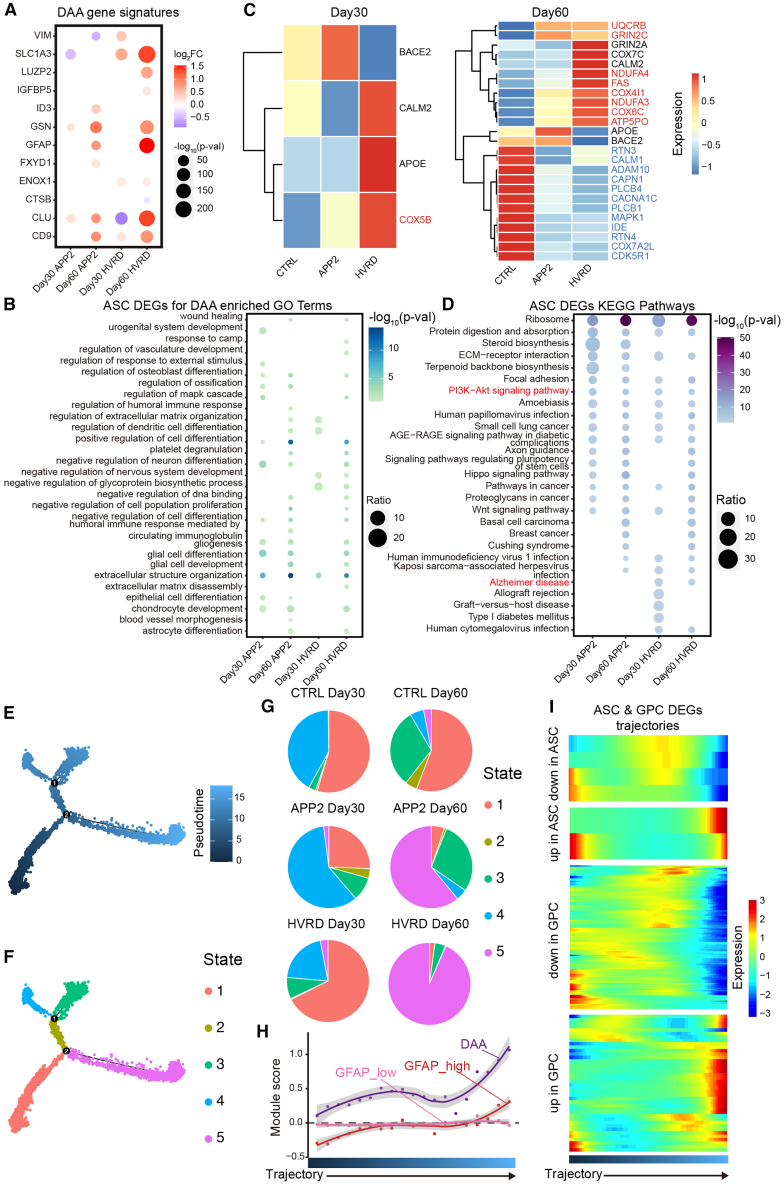
AD-like features in glial linage cells in fAD cerebral organoids (A) The differential expression of the DAA gene signatures in astrocytes of fAD cerebral organoids. (B) The DAA-enriched GO terms in astrocytes of each fAD cerebral organoid. (C) Heatmap for the expression of differential expressed genes, enriched in Alzheimer’s disease pathway, in astrocytes of D30 and D60 organoids. The red color means that the gene exhibits a consistent upregulation in its differential expression within APP2 and HVRD organoids. The blue color means that the gene exhibits a consistent downregulation within APP2 and HVRD organoids. (D) The top enriched KEGG pathways in astrocytes of each fAD cerebral organoid. (E and F) The differentiation pathways labeled by pseudotime (E) or cell states (F) in glia progenitor cells and astrocytes. (G) The proportions of cell states identified from glia progenitor cells and astrocytes in fAD and control cerebral organoids. (H) Module scores for DAA (*GFAP*, *CSTB*, *VIM*, *OSMR*, *GSN*), GFAP^high^ (*GFAP*, *ID3*, *AQP4*, *MYOC*, *ID1*, *FABP7*) and GFAP^low^ (*LUZP2*, *SLC7A10*, *MFGE8*) gene signatures averaged for nuclei in each of the 25 trajectory bins. Solid color lines represent LOESS regressions for each signature, and the gray outlines represent 95% CIs. (I) Heatmap for differential expressed genes identified in astrocytes and glia progenitor cells.

In addition to neuronal cells, we also analyzed DEGs and trajectory of glia linage cells. The snRNA-seq study for the hippocampus from 5xFAD mouse have classified astrocytes into GFAP^low^, GFAP^high^, and disease-associated astrocyte (DAA) subtypes (Habib et al., 2020). The gene signatures in DAA, including *GSN*, *GFAP*, *CLU*, and *CD9*, were upregulated in fAD cerebral organoids, and this tendency was more remarkable at D60 compared to D30 (Figure 3A). In line with this notion, DAA-related GO terms were also enriched in ASC DEGs (Figure 3B). Unlike the results in neurons, only the ASC DEGs from HVRD organoids showed enrichment in the “Alzheimer’s disease” pathway (Figure 3D). In addition, the “PI3K-Akt signaling pathway,” which is related to astrocyte activation (Pang et al., 2023), was consistently enriched across all four fAD organoids (Figure 3D). We also determined the expression of the ASC DEGs associated with “Alzheimer disease” pathway (Figure 3C). Like in neurons, mitochondrial-related genes *COX7A2L* and *UQCRB* showed differential expression in ASC of fAD organoids (Figure 3C). Among the genes associated with AD, *UQCRB*, *RTN3*, *PLCB1*, and *RTN4* were consistently differentially expressed in both YN and ASC of fAD organoids (Figures 2C and 3C). The decreased expression of *RTN3* and *RTN4* has been shown to be associated with increased production of *BACE1*, resulting in accumulation of APP cleavage products (Murayama et al., 2006). These results indicate that astrocytes in our fAD organoids display AD-like features.

The visualization of glia progenitor cells (GPCs) and ASC subclasses and pseudotime trajectory analysis showed starting (state 1) and distinct final states (states 3 and 5) of astrocyte (Figures 3E and 3F). In D30 organoids, the distribution of cells at different states displayed big discrepancy. However, in D60 organoids, the APP2 and HVRD groups exhibited common increase in the proportion of cells at state 5 and a decrease of cells at state 2 (Figure 3G). Based on the expression level of *GFAP* and combined DAA genes, we analyzed the GFAP^low^, GFAP^high^, and DAA module score along this trajectory (Figure 3H; see methods). At the end of trajectory, the fAD cerebral organoids preferred to differentiate to the fates with high score of the GFAP^high^ and DAA modules (Figure 3H). Thus, the developmental trajectory of glia linage cells in the fAD cerebral organoids resembled disease-related alternations in the astrocytes of AD patients (Morabito et al., 2021). Furthermore, the upregulated DEGs of ASC and GPC in fAD organoids were highly expressed at the end of the trajectory, and the downregulated DEGs were highly expressed at the start and the middle of the trajectory (Figure 3I), indicating again the recurrence of disease-associated states of astrocytes in fAD organoids.

### Comparison for gene expression profiles in fAD cerebral organoids and postmortem brain samples

**Figure 4:**
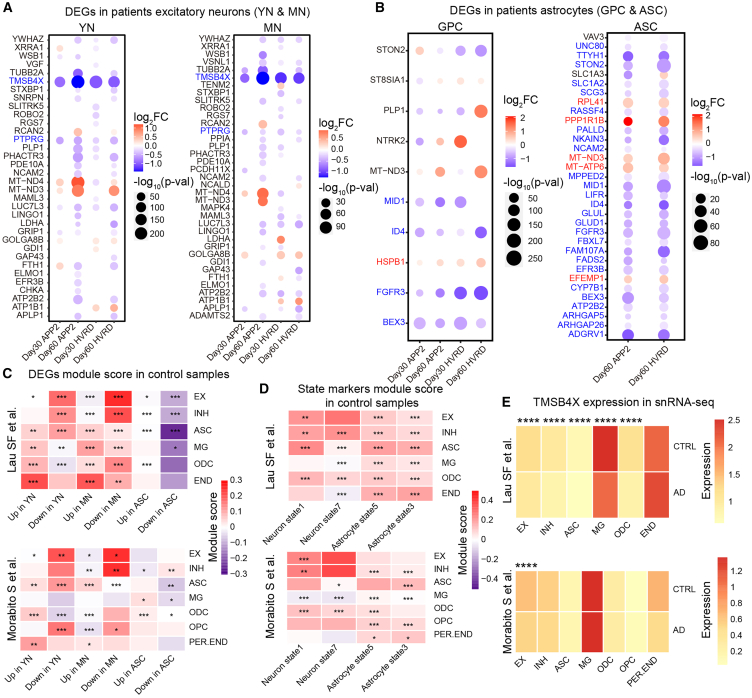
Conjoint analysis of fAD cerebral organoids and patients’ data (A) Differential expressed genes identified in excitatory neurons of AD patients, in young neuron (left) and mature neuron (right) of fAD cerebral organoids. (B) Differential expressed genes identified in astrocytes of AD patients, in glia progenitor cell (left) and astrocyte (right) of fAD cerebral organoids. (C and D) Heatmap showing the module scores of differential expressed genes identified from young neurons, mature neurons, and astrocyte (C) or markers identified in pseudotime trajectory analysis (D) of fAD cerebral organoids in major cell types of control brain samples. EX, excitatory neuron; INH, inhibitory neuron; ASC, astrocyte; MG, microglia; ODC, oligodendrocyte; OPC, oligodendrocyte progenitor; END, endothelial; PER.END, pericytes endothelial. Differences of module scores between control and AD patients were tested by Wilcoxon rank test, and *p* values were overlaid on the heatmap (^∗^*p* < 0.05, ^∗∗^*p* < 0.005, ^∗∗∗^*p* < 0.0005). (E) Heatmap showing the expression of *TMSB4X* in cell types of control and AD patients. ^∗∗∗∗^p.adj <0.0001. See also Figure S4.

Then we analyzed two single-cell datasets from AD patients (Database: GSE157827 and GSE174367) (Lau et al., 2020; Morabito et al., 2021) (Figures S4A and S4B) and compared with our scRNA-seq data of cerebral organoids. The cell-type-specific DEGs for AD patients were analyzed with the same threshold values used in cerebral organoids (Figures S4C and S4D). Among the DEGs in excitatory neurons, 85 genes showed consistent changes in two sets of patient data, and interestingly, only *TMSB4X* and *PTPRG* genes showed decreased expression in both YN and MN of the four types of fAD cerebral organoids (Figure 4A). *PTPRG* has been identified as a risk gene associated with AD (Herold et al., 2016), and *TMSB4X* has been reported to be a potential anti-inflammatory factor against neurodegenerative disorder (Shomali et al., 2020). In addition, we analyzed DEGs in GPC of all four fAD organoids and ASC of D60 fAD organoids, respectively (Figure 4B). Among them, five genes (*HSPB1*, *MID1*, *ID4*, *FGFR3*, and *BEX3*) showed consistent changes in GPC across four fAD cerebral organoids compared to the corresponding control organoids (Figure 4B). The increased expression of small heat shock protein (*HSPB1*) in astrocytes has been thought to be related to the external stimulation stress of astrocytes, and some studies have shown a certain interaction between *HSPB1* and Aβ (Nafar et al., 2016; Wilhelmus et al., 2006). Meanwhile, other four decreased genes (*MID1*, *ID4*, *FGFR3*, and *BEX3*) also showed the same tendency in ASC of D60 fAD organoids. Notably, several mitochondrial genes, including *MT-ND3* and *MT-ATP6*, were upregulated in YN, MN, and ASC of D60 fAD organoids, consistent with the results of a recent scRNA-seq analysis of a large cohort of AD patients, whose mitochondrial gene expression was significantly upregulated in excitatory neurons, astrocytes, and microglia (Mathys et al., 2023).

We calculated the module scores for the DEGs in YN, MN, or ASC of fAD organoids in specific cell types of control samples and detected the difference of these module scores between control samples and AD patients (Figure 4C; see methods). We found that the module scores of DEGs in YN and MN showed significant difference in excitatory neurons (EX) between control samples and AD patients, whereas only that of “Down in ASC” showed consistent differences in the astrocytes of two patient datasets (Figure 4C). Furthermore, the module scores for “Down in YN” and “Down in MN” showed high score in excitatory neurons but the module scores for “UP in YN” showed high score in endothelial cells (END) (Figure 4C). This result implies that the genes downregulated in the neurons of fAD organoids are highly expressed in the neurons of healthy brains, whereas the genes upregulated in fAD organoids are expressed in non-ectodermal cells of the brain. We also analyzed the module scores of state marker genes of neurons and astrocytes detected in trajectory analysis of cell fates in fAD organoids (Figure 4D). We found that the module scores for marker genes of AD-prone state 1 neurons showed significant difference in EX, inhibitory neurons (INH), and oligodendrocytes (ODC) in AD patients versus controls (Figure 4D). And marker genes of normal-prone state 3 in organoid ASC also showed marked differences in the astrocytes from AD patients (Figure 4D). Thus, alterations in cell state trajectory observed in fAD organoids recapitulate to some extent the real cellular changes in AD patients.

The two snRNA-seq datasets of AD patients showed that *TMSB4X* was consistently downregulated in excitatory neurons (Figure 4E). Next, we determined the *TMSB4X* expression levels in organoids. Both fAD organoids (APP2 and HVRD2) exhibited marked downregulation of *TMSB4X* compared to controls (CTRL and CTRL2) (Figure S4E). Thymosin β4 (Tβ4), the product of *TMSB4X*, has been used to treat diverse diseases, such as pressure ulcers (Francis Godschalk, 2007) and dry eye syndrome (Sosne et al., 2012). A recent study has shown that Tβ4 reverses cognitive impairment in mouse model of AD via regulation of microglia polarization and inflammatory response (Wang et al., 2021). Analysis of STRING database (string-db.org) has revealed a set of *TMSB4X* interacting genes (Figure S4F). The top KEGG pathways and GO terms were associated with apoptosis, AD, cytoskeleton, and neurotrophin signaling (Figures S4G and S4H).

To validate the association between *TMSB4X* and AD, control organoids were treated with 5 μM Aβ at day 20 of differentiation, with sampling at day 60 to assess *TMSB4X* expression (Figure S4I). Quantitative analysis revealed a significant downregulation of *TMSB4X* in Aβ-treated organoids (Figure S4J), recapitulating the expression pattern observed in disease contexts. These findings highlight a functional link between *TMSB4X* and AD pathology.

### Tβ4 rescues the neurodevelopmental defects and reduces Aβ in fAD cerebral organoids

**Figure 5:**
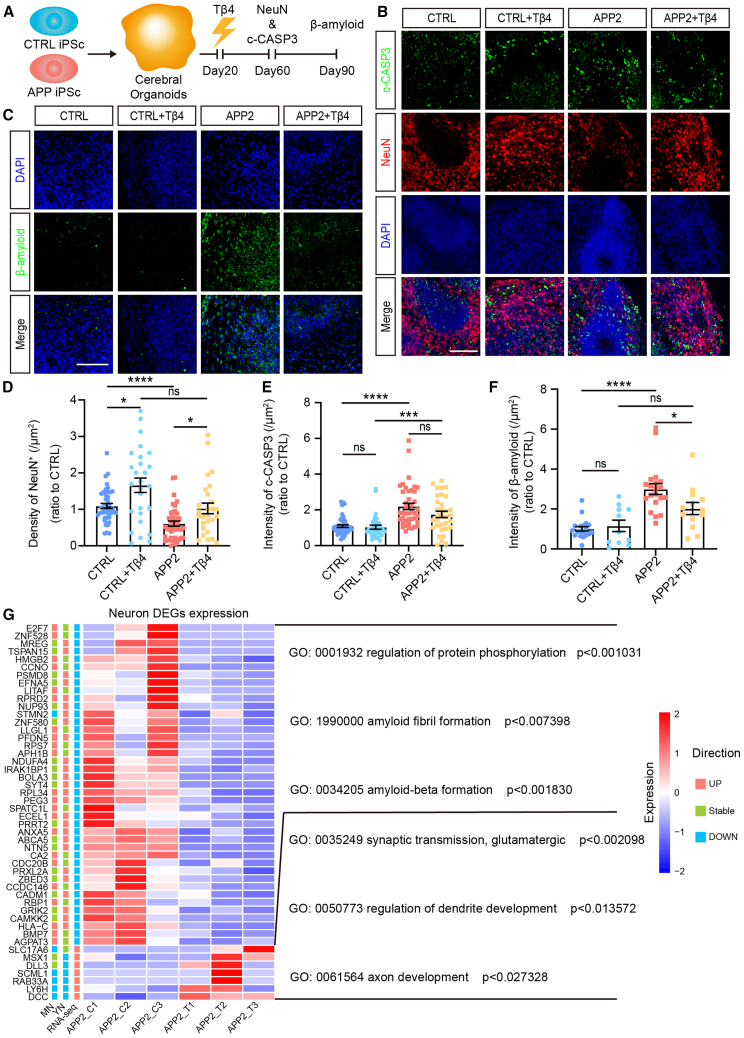
Thymosin β4 protects neurons and attenuates Aβ production in fAD cerebral organoids (A) Schematic representation of thymosin β4 treatment of fAD cerebral organoids and timeline of the analysis. (B) Immunofluorescence for mature neuron (NeuN) and apoptosis signal (c-CASP3) in D60 cerebral organoids. Scale bars, 100 μm. (C) Immunofluorescence for Aβ in D90 cerebral organoids. Scale bars, 100 μm. (D and E) Quantification of the density of NeuN-positive cells (D) and the intensity of c-CASP3 signal (E) in D60 cerebral organoids. Data are presented as mean ± SEM of at least nine organoids (three fields per organoid) per group from at least three independent experiments, with the value of control group normalized as 1.0. Mann-Whitney test. ^∗^*p* < 0.05, ^∗∗∗^*p* < 0.001, ^∗∗∗∗^*p* < 0.0001. (F) Quantification of the intensity of β-amyloid in D90 cerebral organoids. Data are presented as mean ± SEM of at least 11 organoids per group from at least three independent experiments, with the value of control group normalized as 1.0. Mann-Whitney test. ^∗^*p* < 0.05, ^∗∗∗∗^*p* < 0.0001. (G) Heatmap showing the differential expressed genes identified in young neurons and mature neurons of Tβ4-treated (T1, T2, and T3) D30 APP2 cerebral organoids with specific GO terms listed. Vehicle treatments were used as controls (C1, C2, and C3). See also Figures S5 and S6.

To determine the effects of Tβ4, control and APP2 cerebral organoids were treated with Tβ4 (0.5 μg/mL) at D20 followed by examination at D60 and D90 (Figure 5A). We found that Tβ4 treatment markedly increased the density of neurons in both control and APP2 D60 organoids (Figures 5B and 5D). APP2 organoids exhibited increased intensity of c-CASP3 signals and TUNEL signals, suggesting the elevation of cell death (Figures 5B, 5E, S5A, and S5B). Notably, Tβ4 treatment had no effect on c-CASP3 signal in APP2 or control organoids (Figure 5E), but significantly inhibited TUNEL intensity in APP2 D60 cerebral organoids (Figure S5B). Furthermore, Tβ4 significantly decreased the level of Aβ in APP2 D90 cerebral organoids (Figures 5C and 5F) and increased the ratio of Aβ1-42/Aβ1-40 in APP2 D60 cerebral organoids (Figures S5C–S5E). These results suggest that Tβ4 treatment attenuates AD-specific features in fAD cerebral organoids.

To investigate the mechanisms of Tβ4 function in fAD cerebral organoids, we performed bulk RNA-seq analysis for D30 APP2 cerebral organoids treated with and without Tβ4, respectively. We found that Tβ4-treated fAD organoids exhibited 911 upregulated genes and 1,395 downregulated genes (Figure S6A). The upregulated genes were enriched in the GO terms related to neurodevelopmental pathways such as “central nervous system neuron axonogenesis” and “detection of calcium ion” (Figure S6B). And the downregulated genes were enriched in some immune response and protein phosphorylation terms like “somatic recombination of immunoglobulin genes involved in immune response” and “negative regulation of phosphorylation” (Figure S6C).

To corroborate the role of Tβ4 in regulating AD-associated signaling, we performed a joint analysis of bulk RNA-seq data of Tβ4-treated APP2 organoids and scRNA-seq data of APP2 organoids (Figures 5G and S6D). Among the DEGs of fAD organoid neurons (YN and MN), 41 genes were downregulated and 7 genes were upregulated in Tβ4-treated organoids (Figure 5G). Several downregulated genes, such as *APH1B* and *NDUFA4*, were involved in “Alzheimer’s disease” pathway. *APH1B* gene encodes Aph-1b, one of the four subunits (Aph-1, nicastrin, presenilin, and Pen-2) of γ-secretase (Gertsik et al., 2014). The decrease of *APH1B* might inhibit γ-secretase-mediated processing of APP. The downregulated genes were related to amyloid-beta formation, while Tβ4 upregulated genes focused on neuron and synapse development (Figure 5G). We also analyzed the effects of Tβ4 on gene expression of GPC and ASC DEGs (Figure S6D). The treatment with Tβ4 was found to reverse the upregulation of *TMEM132C* and *PEG3*, and the downregulation of *DHCR7* observed in the ASC and GPC of D30 APP2 organoids.

### Administration of AAV-TMSB4X alleviates pathological changes and neuronal excitability in 5xFAD mice

**Figure 6:**
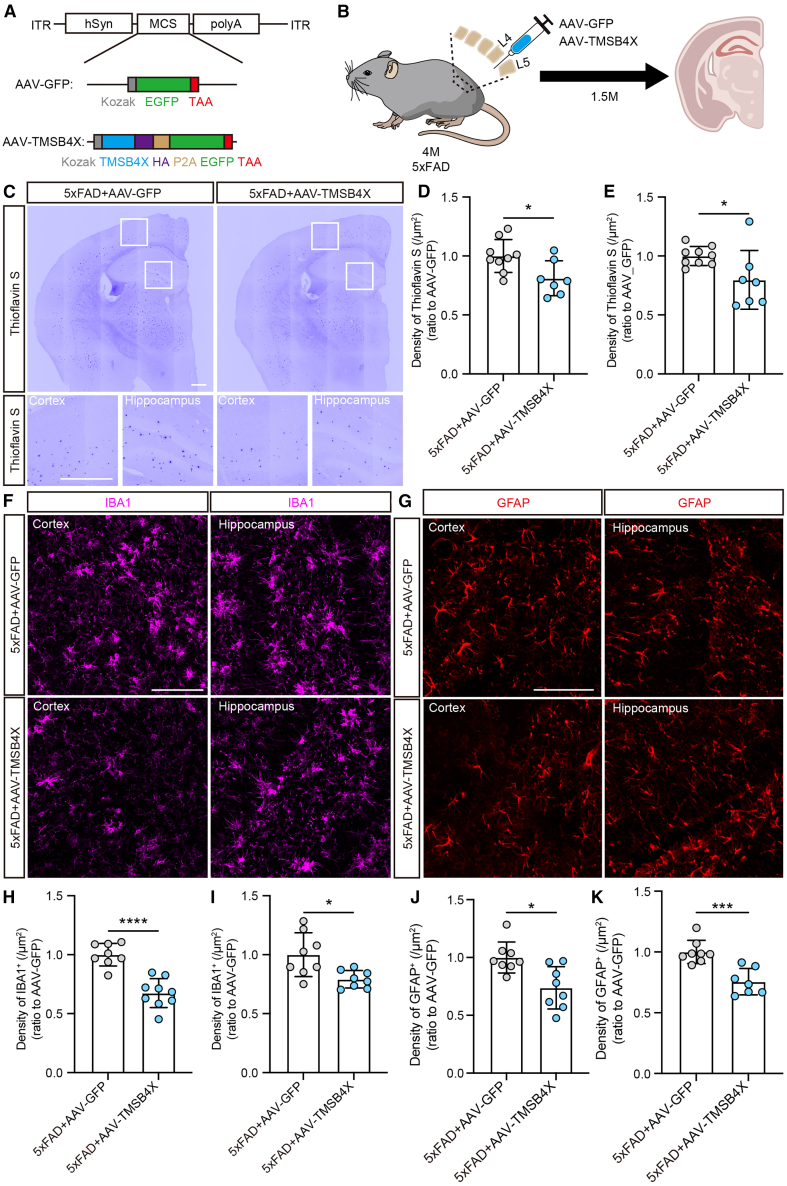
Administration of AAV-TMSB4X alleviates amyloid plaques and gliosis (A) Construct design of AAV-GFP and AAV-TMSB4X vectors, with promoter and expression elements inserted between ITRs. (B) Schematic diagram of the AAV injection in 5xFAD mice. (C) Representative images for amyloid plaques (Thioflavin S) in AAV-GFP- and AAV-TMSB4X-injected 5xFAD mice. Scale bar, 100 μm. (D and E) Quantification of the density of amyloid plaques (Thioflavin S) in the cortex (D) or hippocampus (E) of AAV-GFP- (*n* = 9 mice) and AAV-TMSB4X (*n* = 7 mice)-injected 5xFAD mice. Data are presented as mean ± SEM with the value of AAV-GFP-injected mice normalized as 1.0. Mann-Whitney test. ^∗^*p* < 0.05. (F) Immunofluorescence for IBA1-labeled microglial cells in AAV-GFP- and AAV-TMSB4X-injected 5xFAD mice. Scale bars, 100 μm. (G) Immunofluorescence for GFAP-labeled activated astrocytes in AAV-GFP- and AAV-TMSB4X-injected 5xFAD mice. Scale bars, 100 μm. (H and I) Quantification of the density of IBA1-positive cells in the cortex (H) or hippocampus (I) of AAV-GFP- and AAV-TMSB4X-injected 5xFAD mice. Data are presented as mean ± SEM of at least eight mice in each group with the value of AAV-GFP group normalized as 1.0. Mann-Whitney test. ^∗^*p* < 0.05, ^∗∗∗∗^*p* < 0.0001. (J and K) Quantification of the density of GFAP-positive cells in the cortex (J) or hippocampus (K) of AAV-GFP- and AAV-TMSB4X-injected 5xFAD mice. Data are presented as mean ± SEM of at least seven mice in each group with the value of AAV-GFP group normalized as 1.0. Mann-Whitney test. ^∗^*p* < 0.05, ^∗∗∗^*p* < 0.001. See also Figure S7.

Finally, we determined the expression and roles of *TMSB4X* in animal models *in vivo*. Notably, *Tmsb4x* expression was upregulated in the hippocampus of 5xFAD mice compared to wild-type (WT) controls (Figure S7A), consistent with prior RNA-seq data from 6- and 11-month-old 5xFAD mice (Database: GSE142633) (Figures S7B and S7C). The difference with human samples may reflect species- or cell-type-specific transcriptional adaptations to neurodegenerative stress. We constructed a plasmid with the human synapsin (hSyn) promoter to achieve overexpression of *TMSB4X* in neurons (Figure 6A) and used the AAV-PhP.eB capsid to encapsulate the plasmid and infect brain neurons of 5xFAD mice by intrathecal injection (Figure 6B). *TMSB4X* was overexpressed in the cortex and hippocampus of 5xFAD mice after virus infection for 1.5 months (Figures S7D–S7F). Consistent with the phenotypes in Tβ4-treated fAD organoids and *TMSB4X*-overexpressed APP/PS1 mice (Wang et al., 2021), overexpression of *TMSB4X* effectively inhibited the accumulation of amyloid plaques in 5xFAD mice (Figures 6C–6E, S7G, and S7H). For the soluble Aβ, injection of AAV-TMSB4X resulted in a reduction of Aβ1-40 levels and an increase in the Aβ1-42/Αβ1-40 ratio (Figures S7I–S7K). Meanwhile, the proliferation of microglia and astrocyte in AD mice was also markedly inhibited by AAV-TMSB4X injection (Figures 6F–6K). In addition to attenuating gliosis in 5xFAD brain, injection of AAV-TMSB4X also suppressed the expression of *Tnf-α* and *Il6* in the hippocampus (Figures S7L and S7M). These results indicate that overexpressing *TMSB4X* in neurons of AD mice can effectively alleviate AD pathology.

**Figure 7:**
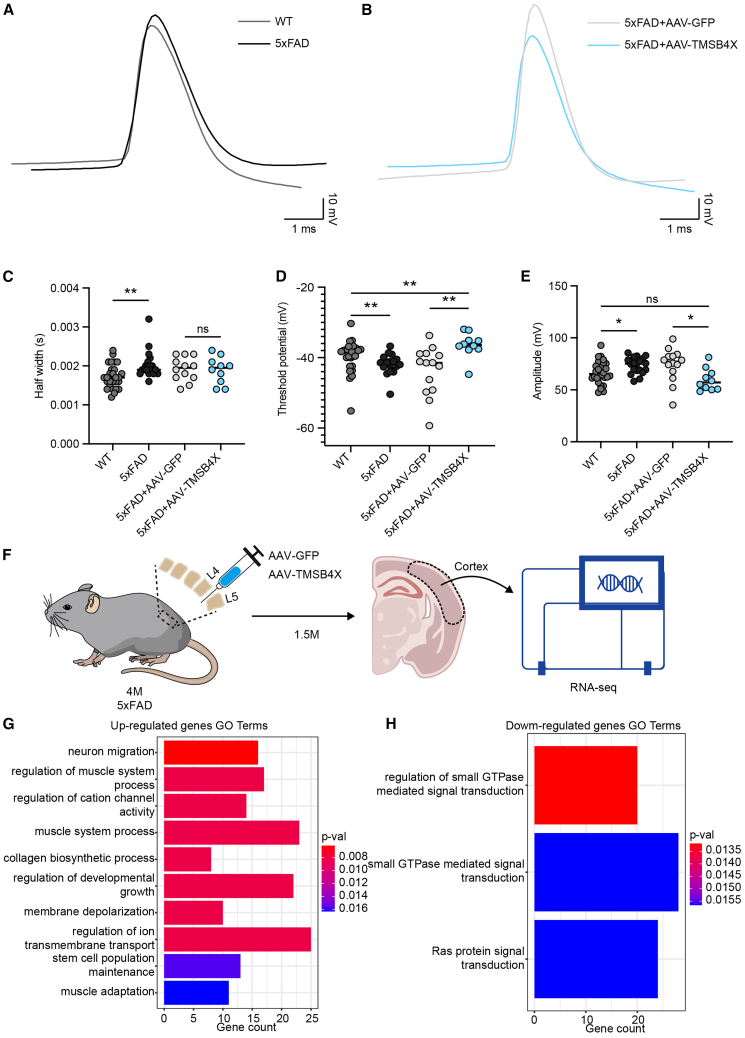
Administration of AAV-TMSB4X alleviates neuronal hyperexcitability in 5xFAD mice (A) Sample traces of action potential from excitatory neurons in the cortex of WT and 5xFAD mice. (B) Sample traces of action potential from excitatory neurons in the cortex of 5xFAD mice injected with AAV-GFP and AAV-TMSB4X, respectively. (C–E) Quantification of half-width (C), threshold potential (D), and the amplitude (E) of the action potential from excitatory neurons in indicated mice. Data are presented as mean ± SEM of at least 10 neurons from at least three mice per group. Mann-Whitney test. ^∗^*p* < 0.05, ^∗∗^*p* < 0.01. (F) Schematic diagram of the bulk RNA-seq for AAV-injected 5xFAD mice. (G and H) GO terms enriched by the upregulated genes (G) and downregulated genes (H) in AAV-injected 5xFAD mice. See also Figure S7.

In addition to common AD pathological changes, neurons in AD usually exhibit hyperexcitability (Siskova et al., 2014). In our previous results of DEGs analysis of Tβ4-treated organoids, Tβ4 was observed to affect genes related to synapse formation and neural signal transmission (Figures 5G and S6B). Thus, we determined whether overexpression of *TMSB4X* had any effects on the hyperexcitability of neurons in AD. By analyzing the action potentials (AP) of cortical excitatory neurons in 6- to 7-month-old WT and 5xFAD mice (Figure 7A), we found that excitatory neurons in 5xFAD mice showed obvious hyperexcitability characteristics including an increase in half-width, a decrease in threshold potential, and an increase in amplitude (Figures 7C–7E). Interestingly, this hyperexcitability was markedly alleviated in AD mice with *TMSB4X* overexpression (Figures 7B–7E).

To investigate the mechanism of AAV-TMSB4X in 5xFAD mice, we performed RNA-seq for 5xFAD mice injected with AAV-GFP and AAV-TMSB4X, respectively (Figure 7F). The exogenous TMSB4X-HA was detected exclusively in the AAV-TMSB4X group (Figure S7N). Differential gene expression analysis between AAV-GFP- and AAV-TMSB4X-injected 5xFAD mice identified 627 upregulated and 827 downregulated genes (Figure S7O). Principal-component analysis (PCA) further revealed distinct transcriptional profiles between AAV-GFP and AAV-TMSB4X groups (Figure S7P). GO enrichment analysis of upregulated gens revealed significant clustering in neural signaling pathways such as “membrane depolarization” and “regulation of ion transmembrane transport” (Figure 7G), potentially uncovering the mechanism by which AAV-TMSB4X reduces neuronal hyperexcitability. Conversely, downregulated genes were primarily enriched in small GTPase and Ras protein signal transduction pathways (Figure 7H). It has been shown that H-Ras deletion improves memory, reduces amyloid plaques, and protects dendrites in AD mice (Qu et al., 2023). These suggest that AAV-TMSB4X may reduce amyloid plaques production in 5xFAD mice by inhibiting the expression of Ras proteins. Taken together, *TMSB4X* has exhibited effects on AD pathology both in AD brain organoids and model mice.

## Discussion

In this study, we have used cerebral organoids to understand the cellular and molecular alterations during early neurogenesis of AD subjects. We found increased levels of Aβ in both fAD cerebral organoids at a late stage, attenuated genesis of mature neurons, and increased cell apoptosis at early stages. Furthermore, fAD organoids showed changes in the transcriptomes and developmental trajectories in a cell-type-specific manner. Additionally, we have identified *TMSB4X* as a target gene, whose downregulation contributes to impaired neurogenesis and the production of Aβ in fAD organoids. Intriguingly, overexpressing *TMSB4X* in the neurons of 5xFAD mice mitigated AD pathology and neuronal hyperexcitability.

Previous studies involving AD organoids utilizing serum samples from AD patients and iPSCs from patients with familial PSEN mutations and sporadic APOE mutations have all observed increased levels of Aβ (Chen et al., 2021; Vanova et al., 2023; Zhao et al., 2020). However, unlike these studies, we employed scRNA-seq to understand any changes in the neurogenesis and neuronal maturation of fAD organoids. After annotating the cell types of cells, mature neurons were observed to be reduced in fAD cerebral organoids. In line with this result, a previous study has shown a reduction of neurons in the cerebral organoids treated with serum from AD patients (Chen et al., 2021). The increased apoptotic signaling in AD cerebral organoids has also been observed in APOE-mutated organoids (Zhao et al., 2020).

Furthermore, we combined our single-cell RNA data with single-nuclear RNA data of AD patients (Lau et al., 2020; Morabito et al., 2021) and focused on *TMSB4X*, which showed consistent downregulation in both neurons of fAD cerebral organoids and excitatory neurons of AD patients. We found Tβ4 treatment mitigated reduced mature neurons and increased Aβ in fAD cerebral organoids. It has been shown that overexpression of Tβ4 reverses cognitive impairment in mouse model of AD via regulation of microglia polarization and inflammatory response (Wang et al., 2021). However, our result suggests that Tβ4 treatment can reduce Aβ production independent of microglia cells. This discrepancy might reflect multifaceted roles of Tβ4. Previous studies have shown that AD neurons exhibit hyperexcitability (Siskova et al., 2014), which is accompanied by abnormalities in calcium ion channels (Targa Dias Anastacio et al., 2022) and closely related to neuronal death (Sukumaran et al., 2021). Our sequencing analysis of fAD organoids treated with Tβ4 indicates that Tβ4 regulates the expression of genes related to synapse and nerve signal transmission. And overexpression of *TMSB4X* in neurons of the cortex in 5xFAD mice effectively alleviated neuronal hyperexcitability. The beneficial effects of Tβ4 in fAD organoids and animal models point to the potential as an intervention target against AD pathology.

Despite challenges in modeling irreversible neurodegenerative impacts with cerebral organoids, our comparative analysis revealed cell-type-specific early developmental alterations, which is unattainable with postmortem brain samples. Like other brain organoid studies, this study has limitations, in particular batch-to-batch and intra-batch heterogeneity. To address this issue, we applied within-batch corrections to control groups. Another limitation is the difference in cell types between organoids and human brains. To address this issue, we focused on cell types shared between organoids and the developing human brain. The third limitation is the absence of non-ectodermal cells in current cerebral organoid models, such as microglia and vascular cells, which are believed to influence disease progression (Nortley et al., 2019; Sun et al., 2023). Recently, we have established vascularized brain organoids that contain extensive amount of microglia and brain-blood barrier structures (Sun et al., 2022). This system will enable us to investigate the changes and roles of these cell types and intercellular interactions during AD progression.

## Methods

### Animals

All animal experiments, including mouse rearing, breeding, and surgical operations, were executed in compliance with the ethical guidelines of the Institutional Animal Care and Use Committee of ShanghaiTech University. For additional information on mouse husbandry and mouse strains, see supplemental methods.

### Cerebral organoids culture, treatments with Tβ4 and Aβ

Cerebral organoids were cultured following instructions of the commercial STEMdiffTM Cerebral Organoid Kit (STEMCELL #08570). For additional information on organoid generation, see supplemental methods. For Tβ4-treated cerebral organoids, the medium was replaced by Maturation Medium with the addition of Tβ4 (0.5 μg/mL, Abcam, #ab245823) at day 20 and replaced every 5 days. For Αβ-treated cerebral organoids, the medium was replaced by maturation medium with the addition of amyloid β peptide (1–42) human (5 μM, Beyotime, #P9001-1mg) at day 20 and replaced every 5 days.

### Single-cell RNA-seq

Organoids were dissociated using the methods described in a previous study (Thomsen et al., 2016). For additional information on single-cell RNA-seq and analysis, see supplemental methods.

### Bulk RNA-seq, quality control, and DEG analysis

Tβ4-treated fAD cerebral organoids and cortex from 5xFAD mice with AAV-GFP and AAV-TMSB4X injected, respectively, were sequenced on illumine Novaseq 6000 pipeline. Raw reads were mapped to the human reference genome GRCh38 (v107) by Hisat2 using default parameter. Gene expression level was calculated as count number by htseq-count software. DEG analyses were performed by edgeR (v3.38.4 R package) with *p* value <0.05 and log2 fold change >1 (Robinson et al., 2010).

### Intrathecal injection

The specific procedure for intrathecal injection follows established protocols as described previously (Cheng et al., 2023). The fur from the tail to the thoracic vertebra was shaved using a razor blade on mice anesthetized with avertin (500 mg/kg, i.p.). The AAV virus (5 × 10^11^ vg per mouse, 50 μL) was then injected into the intrathecal space between either the L4 and L5 or L3 and L4 vertebrae using a 30 G needle. The needle was removed 30 s post-injection, ensuring no fluid leakage occurred. After the injection, the mice were placed in a recovery cage and observed until normal activities resumed.

### Statistical analysis

Statistical analyses for immunostaining signals and action potentials were performed using the GraphPad Prism software v.9.5.1. Significant differences between the two groups were calculated using a two-tailed Mann-Whitney test or Student’s t test. Statistical analyses for sequencing data were performed using R software. Statistical significance was set at *p* < 0.05. Appropriate methods are indicated within the legends, and significant differences are marked in all figures. Values and error bars are presented as mean ± SEM as noted in the figure legends.

## Resource availability

### Lead contact

Requests for further information may be directed to the lead contact, Zhen-Ge Luo (luozhg@shanghaitech.edu.cn).

### Materials availability

Reagents reported in this paper is available from the lead contact upon request.

### Data and code availability

The accession numbers for single-cell RNA sequencing transcriptome data and bulk RNA-seq data supporting this study are: SRP510960 (scRNA-seq), SRP514285 (bulk RNA-seq for organoids), and SRP593290 (bulk RNA-seq for 5xFAD mice), which can be accessed at https://www.ncbi.nlm.nih.gov/sra. This paper does not report original code.

## Acknowledgments

This work was partially supported by 10.13039/501100012166National Key Research and Development Program of China (2024YFA1108000, 2021ZD0202500), 10.13039/501100001809National Natural Science Foundation of China (32130035 and 92168107), The Joint Project of the Yangtze River Delta Science and Technology Innovation Community (2024CSJZN0600), Central Guidance on Local Science and Technology Development Fund (YDZX20233100001002), Shanghai Clinical Research and Trail Center, and Shanghai Frontiers Science Center for Biomacromolecules and Precision Medicine at 10.13039/501100012600ShanghaiTech University. We thank the MultiOmics Core Facility, Molecular Imaging Core Facility, Molecular and Cell Biology Core Facility, and model animal facility at the School of Life Science and Technology, ShanghaiTech University, for providing technical support.

## Author contributions

P.-M.Z., X.-Y.S., X.-C.J., and Z.-G.L. designed the research. P.-M.Z. performed most of the experiments and prepared the figures. X.-Y.S., Y.L., and W.-D.W. provided assistance for organoids experiments. J.H. and P.-J.Q. provided assistance for mouse experiments. D.D.C. performed electrophysiology recoding. P.-M.Z. and Z.-G.L. wrote the manuscript. Z.-G.L. supervised the whole project.

## Declaration of interests

A related patent pertaining to the application of TMSB4X on AD treatment has been filed (patent applicant: ShanghaiTech University, inventors: Z.-G.L. and P.-M.Z., status: pending).

## Supplemental information

Document S1. Figures S1–S7, Table S1, and supplemental methods

Document S2. Article plus supplemental information
