# Supplementary material for: Thymosin beta 4 as an Alzheimer disease intervention target identified using human brain organoids
Source: Stem Cell Reports. 2025 Aug 14;20(9):102601. doi: 10.1016/j.stemcr.2025.102601 (PMC12447315; doi:10.1016/j.stemcr.2025.102601)
Supplement: Document S1. Figures S1–S7, Table S1, and supplemental methods [file mmc1.pdf]

Stem Cell Reports, Volume *20*

Supplemental Information

Thymosin beta 4 as an Alzheimer disease intervention target identified using human brain organoids

Peng-Ming Zeng, Xin-Yao Sun, Yang Li, Wen-di Wu, Jing Huang, Dong-Dong Cao, Pin-jue Qian, Xiang-Chun Ju, and Zhen-Ge Luo

Supplemental Figures and Legends

Figure S1

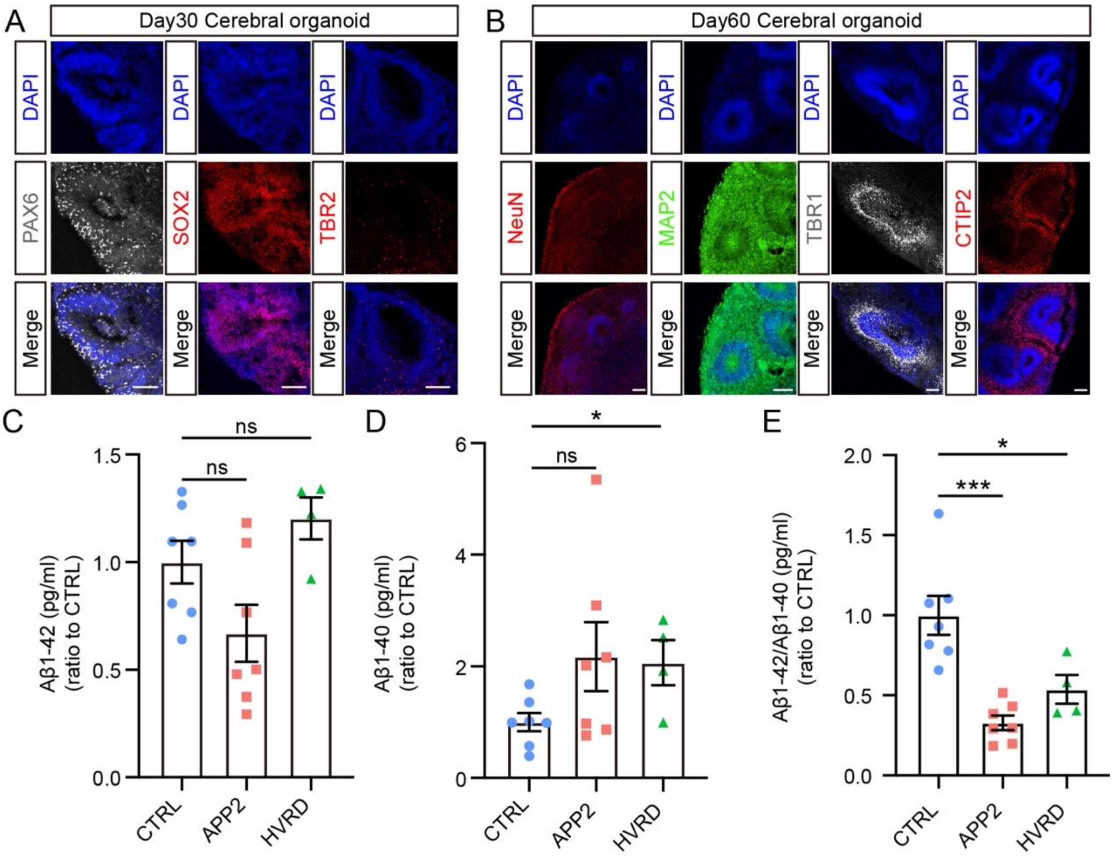

Figure S1. The analysis of iPSC-derived cerebral organoids, related to Figure 1. (A) Representative images of D30 organoids showing the expression of neural progenitor markers, including PAX6, SOX2, and TBR2. Scale bar, 100 μm. (B) Representative images of D60 organoids showing the expression of markers of differentiated neurons including NeuN, MAP2, TBR1, and CTIP2. Scale bar, 100 μm. (C) ELISA results of Aβ1-42 in the medium of D60 cerebral organoids. (D) ELISA results of Aβ1-40 in the medium of D60 cerebral organoids. (E) Ratios of Aβ1-42/Aβ1-40 from ELISA. Data are presented as mean ± SEM of 4-7 organoids per group. The value of the control group was normalized as 1.0. Mann-Whitney test. *P < 0.05, ***P < 0.001.

Figure S2

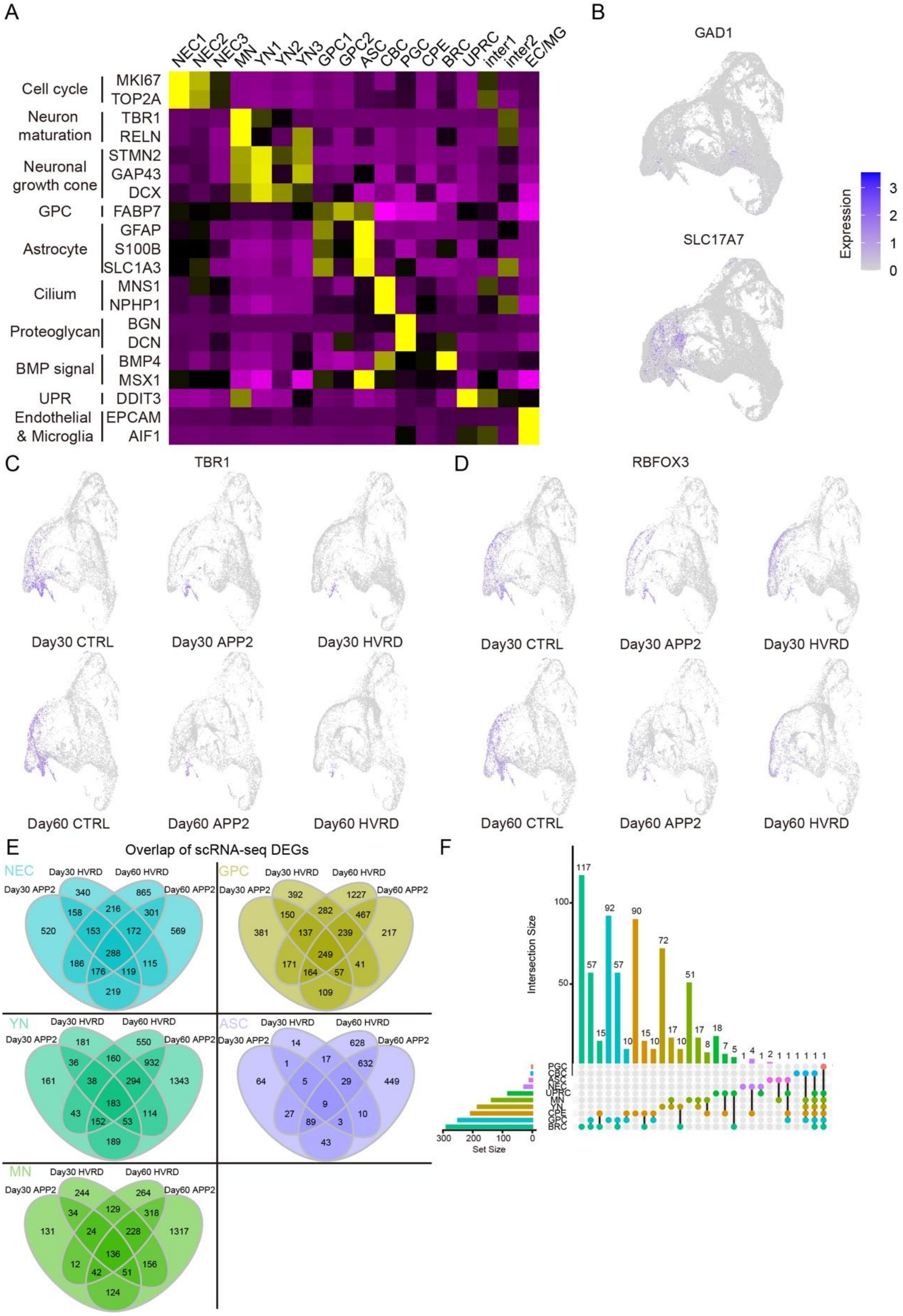

Figure S2. scRNA-seq analysis of cerebral organoids, related to Figure 1. (A)

Single-cell gene expression heat map of cell-type marker genes. (B) Expression patterns of markers for inhibitory neuron (*GAD1*) and excitatory neuron (*SLC17A7*). (C) Expression patterns of marker for sixth layer neuronal marker *TBR1* in indicated cerebral organoids. (D) Expression patterns of mature neuron marker NeuN (*RBFOX3*) in indicated organoids. (E) Venn diagrams for differential expressed genes detected in 5 major cell types (NEC: neuroepithelial cell; GPC: glia progenitor cell; YN: young neuron; ASC: astrocyte; MN: mature neuron) showing the overlaps between four fAD cerebral organoids (p.adj < 0.05). (F) Upset plot showing the overlaps between the set of differentially expressed genes identified in indicated cell types (p.adj < 0.05). CPE: choroid plexus epithelial; UPRC: unfolded-protein-response-related cell; BRC: BMP-related cell; CBC: Cilia-bearing cell; PGC: proteoglycan-expressing cell.

Figure S3

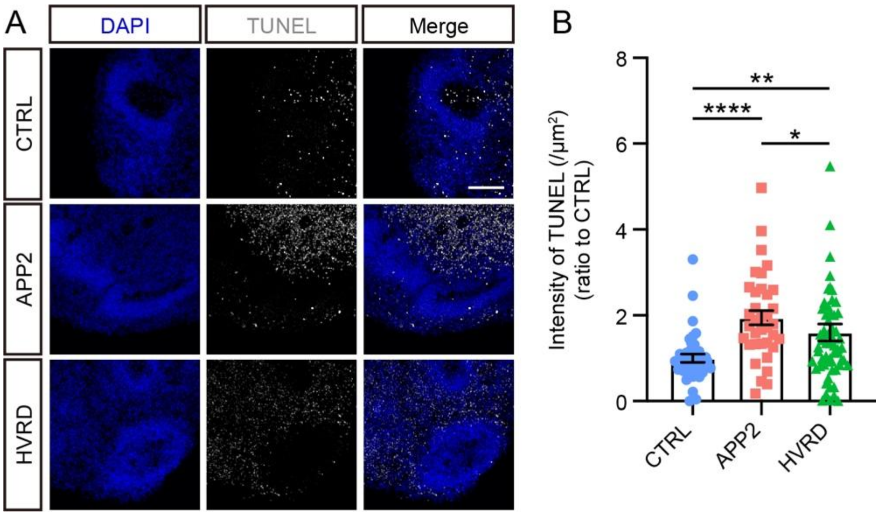

Figure S3. Cell death increases in fAD cerebral organoids, related to Figure 2. (A) Immunofluorescence for apoptosis signal (TUNEL) in D60 cerebaral organoids. Scale bar, 100 μm. (B) Quantification of the intensity of TUNEL signal in D60 cerebral organoids. Data are presented as mean ± SEM of at least 12 organoids (3 fields per organoid) per group from at least 2 independent experiments, with the value of control group normalized as 1.0. Mann-Whitney test. *P < 0.05, **P < 0.01, ****P < 0.0001.

Figure S4

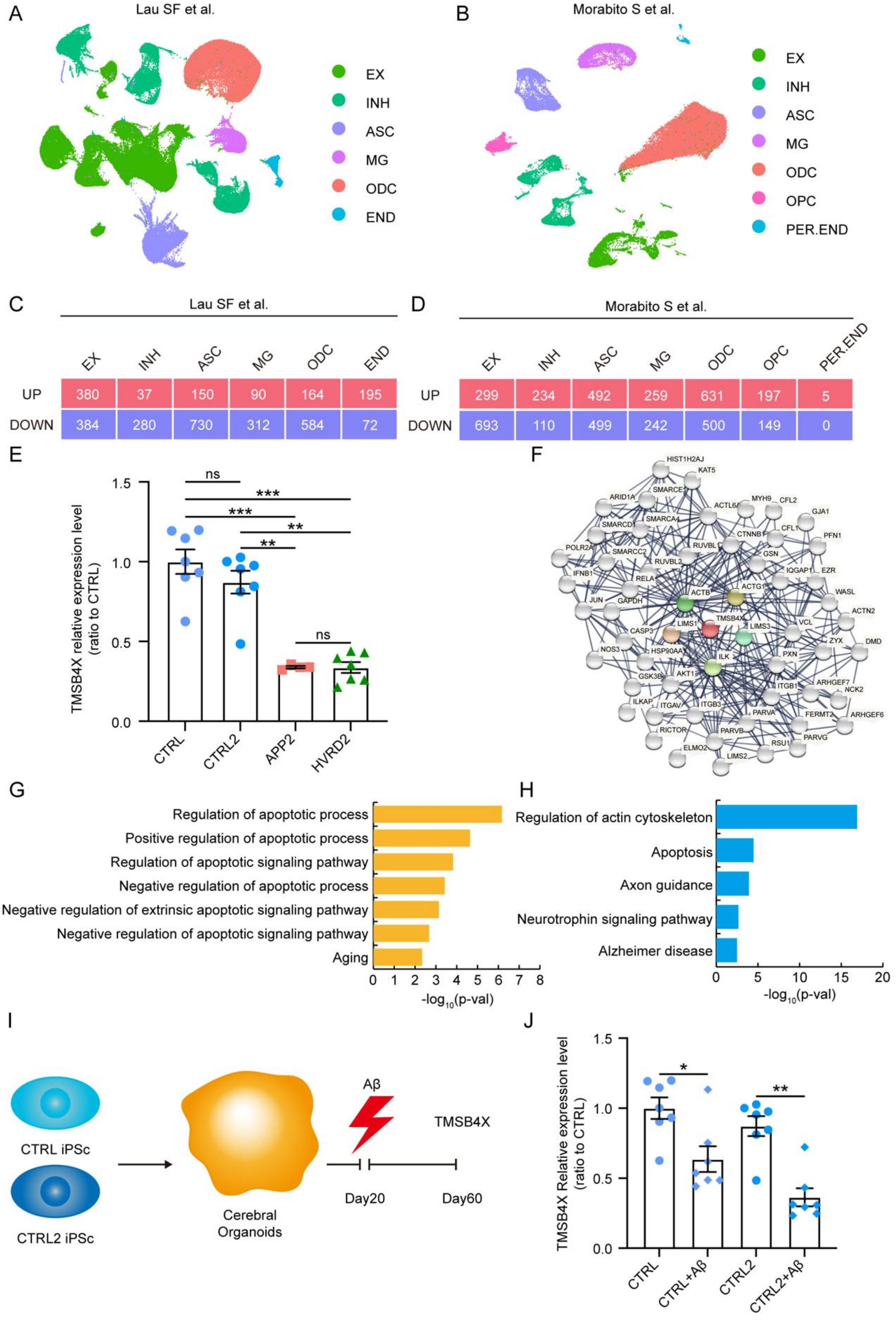

Figure S4. TMSB4X is related to aging and Alzheimer's disease pathways,

related to Figure 4. (A, B) UMAP visualization for major cell types identified by single-nuclei RNA-seq (snRNA-seq) analysis of AD brain samples. EX: excitatory neuron; INH: inhibitory neuron; ASC: astrocyte; MG: microglia; ODC: oligodendrocyte; OPC: oligodendrocyte progenitor; END: endothelial; PER.END: pericytes endothelial. (C, D) The number of differentially expressed genes identified in major cell types of AD brain samples. (p.adj <0.05). (E) The expression level of *TMSB4X* in D60 cerebral organoids. Data are presented as mean ± SEM of 4-7 organoids per group. The value of the control group was normalized as 1.0. Student's t-test. $^{**}P < 0.01$, $^{***}P < 0.001$. (F) Protein-protein interaction network for *TMSB4X* analyzed using the data from STRING database. (G) The top 7 GO terms enriched by *TMSB4X* interacting genes. (H) The top 5 KEGG pathways enriched by *TMSB4X* interacting genes. (I) Schematic representation of Aβ treatment of cerebral organoids and timeline of the analysis. (J) The expression level of *TMSB4X* in Aβ-treated cerebral organoids at D60. Data are presented as mean ± SEM of 4-7 organoids per group. The value of the control group was normalized as 1.0. Student's t-test. $^{*}P < 0.05$, $^{**}P < 0.01$.

Figure S5

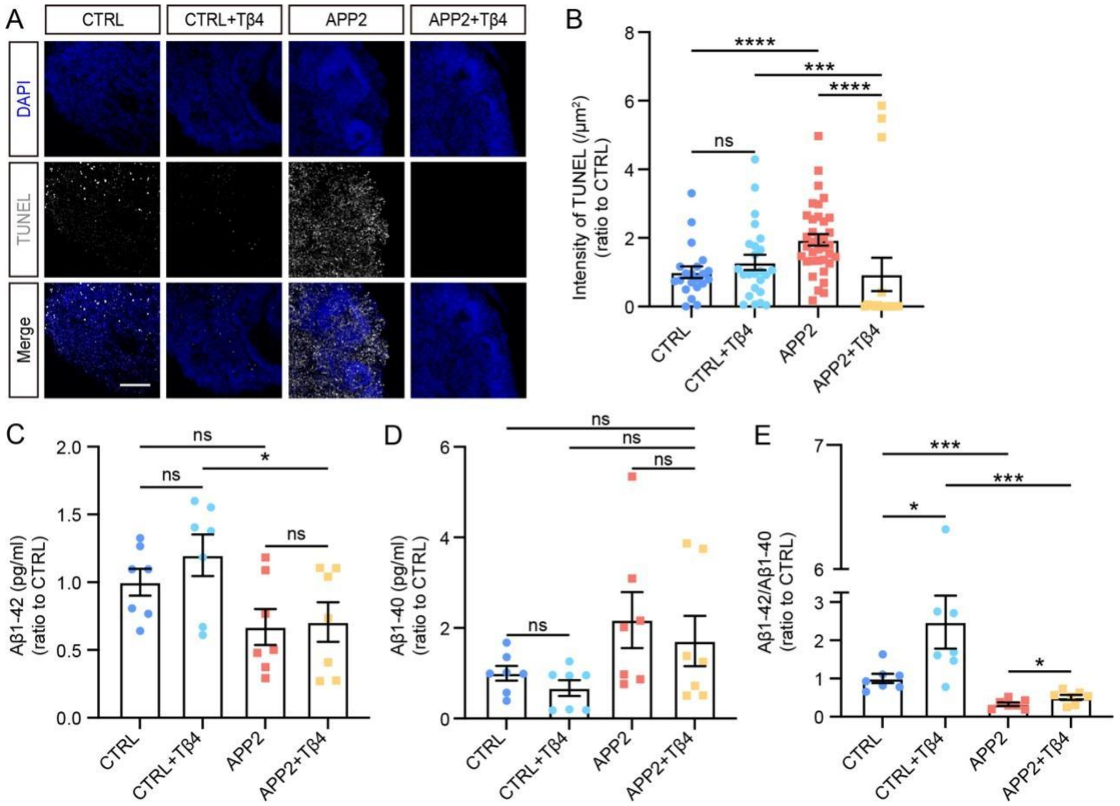

Figure S5. Thymosin β4 increases the ratios of Aβ1-42/Aβ1-40 and decreases cell death in fAD cerebral organoids, related to Figure 5. (A) Immunofluorescence for apoptosis signal (TUNEL) in D60 cerebral organoids. Scale bar, 100 μm. (B) Quantification of the intensity of TUNEL signal in D60 cerebral organoids. Data are presented as mean ± SEM of at least 6 organoids (3 fields per organoid) per group, with the value of control group normalized as 1.0. Mann-Whitney test. ***P < 0.001, ****P < 0.0001. (C) ELISA results of Aβ1-42 in the medium of D60 cerebral organoids. (D) ELISA results of Aβ1-40 in the medium of D60 cerebral organoids. (E) Ratios of Aβ1-42/Aβ1-40 from ELISA. Data are presented as mean ± SEM of 7 organoids per group. The value of the control group was normalized as 1.0. Mann-Whitney test. *P < 0.05, ***P < 0.001.

Figure S6

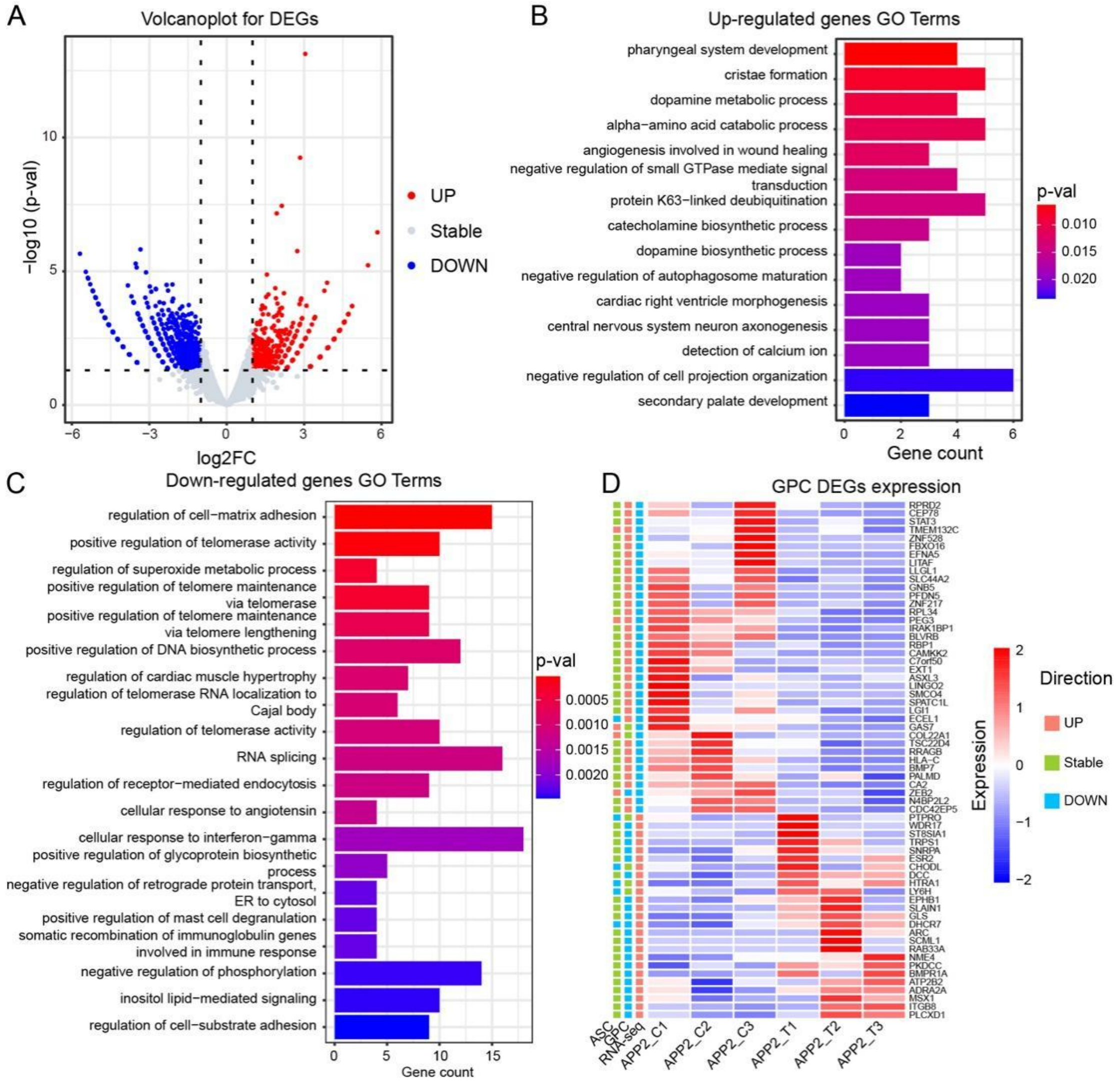

Figure S6. Bulk RNA-seq for thymosin β4-treated fAD cerebral organoids, related to Figure 5. (A) Volcano plot showing the number of differential expressed genes identified in thymosin β4 treated APP2 cerebral organoids. (B) The top 15 GO terms enriched by the up-regulated genes in thymosin β4 treated APP2 cerebral organoids. (C) The top 20 GO terms enriched by the down-regulated genes in thymosin β4 treated APP2 cerebral organoids. (D) Heatmap showing the differential expressed genes identified in thymosin β4 treated D30 APP2 cerebral organoids with different alternations in astrocyte and glia progenitor.

Figure S7

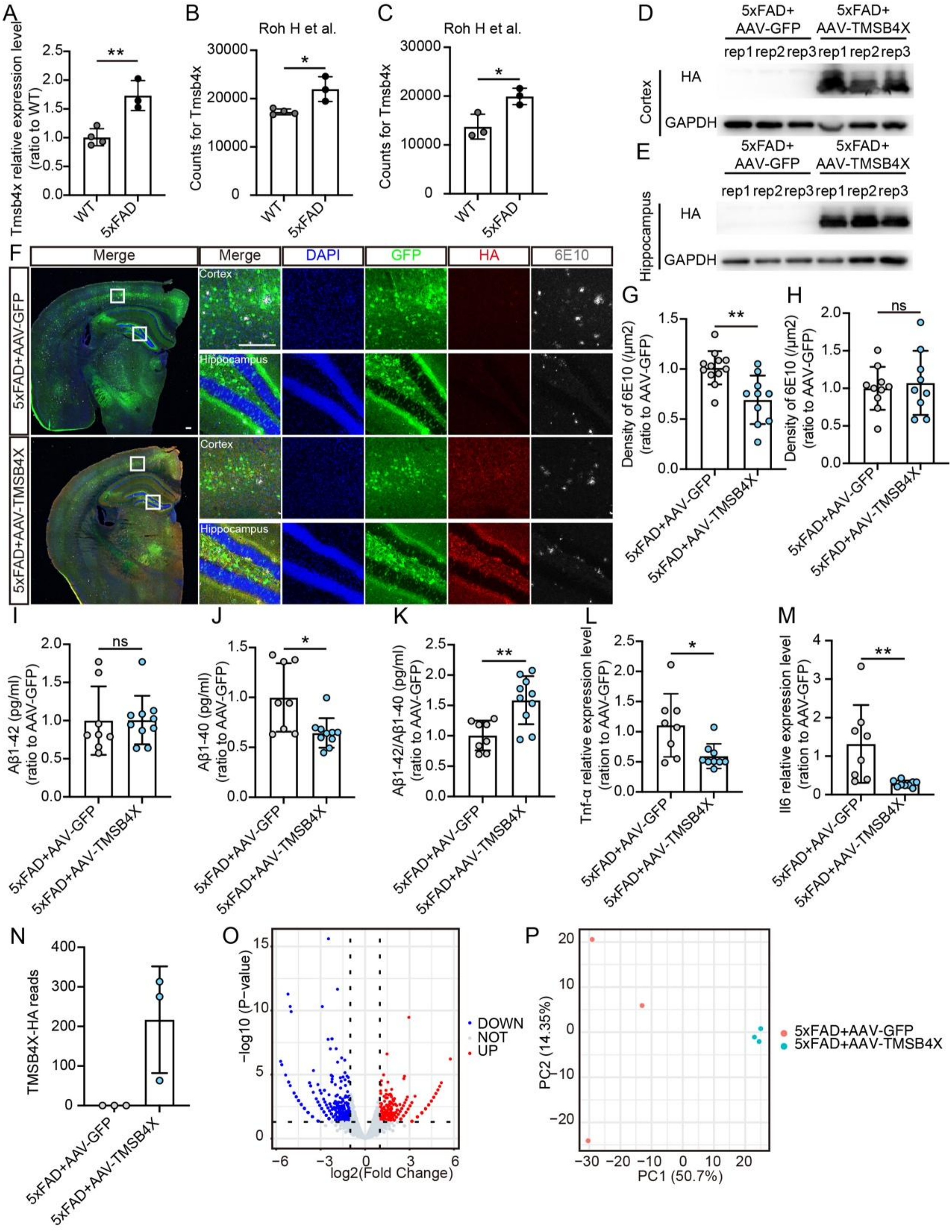

Figure S7. Administration of AAV-TMSB4X reduces amyloid plaques and neuroinflammation in 5xFAD mice, related to Figure 6 and Figure 7. (A) The expression level of *Tmsb4x* in hippocampus of WT and 5xFAD mice. Data are presented as mean ± SEM of at least 3 mice in each group with the value of WT mice

normalized as 1.0. Student's t-test. **P < 0.01. (B, C) The expression of *Tmsb4x* in bulk RNA-seq from 5xFAD mice at 6 months (B) or 11 months (C). Data are presented as mean ± SEM of at least 3 mice in each group with the value of WT mice normalized as 1.0. t test. *P < 0.05. (D, E) The expression of TMSB4X-HA in the cortex (D) or hippocampus (E) of AAV injected 5xFAD mice. Three mice were analyzed in each group (rep 1-3). (F) Immunofluorescence for GFP, HA and amyloid plaques (6E10) in AAV-GFP and AAV-TMSB4X injected 5xFAD mice. Scale bar, 100 μm. (G, H) Quantification of the density of amyloid plaques (6E10) in the cortex (G) or hippocampus (H) of AAV-GFP and AAV-TMSB4X injected 5xFAD mice. Data are presented as mean ± SEM of at least 9 mice in each group with the value of AAV-GFP group normalized as 1.0. Mann-Whitney test. **P < 0.01. (I) ELISA results of Aβ1-42 in in AAV-GFP and AAV-TMSB4X injected 5xFAD mice. (J) ELISA results of Aβ1-40 in AAV-GFP and AAV-TMSB4X injected 5xFAD mice. (K) Ratios of Aβ1-42/Aβ1-40 from ELISA. Data are presented as mean ± SEM of 7 mice per group. The value of AAV-GFP group was normalized as 1.0. Mann-Whitney test. *P < 0.05, **P < 0.01. (L) The expression level of *Tnf-α* in hippocampus of AAV-GFP and AAV-TMSB4X injected 5xFAD mice. Data are presented as mean ± SEM of at least 8 mice in each group with the value of AAV-GFP group normalized as 1.0. Student's t test. *P < 0.05. (M) The expression level of *Il6* in hippocampus of AAV-GFP and AAV-TMSB4X injected 5xFAD mice. Data are presented as mean ± SEM of at least 8 mice in each group with the value of AAV-GFP group normalized as 1.0. Student's t test. **P < 0.01. (N) The number of TMSB4X-HA reads in AAV-GFP and AAV-TMSB4X injected 5xFAD mice. (O) Volcano plot showing the number of differential expressed genes identified in AAV-TMSB4X injected 5xFAD mice compared to AAV-GFP injected 5xFAD mice. (P) PCA plot for AAV-GFP and AAV-TMSB4X injected 5xFAD mice.

Supplemental Table

Table S1. Information about the iPSCs used in this study, related to Figure 1 and Figure S4.

Cell Line	Cell Type	Disease	Gender	Age	Mutation	APOE genotype	Label
UCSD0931i-1-11	Human iPSC	None reported	Female	34	normal	APOE3/APOE3	CTRL
UCSD224i-NDC1-2	Human iPSC	None reported	Male	86	normal	APOE3/APOE3	CTRL2
HVRDi002-A-1	Human iPSC	Familial Alzheimer's disease	Female	33	APPV717I/- mutation	APOE3/APOE3	HVRD
HVRDi001-A-1	Human iPSC	Familial Alzheimer's disease	Male	57	APPV717I/- mutation	APOE3/APOE3	HVRD2
UCSD239i-App2-1	Human iPSC	Familial Alzheimer's disease	Female	60	APP (duplication)	APOE3/APOE3	APP2

Supplemental Methods

Animals

The mice were group-housed (3 to 5 mice per cage) in the institutional animal care facility, with a stable environmental temperature of 21 °C ± 1 °C, a humidity level controlled at 50% ± 10%, and a regular 12-hr light-dark cycle implemented. The 5xFAD mice (B6SJL-Tg [APP-SwFlLon, PS1*M146L*L286V] 6799Vas/J, JAX stock number: 034840) were crossed with C57 mice (C57BL/6, JAX stock number: 000664) to generate offsprings, with the day of birth being defined as postnatal day 0 (P0). Both female and male mice were incorporated into the experiments and involved in the result-recording processes.

iPSC culture

The iPSCs used in this study were purchased from WiCell company and were tested previously (Israel et al., 2012). The detailed information was showed in Supplementary Table S1. Cells were cultured on hESC-Matrigel (BD-Biocoat, #354277) coated dishes in mTeSR Plus (STEMCELL, #85850) medium with the addition of bFGF (STEMCELL, #78003) (4 μg/ml). The culture medium was half-replaced every day and then cells were passaged every 5 days by passage reagent ReLeSR (STEMCELL, #05872). The cells were tested for mycoplasma and sterility before each passage.

Cerebral organoid culture

Cerebral organoids were cultured following instructions of the commercial STEMdiffTM Cerebral Organoid Kit (STEMCELL #08570). On day 0, iPSCs colonies were dissociated into single-cell suspension using Accutase and around 9000 cells were seeded into Lipidure (#CM5206)-coated V-bottom well of ultra-low-attachment 96-well plate by 100 μl EB Formation Medium containing 1 μM Y27632 (STEMCELL, #72304). On day 2 and day 4, 100 μl EB Formation Medium without Y27632 was added into each well, respectively. On day 5, 200 μl Induction

Medium after aspirating the EB Formation Medium was added. On day 7, each EB (embryonic body) was embedded into 15 μl of Matrigel (BD-Biocoat, #354230) and cultured in 10-cm dishes with 5 ml Expansion Medium for 3 days for organoid formation. On day 10, cerebral organoids were moved into T25 flask with 15 ml Maturation Medium on a shaker. The medium was replaced every 3-4 days.

Single-cell RNA-seq

Organoids were dissociated using the methods as described in previous study (Thomsen et al., 2016). Briefly, 8-10 organoids were pooled and washed in DPBS (Life/Invitrogen, #14190144), then cut into small pieces, which were then incubated with 2 ml trypsin solution (Ca_2^+/Mg_2^+-free HBSS with 10 mM HEPES, 2 mM $MgCl_2$, 10 μg/ml DNase I, 0.25 mg/ml trypsin) for 30 min at 37°C, quenched with 4 ml Quenching Buffer. Following is the formulation of Quenching Buffer: 440ml Leibovitz L-15 medium (Thermo, #11415064), 50 ml ddH_2O, 5 ml 1M HEPES (PH 7.3-7.4), 10 μg/ml DNase I, 100 nM TTX (TOCRIS, #1069), 20 μM DNQX (TOCRIS, #0189), 50 μM DL-AP5 (TOCRIS, #3693), 5 ml 100x Anti-Anti, 2 mg/ml BSA, 100 μg/ml trypsin inhibitor (Sigma-Aldrich, #T6522). The dissociated cells were precipitated by centrifugation (220×g for 4 min at 4°C), resuspended with 2 ml Staining Medium (440ml Leibovitz L-15 medium with 50 ml ddH_2O, 5ml 1M HEPES (PH 7.3-7.4), 1g BSA, 100 nM TTX, 20 μM DNQX, and 50 μM DL-AP5, 5 ml 100 x Anti-Anti, 20 ml 77.7 mM EDTA (PH 8.0)), filtered through a 40-micron cell filter, and centrifuged again (220×g for 4 min at 4°C). Then the cell pellets were resuspended in 5 ml DPBS with 1% BSA. Dissociated cells were resuspended at a concentration of 500 cells/μl. cDNA libraries were generated by Novogene company using Single Cell Reagent Kits (10x GENOMICS), with individual cell and transcript barcoded.

Single-cell RNA sequence processing and integration

Raw data of gene expression obtained with 10x Genomics was processed using CellRanger software (v6.1.2), with human genome data version GRCh38 (v107) as

reference, generating datasets including matrix, features and barcodes information. Seurat (v4.2.0 R package) was used for quality control and integrating the single-cell transcriptome data (Macosko et al., 2015). The function called “Read10X” was used to load 10x Genomics data to R environment. For quality control, the cells with less than 300 or more than 7500 detected genes and with mitochondrial gene proportion higher than 20% were excluded. Filtered data were normalized with LogNormalize using “NormalizeData” function with default parameters and combined by “IntegratedData” function using top 20 anchors based on 2000 genes with the highest residual variance. Finally, we obtained 17644 features and 54130 cells.

Cell type dimensionality reduction and cluster analysis

For the combined data, we scaled the data by “ScaleData” function with default parameters and performed principal component analysis (PCA) based on 2000 genes with the highest residual variance. The clustering and dimensional reduction for each cell were conducted with the Seurat functions with default parameters using the top 20 principal components and 0.3 resolution. The marker genes for each cluster were identified with >0.25 average log2 fold change and < 0.1 adjusted p-value. Over-represented GO terms for these marker genes were identified by clusterProfiler (v4.4.4 R package) and org.Hs.eg.db (v3.15.0 R package) (Kuleshov et al., 2016; Wu et al., 2021).

Clusters were classified following the rules as described (Tanaka et al., 2020). Firstly, the clusters were separated by general neural genes (*SIMN2* and *DCX*) and early neurogenesis markers (*VIM, HES1, SOX2*) into neuronal and non-neuronal group. The neuronal groups were classified into mature neuron (MN) and young neuron (YN) by *YBR1*. For the non-neuronal groups, proteoglycan-expressing cell (PGC) was identified by *BGN* and *DCN*, and Cilia-bearing cell (CBC), BMP-related cell (BRC) and unfolded-protein-response-related cell (UPRC) were identified by GO terms ‘‘cilium assembly (GO:0044458)’’, ‘‘response to BMP (GO:0030509)’’ and ‘‘endoplasmic reticulum unfolded protein response (GO:0030968)’’. Neuroepithelial

cell (NEC) was identified by cell cycle-related genes such as *MKI67*. Glial progenitor cells were classified by “glial cell differentiation (GO:0010001)”. Astrocyte (ASC) were identified by *GFAP* and *SLC1A3*. Choroid plexus epithelial (CPE) was identified by TTR. Endothelia cell/microglia (EC/MG) were identified by EPCAM and AIF1. The remaining cells were distinguished as intermediate (inter).

Single-cell data comparison of control and fAD cerebral organoids

To analyze the cell-type-specific DEGs between control and fAD cerebral organoids, we calculated the significance of difference by wilcox rank sum testing. The genes with p-value < 0.05 and log2 fold change > 0.1 were identified as cell-type-specific DEGs. To identify biological processes associated with these DEGs, we used the enrichR (v3.2 R package) to query enriched GO terms and KEGG pathways for these cell-type-specific DEGs in the GO Biological Processes 2021, GO Cellular Component 2021, GO Molecular Function 2021 and KEGG 2019 Human.

Trajectory analysis

The pseudotime trajectory analysis followed the protocol as described in monocle (v2.24.1 R package) website (Cao et al., 2019; Qiu et al., 2017; Trapnell et al., 2014). Briefly, the data were extracted from Seurat analysis processing and monocle object was built by feature data, expression data and meta data. After calculating size factors and dispersions, 2000 genes with the highest residual variance were used for downstream analysis. Finally, the results were obtained by running “reduceDiemsion” and “orderCells”

Gene module score analysis

Gene module scores for particular gene sets were computed by Seurat “AddModuleScore” function. The particular gene lists used in our study include: GFAP ^{high} signature (*GFAP, ID3, AQP4, MYOC, ID1, FABP7*); GFAP^{low} signature (*LUZP2, SLC7A10, MFGE8*); Disease associated astrocyte (DAA) signature (*GFAP, CSTB, VIM, OSMR, GSN*); Up in YN signature (upregulated DEGs in young neurons

of four fAD organoids); Down in YN signature (downregulated DEGs in young neurons of four fAD organoids); Up in MN signature (upregulated DEGs in mature neurons of four fAD organoids); Down in MN signature (downregulated DEGs in mature neurons of four fAD organoids); Up in ASC signature (upregulated DEGs in astrocytes of four fAD organoids); Down in ASC signature (downregulated DEGs in astrocytes of four fAD organoids); Neuron state 1 signature (marker genes of state 1 in trajectory analysis of young neuron and mature neuron in cerebral organoids); Neuron state 7 signature (marker genes of state 7 in trajectory analysis of young neuron and mature neuron in cerebral organoids); Astrocyte state 5 signature (marker genes of state 1 in trajectory analysis of astrocyte and glia progenitor cell in cerebral organoids); Astrocyte state 3 signature (marker genes of state 3 in trajectory analysis of astrocyte and glia progenitor cell in cerebral organoids).

Immunofluorescence

The cerebral organoids were fixed in 4% paraformaldehyde (PFA) for 30-60 min after 3 washes with PBS, dehydrated with 30% sucrose in PBS overnight at 4 °C, embedded into optical cutting temperature (OCT) (Sakura, #4583) and frozen in -20 °C and cryosectioned into 35 μm-thick slides, which were then permeabilized in 0.5% Triton (Sigma-Aldrich, #T8787) and blocked with 5% BSA (Sigma-Aldrich, #V900933) in 0.1% Triton for 1 hr. Then, slides were incubated with primary antibodies in 5% BSA for two nights at 4 °C, washed with PBS three times, and incubated with Fluor-conjugated secondary antibodies overnight at 4 °C. Stained slides sections were mounted with mounting medium after washing three times with PBS. For mouse brain sections, the following procedures were carried out. Firstly, the animals were perfused with PBS. Then, the brains were fixed in 4% PFA at 4 °C for two nights, followed by dehydration in 30% sucrose dissolved in PBS at 4 °C for another two nights. The dehydrated brains were embedded in OCT compound and then frozen at -20 °C, and then cryosectioned with a thickness of 50 μm. After permeabilization in 0.5% Triton for 1 hr and blocking with 5% BSA in 0.1% Triton for 1 hr, brain sections were incubated with primary antibodies in 5% BSA over-night

at 4 °C and incubated with Fluor-conjugated secondary antibodies for 2 hr after washing three times with PBS. After three additional washes with PBS, the stained sections were mounted using mounting medium. Fluorescence signals were taken using laser scanning confocal microscopy and analyzed by Image J software. The primary antibodies include: β-amyloid (Cell Signaling, #8243T, 1:1000); PAX6 (R&D, #AF8150, 1:500); SOX2 (Santa Cruz, #sc-17320, 1:400); DCX (Santa Cruz, #sc-8006, 1:1000); TBR2 (R&D, #AF6166, 1:1000); TBR1 (Abcam, #ab31940, 1:1000); CTIP2 (Abcam, #ab18465, 1:1000); MAP2 (Sigma-Aldrich, #AB5622, 1:1000); NEUN (Millipore, #MAB377, 1:1000); c-CASP3 (Cell Signaling, #9661L, 1:1000); 6E10 (BioLegend, #803004, 1:1000); IBA1 (Oasis, #OB-PGP049-02, 1:1000); HA (Cell Signaling, #3724S, 1:1000); GFP (Abcam, #ab31970, 1:1000); GFAP (Oasis, #OB-PGP055, 1:1000). The secondary antibodies were Alexa Fluor 488, 555, 594, or 647-conjugated donkey anti-mouse, -rabbit, -rat, -pig or -chicken IgG (Invitrogen, all used at 1:1000 dilution).

TUNEL staining

Frozen cerebral organoids sections were washed three times with PBS for 5 min each, and then incubated with DAPI (Beyotime, #C1002, 1:1000) in 5% BSA over-night at 4 °C. Next, the sections were washed three times with PBS for 5 min each. Subsequently, the sections were stained for TUNEL using TUNEL apoptosis detection kit (Alexa Fluor 640) (Yeasen, #40308ES60).

Thioflavin S staining

Frozen mice brain sections were washed three times with PBS for 5 min each, and then incubated with 1 mg/ml Thioflavin S (Sigma-Aldrich, #T1892) for 7 min. The sections were then sequentially washed with 90%, 80% and 70% alcohol for 7 min each. Subsequently, they were washed three more times with PBS for 5 min each to remove any remaining alcohol. Finally, the sections were mounted and imaged.

ELISA for Aβ1-42 and Aβ1-40

The total amount of protein in cerebral organoids' culture medium and mouse brain tissue was measured by BCA protein quantification kit (YEASEN, #B2419081), and the protein amount was normalized based on the BCA detected results. The normalized proteins were then used to detect the Aβ1-42 and Aβ1-40 levels by the ELISA kits. Detection kits used were as follows: human Aβ1-40 (share-bio, #SB-FY6715-48T), human Aβ1-42 (share-bio, #SB-FY6716-48T), mouse Aβ1-40 (share-bio, #SB-FY6378-48T), mouse Aβ1-42(share-bio, #SB-FY6379-48T).

Quantitative PCR (qPCR)

Quantitative PCR was performed by using the Agilent Mx3000P qPCR system with the 2xSYBR Green qPCR Master Mix (Bimake). Relative mRNA expression was determined by the delta cycle time with human 18S as the internal control in data normalization for cerebral organoids and mouse β-actin as the internal control in data normalization for mouse tissue. Primer sequences were as follows:

18S: forward, 5'- ATCACCATTATGCAGAATCCACG-3', reverse, 5'-GACCTGGCTGTATTTTCCATCC-3';

TMSB4X: forward, 5'-CGAATCGTAATGAGGCGTGC-3', reverse, 5'-TCCCTGCCAGCCAGATAGAT-3';

β-actin: forward, 5'-GAGACCTTCAACACCCCAGC-3', reverse, 5'-ATGTCACGCACGATTTCCC-3';

Tmsb4x: forward, 5'-CGTCCTTAAAGCCAAGTCCAAG-3', reverse, 5'-TACAGTGCATATTGGCGGCG-3';

Tnf-α: forward, 5'-ACCCTCACACTCACAAACCA-3', reverse, 5'-ATAGCAAATCGGCTGACGGT-3';

Il6: forward, 5'-GCCTTCTTGGGACTGATGCT-3', reverse, 5'-GTGACTCCAGCTTATCTCTTGGT-3'.

Electrophysiological recording

Transverse cortical slices were prepared from 5.5–7-month-old WT, 5xFAD, AAV-GFP or AAV-TMSB4X injected 5xFAD mice. The mice were anesthetized with

avertin (625 mg/kg, i.p.) and then perfused with cold, oxygenated NMDG ACSF solution (93 mM NMDG, 93 mM HCl, 2.5 mM KCl, 1.25 mM NaH_2PO_4, 10 mM $MgSO_4 \cdot 7H2O$, 30 mM $NaHCO_3$, 25 mM glucose, 20 mM HEPES, 5 mM sodium ascorbate, 3 mM sodium pyruvate, and 2 mM thiourea). After perfusion, the brain was rapidly dissected and transferred to a cold NMDG ACSF solution, and then sectioned coronally using a vibratome (VT1200 S, Leica) in the same buffer at a thickness of 300 µm. The cortical slices were incubated in oxygenated NMDG ACSF at 32 °C for 10-15 min and subsequently transferred to a normal oxygenated ACSF solution (126 mM NaCl, 2.5 mM KCl, 1.25 mM NaH_2PO_4, 2 mM $MgSO_4 \cdot 7H_2O$, 10 mM glucose, 26 mM $NaHCO_3$, 2 mM $CaCl_2$) at room temperature for one hour. All chemicals used in the preparation of the slices were obtained from Sigma-Aldrich (St. Louis, MO, USA).

The brain slices were carefully transferred to a recording chamber that was completely submerged and continuously perfused with ACSF solution at a flow rate of 3 mL/min, and maintained at 28 °C. The neurons for recoding were visualized by differential interference contrast optics (DIC; Olympus BX61WI). The recording pipettes with 3 to 4 MΩ resistance were fabricated using a micropipette puller (P2000, Sutter Instrument; USA). For whole-cell recordings, the pipettes were filled with an ACSF solution composed of 133 mM potassium gluconate, 18 mM NaCl, 0.6 mM EGTA, 10 mM HEPES, 2 mM Mg·ATP, and 0.3 mM $NA_3 \cdot GTP$ (pH 7.2, 280 mOsm). Once a whole-cell configuration was successfully established, the neurons were voltage-clamped at -70 mV. To evoke action potentials (AP), a current-step protocol was repeatedly applied, with the current ranging from -20 to +400 pA in increments of 20 pA. Detection and analysis of AP were performed by Clampfit Program.

References

Cao, J., Spielmann, M., Qiu, X., Huang, X., Ibrahim, D.M., Hill, A.J., Zhang, F., Mundlos, S., Christiansen, L., Steemers, F.J., et al. (2019). The single-cell

transcriptional landscape of mammalian organogenesis. Nature *566*, 496-502. 10.1038/s41586-019-0969-x.

Israel, M.A., Yuan, S.H., Bardy, C., Reyna, S.M., Mu, Y., Herrera, C., Hefferan, M.P., Van Gorp, S., Nazor, K.L., Boscolo, F.S., et al. (2012). Probing sporadic and familial Alzheimer's disease using induced pluripotent stem cells. Nature *482*, 216-220. 10.1038/nature10821.

Kuleshov, M.V., Jones, M.R., Rouillard, A.D., Fernandez, N.F., Duan, Q., Wang, Z., Koplev, S., Jenkins, S.L., Jagodnik, K.M., Lachmann, A., et al. (2016). Enrichr: a comprehensive gene set enrichment analysis web server 2016 update. Nucleic Acids Res *44*, W90-97. 10.1093/nar/gkw377.

Macosko, E.Z., Basu, A., Satija, R., Nemesh, J., Shekhar, K., Goldman, M., Tirosh, I., Bialas, A.R., Kamitaki, N., Martersteck, E.M., et al. (2015). Highly Parallel Genome-wide Expression Profiling of Individual Cells Using Nanoliter Droplets. Cell *161*, 1202-1214. 10.1016/j.cell.2015.05.002.

Qiu, X., Mao, Q., Tang, Y., Wang, L., Chawla, R., Pliner, H.A., and Trapnell, C. (2017). Reversed graph embedding resolves complex single-cell trajectories. Nat Methods *14*, 979-982. 10.1038/nmeth.4402.

Tanaka, Y., Cakir, B., Xiang, Y., Sullivan, G.J., and Park, I.H. (2020). Synthetic Analyses of Single-Cell Transcriptomes from Multiple Brain Organoids and Fetal Brain. Cell Rep *30*, 1682-1689 e1683. 10.1016/j.celrep.2020.01.038.

Thomsen, E.R., Mich, J.K., Yao, Z., Hodge, R.D., Doyle, A.M., Jang, S., Shehata, S.I., Nelson, A.M., Shapovalova, N.V., Levi, B.P., and Ramanathan, S. (2016). Fixed single-cell transcriptomic characterization of human radial glial diversity. Nat Methods *13*, 87-93. 10.1038/nmeth.3629.

Trapnell, C., Cacchiarelli, D., Grimsby, J., Pokharel, P., Li, S., Morse, M., Lennon, N.J., Livak, K.J., Mikkelsen, T.S., and Rinn, J.L. (2014). The dynamics and regulators of cell fate decisions are revealed by pseudotemporal ordering of single cells. Nat Biotechnol *32*, 381-386. 10.1038/nbt.2859.

Wu, T., Hu, E., Xu, S., Chen, M., Guo, P., Dai, Z., Feng, T., Zhou, L., Tang, W., Zhan, L., et al. (2021). clusterProfiler 4.0: A universal enrichment tool for interpreting omics

data. Innovation (Camb) *2*, 100141. 10.1016/j.xinn.2021.100141.
