# Supplementary material for: Thymosin beta 4 as an Alzheimer disease intervention target identified using human brain organoids
Source: Stem Cell Reports. 2025 Aug 14;20(9):102601. doi: 10.1016/j.stemcr.2025.102601 (PMC12447315; doi:10.1016/j.stemcr.2025.102601)
Supplement: Document S2. Article plus supplemental information [file mmc2.pdf]

Stem Cell Reports

Article

OPEN ACCESS

Thymosin beta 4 as an Alzheimer disease intervention target identified using human brain organoids

Peng-Ming Zeng,[1] Xin-Yao Sun,[1,2] Yang Li,[1] Wen-di Wu,[1] Jing Huang,[1] Dong-Dong Cao,[1] Pin-jue Qian,[1] Xiang-Chun Ju,[3] and Zhen-Ge Luo[1,4,*]

[1]School of Life Science and Technology & State Key Laboratory of Advanced Medical Materials and Devices, ShanghaiTech University, Shanghai 201210, China

[2]Institute of Neuroscience, Center for Excellence in Brain Science and Intelligence Technology, Chinese Academy of Sciences, Shanghai 200031, China

[3]Okinawa Institute of Science and Technology Graduate University, Okinawa 904-0495, Japan

[4]Lead contact

*Correspondence: luozhg@shanghaitech.edu.cn

https://doi.org/10.1016/j.stemcr.2025.102601

SUMMARY

The developmental origin of Alzheimer disease (AD) has been proposed but is arguably debated. Here, we developed cerebral organoids from induced pluripotent stem cells (iPSCs) with mutations in amyloid precursor protein (APP) associated with familial AD (fAD) and analyzed the dynamic changes of cellular states. We found that mature neurons induced in fAD organoids markedly decreased compared to that of health control, accompanied with increased cell senescence and β-amyloid (Aβ) production. Interestingly, the expression level of the gene *TMSB4X* that encodes thymosin beta 4 (Tβ4) significantly decreased both in fAD organoids' neurons and AD patients' excitatory neurons. Remarkably, the neurodevelopmental deficits and Aβ formation in fAD organoids were rescued by treatment with Tβ4. The beneficial effects of Tβ4 were also revealed in 5xfAD model mice. Thus, this study has identified Tβ4 as a neuroprotective factor that may mitigate altered neurogenesis and AD pathology, highlighting a potential for disease intervention.

INTRODUCTION

Alzheimer disease (AD) is characterized by a range of cellular pathologies, including accumulation of β-amyloid peptide (Aβ), formation of neurofibrillary tangles (NFT), increased glial activation, and synaptic and neuronal loss (Hardy and Selkoe, 2002; Sato et al., 2018). In support of Aβ hypothesis, mutations in genes encoding amyloid precursor protein (APP) or subunits of γ-secretase, which cleaves APP to generate Aβ, have been found to be closely associated with AD occurrence and progression (Israel et al., 2012; Muratore et al., 2014).

It has been shown that long before the onset of AD pathology and behavior impairments, defects in brain structures and functions have been observed in AD patients (Busche and Konnerth, 2016; Crews et al., 2010). The earlier onset of neurogenesis defects preceding the core AD pathology including plaque and tangle formation has also been found in animal models harboring fAD mutations (Hamilton et al., 2010; Wen et al., 2004). Interestingly, treatment with Aβ has been shown to affect proliferation, differentiation, and survival of cultured human and rodent neural progenitor cells (Haughey et al., 2002). Thus, it is conceivable that future treatments to intervene the progression of AD may take effect when administered to risk people at early stages.

Identification of molecular targets for disease modification has relied on the analysis of human postmortem brain samples and animal models. However, the knowledge obtained using mouse models remain limited due to their developmental discrepancies compared with humans. The recently developed brain organoid model derived from induced pluripotent stem cells (iPSCs) has provided an opportunity to access the developmental process of human brain and model diseases *in vitro*, lifting the ethical issues related with limited accessibility of live human brains (Giandomenico and Lancaster, 2017). For instance, brain organoids from familial AD (fAD) iPSCs have shown AD-specific molecular features and pathology, as well as cell fate changes (Vanova et al., 2023; Zhao et al., 2020). However, these studies only focused on the description of the phenotypes in AD cerebral organoids but did not conduct further studies on specific pathogenic mechanisms, let alone the identification of intervention targets.

In this study, we generated fAD cerebral organoids from iPSCs with APP gene duplication and APPV717I mutation. The fAD cerebral organoids exhibited an increase in the level of Aβ, and alterations in cell types, as well as cell-type-specific transcriptomic changes. Furthermore, we compared the differential expression genes (DEGs) detected in fAD cerebral organoids with the single-nucleus RNA sequencing (snRNA-seq) data from AD patients and found a set of genes that showed similar tendency. We focused on functional studies on *TMSB4X*, which showed decrease both in neurons of fAD cerebral organoids and excitatory neurons of AD patients. The defects of neurogenesis and increase of Aβ in fAD cerebral organoids can be rescued by treatment with thymosin beta 4 (Tβ4), the protein product of *TMSB4X*. Overexpression of *TMSB4X* in neurons via AAV-TMSB4X rescued the pathological

Published by Elsevier Inc. on behalf of International Society for Stem Cell Research.
This is an open access article under the CC BY license (http://creativecommons.org/licenses/by/4.0/).

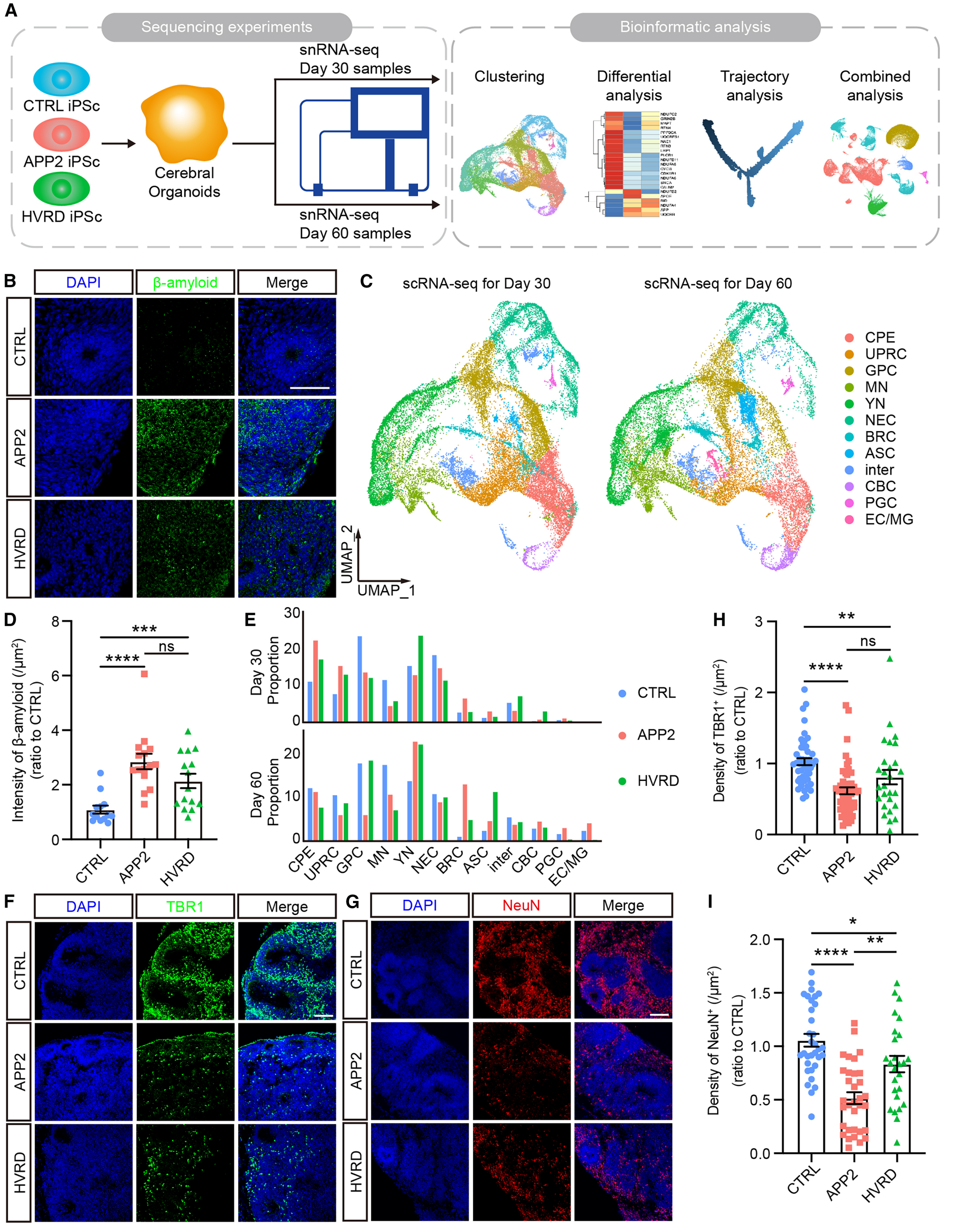

(legend on next page)

changes and mitigated neuronal hyper-excitability in 5xFAD mice. Thus, the fAD organoids developed in this study have shown early onset of neurogenesis defects and provided a platform for target screening and testing.

RESULTS

Generation and verification of fAD cerebral organoids

Three iPSCs cell lines including two fAD iPSCs and one control iPSC were used in single-cell RNA sequencing (scRNA-seq) (Table S1). The two fAD iPSCs carry *APP* gene duplication mutation (APP2) and *APPV717I* single-site mutation (HVRD), respectively, which are positively associated with AD pathology caused by increased Aβ due to higher APP level or increased processing (Israel et al., 2012; Muratore et al., 2014). The cerebral organoids were generated using the protocol as described in previous studies (Lancaster and Knoblich, 2014) and then analyzed for cell composition using scRNA-seq and immunostaining at different days (D30, D60, or D90) (Figure 1A). As expected, abundant neural progenitors (NPs) were present in D30 organoids as reflected from positive signals of stem cell marker SOX2, radial glia marker PAX6, and intermediate progenitor marker TBR2 (Figure S1A), and neurons gradually became mature later on as indicated by signals for pan-neuronal marker MAP2, mature neuron marker NeuN, and cortical layer markers TBR1 and CTIP2 (Figure S1B). We also determined levels of Aβ and found that the fAD organoid exhibited marked increase in the intensity of Aβ at D90 (Figures 1B and 1D). For the soluble Aβ, the ratio of Aβ1-42/Aβ1-40 significantly decreased in the medium of fAD organoids at D60 (Figures S1C–S1E). This finding aligns with prior studies documenting altered Aβ1-42/Aβ1-40 ratios in the cerebrospinal fluid of AD patients (Delaby et al., 2022). Thus, the fAD cerebral organoids exhibited AD-related changes after maturation.

The cell composition and heterogeneity were also revealed by scRNA-seq, which showed the presence of 12 cell types, after dimensionality reduction and cell-type clustering (Figure 1C). The annotation for these clusters was based on the expression of their marker genes and Gene Ontology (GO) terms enriched (Figure S2A; see methods). The limited *GAD1* expression and pronounced *SLC17A7* expression (Figure S2B) indicated the preference for generating excitatory neurons. It appeared that D60 organoids contained more mature neurons than D30 organoids (Figures 1C and 1E), and the proportion of several cell types between the control and fAD organoids displayed different distribution (Figure 1E). Interestingly, both APP2 and HVRD organoids showed reduction of mature neurons (MN) at 30 and 60 days and increase of young neurons (YN), BMP-related cells (BRC), and astrocytes (ASC) at 60 days, suggesting an impairment in neuronal maturation (Figure 1E). The expression of sixth layer neuron (*TBR1*) and mature neuron markers (NeuN/*RBFOX3*) showed marked reduction in both of the fAD cerebral organoids compared to the control (Figures S2C and S2D). In line with this notion, immunostaining results showed that TBR1 signal in D30 organoids or NeuN signals in D60 organoids decreased in both APP2 and HVRD organoids (Figures 1F–1I).

Cell-type-specific transcriptomic changes in fAD cerebral organoids

Then we compared transcriptome profiles of all cell types between fAD and control organoids at D30 and D60, respectively (Figure S2E). Notably, more DEGs were observed only in a certain cell type, and only limited numbers of DEGs were present across multiple cell types, indicating the cell-type-specific differences in fAD organoids (Figure S2F). Next, we analyzed biological functions of these DEGs in various cell types using the GO and Kyoto Encyclopedia of Genes and Genomes (KEGG) analysis.

Figure 1. The cell composition difference between fAD and control cerebral organoids

(A) Schematic representation of the iPSCs and organoids used in this study, sequencing experiments, and downstream bioinformatics analysis.

(B) Immunofluorescence for β-amyloid in D90 cerebral organoids. Scale bars, 100 μm.

(C) UMAP visualization of 12 major cell types isolated from D30 and D60 cerebral organoids. CPE, choroid plexus epithelial; UPRC, unfolded-protein-response-related cell; GPC, glia progenitor cell; MN, mature neuron; YN, young neuron; NEC, neuroepithelial cell; BRC, BMP-related cell; ASC, astrocyte; inter, intermediate; CBC, Cilia-bearing cell; PGC, proteoglycan-expressing cell; EC/MG, endothelia cell/microglia.

(D) Quantification of the intensity of β-amyloid in D90 cerebral organoids. Data are presented as mean ± SEM of at least 13 organoids per group from four independent experiments. Mann-Whitney test. ***$p < 0.001$, ****$p < 0.0001$.

(E) The proportions of major cell types in fAD and control cerebral organoids at D30 (up) and D60 (down).

(F) Immunofluorescence for sixth layer neuron marker TBR1 in D30 cerebral organoids. Scale bars, 100 μm.

(G) Immunofluorescence for mature neuron marker NeuN in D60 cerebral organoids. Scale bars, 100 μm.

(H and I) Quantification of the density of TBR1-positive cells in D30 organoids (H) or the density of NeuN-positive cells in D60 organoids (I). Data are presented as mean ± SEM of at least nine organoids (three fields per organoid) per group from at least three independent experiments. The value of the control group was normalized as 1.0. Mann-Whitney test. *$p < 0.05$, **$p < 0.01$, ****$p < 0.0001$. See also Figures S1 and S2 and Table S1.

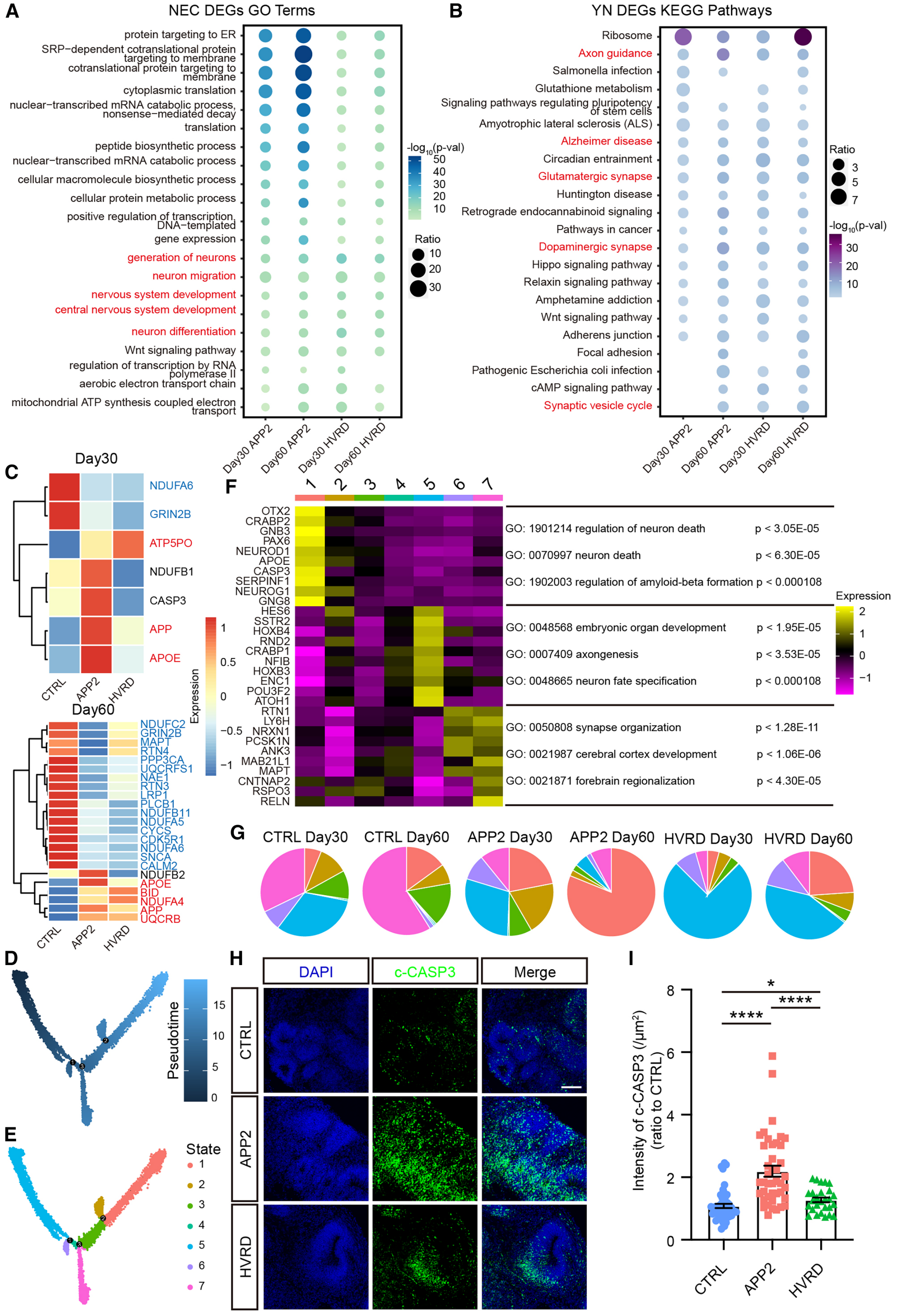

(legend on next page)

We found that DEGs in neuroepithelial cells (NECs), which represent neural stem cell populations contributing to early neurogenesis, were enriched with terms including “generation of neurons” and “neuron differentiation” (Figure 2A). Meanwhile, the DEGs in YN were enriched with “axon guidance” and “glutamatergic synapse” pathways, which are important for synapse formation in neurons, especially excitatory neurons (Figure 2B). These results agree with the fact that fAD cerebral organoids displayed neuronal maturation defects as shown in a previous study (Ghatak et al., 2021). Interestingly, the DEGs in YN were also enriched with “Alzheimer disease” pathway, suggesting that molecular features of AD already emerged in neurons at early differentiation stage (Figure 2B). We then analyzed the expression of these DEGs enriched in the “Alzheimer disease” pathway and defined them as AD-related DEGs (Figure 2C). With the increase of the culture time, the D60 fAD organoids showed more AD-related DEGs than that at D30 (Figure 2C). Both the *APP* and *APOE* genes, which are associated with a high risk of AD as well as aging and apoptosis in neurons (Wang et al., 2014; Zhao et al., 2022), were found to be highly expressed in the neurons of D30 and D60 fAD organoids. Notably, genes encoding mitochondrial membrane proteins related to aerobic electron transport chain, including *NDUFA4*, *NDUFA6*, and *NDUFB11*, were observed in D60 fAD organoids (Figure 2C). These results indicate the activation of neuronal apoptosis and mitochondrial dysfunction in early differentiated neurons in fAD organoids.

The presence of apoptotic state of differentiated neurons in fAD organoids was also confirmed by pseudotime trajectory analysis, which showed two branched neuronal differentiation pathways comprised of seven cell states, with states 1 and 7 representing two distinct terminal states (Figures 2D and 2E). GO analysis showed that genes associated with neuronal fate specification and organ development were highly expressed in the original state (state 5), genes related with Aβ formation and cell death were highly expressed in state 1, and genes associated with synapse organization and brain regionalization were highly expressed in state 7 (Figure 2F). Interestingly, fAD organoids contained more neurons at state 1 and less neurons at state 5 at D60 (Figure 2G). In line with this notion, both the signal of cleaved caspase3 (c-CASP3) and TUNEL were increased in fAD organoids (Figures 2H, 2I, S3A, and S3B). The destination to cell death state may be due to the retardation of neuronal maturation. These results imply that the accumulation of AD-like molecular and cellular features may be originated from early brain developmental stages.

In addition to neuronal cells, we also analyzed DEGs and trajectory of glia linage cells. The snRNA-seq study for the hippocampus from 5xFAD mouse have classified astrocytes into $GFAP^{low}$, $GFAP^{high}$, and disease-associated astrocyte (DAA) subtypes (Habib et al., 2020). The gene signatures in DAA, including *GSN*, *GFAP*, *CLU*, and *CD9*, were upregulated in fAD cerebral organoids, and this tendency was more remarkable at D60 compared to D30 (Figure 3A). In line with this notion, DAA-related GO terms were also enriched in ASC DEGs (Figure 3B). Unlike the results in neurons, only the ASC DEGs from HVRD organoids showed enrichment in the “Alzheimer’s disease” pathway (Figure 3D). In addition, the “PI3K-Akt signaling pathway,” which is related to astrocyte activation (Pang et al., 2023), was consistently enriched across all four fAD organoids (Figure 3D). We also determined the expression of the ASC DEGs associated with “Alzheimer disease” pathway (Figure 3C). Like in neurons, mitochondrial-related genes *COX7A2L* and *UQCRB* showed differential expression in ASC of fAD organoids (Figure 3C). Among the genes associated with AD, *UQCRB*, *RTN3*, *PLCB1*, and *RTN4* were consistently differentially expressed in both YN and ASC of fAD organoids (Figures 2C and 3C). The decreased expression of *RTN3* and *RTN4* has been shown to be associated with increased production of *BACE1*, resulting in accumulation of APP cleavage products (Murayama et al., 2006). These results indicate that astrocytes in our fAD organoids display AD-like features.

Figure 2. Enrichment of neuronal clusters with AD-like features in fAD cerebral organoids
(A) The top enriched GO terms in neuroepithelial cells of each fAD cerebral organoid.
(B) The top enriched KEGG pathways in young neurons of each fAD cerebral organoid.
(C) Heatmap for the expression of differential expressed genes, enriched in Alzheimer’s disease pathway, in young neurons at 30 and 60 days, respectively. The red color means that the gene exhibits a consistent upregulation in its differential expression within APP2 and HVRD organoids. The blue color means that the gene exhibits a consistent downregulation within APP2 and HVRD organoids.
(D and E) Differentiation pathways labeled by pseudotime (D) or cell states (E) in neurons (young neurons and mature neurons).
(F) Heatmap showing the top 10 marker genes in state 1, 5, and 7 cells in neurons with specific GO terms listed.
(G) The proportions of neuronal states in fAD and control cerebral organoids.
(H) Immunofluorescence for apoptosis signal (c-CASP3) in D60 cerebral organoids. Scale bars, 100 μm.
(I) Quantification of the intensity of c-CASP3 in D60 cerebral organoids. Data are presented as mean ± SEM of at least nine organoids (three fields per organoid) per group from at least three independent experiments, with the value of control group normalized as 1.0. Mann-Whitney test. *$p < 0.05$, ****$p < 0.0001$. See also Figure S3.

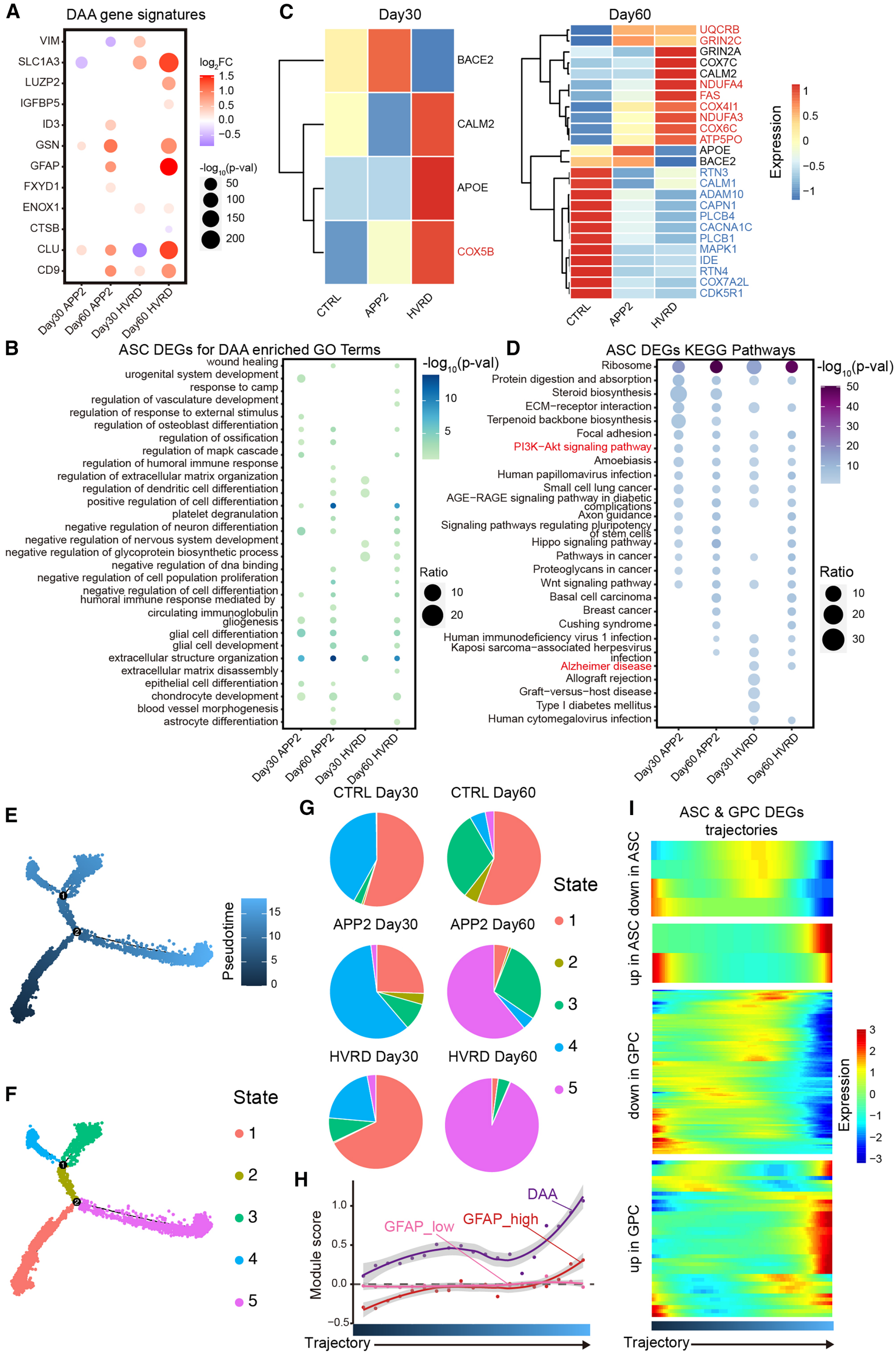

(legend on next page)

The visualization of glia progenitor cells (GPCs) and ASC subclasses and pseudotime trajectory analysis showed starting (state 1) and distinct final states (states 3 and 5) of astrocyte (Figures 3E and 3F). In D30 organoids, the distribution of cells at different states displayed big discrepancy. However, in D60 organoids, the APP2 and HVRD groups exhibited common increase in the proportion of cells at state 5 and a decrease of cells at state 2 (Figure 3G). Based on the expression level of *GFAP* and combined DAA genes, we analyzed the $GFAP^{low}$, $GFAP^{high}$, and DAA module score along this trajectory (Figure 3H; see methods). At the end of trajectory, the fAD cerebral organoids preferred to differentiate to the fates with high score of the $GFAP^{high}$ and DAA modules (Figure 3H). Thus, the developmental trajectory of glia linage cells in the fAD cerebral organoids resembled disease-related alternations in the astrocytes of AD patients (Morabito et al., 2021). Furthermore, the upregulated DEGs of ASC and GPC in fAD organoids were highly expressed at the end of the trajectory, and the downregulated DEGs were highly expressed at the start and the middle of the trajectory (Figure 3I), indicating again the recurrence of disease-associated states of astrocytes in fAD organoids.

Comparison for gene expression profiles in fAD cerebral organoids and postmortem brain samples

Then we analyzed two single-cell datasets from AD patients (Database: GSE157827 and GSE174367) (Lau et al., 2020; Morabito et al., 2021) (Figures S4A and S4B) and compared with our scRNA-seq data of cerebral organoids. The cell-type-specific DEGs for AD patients were analyzed with the same threshold values used in cerebral organoids (Figures S4C and S4D). Among the DEGs in excitatory neurons, 85 genes showed consistent changes in two sets of patient data, and interestingly, only *TMSB4X* and *PTPRG* genes showed decreased expression in both YN and MN of the four types of fAD cerebral organoids (Figure 4A). *PTPRG* has been identified as a risk gene associated with AD (Herold et al., 2016), and *TMSB4X* has been reported to be a potential anti-inflammatory factor against neurodegenerative disorder (Shomali et al., 2020). In addition, we analyzed DEGs in GPC of all four fAD organoids and ASC of D60 fAD organoids, respectively (Figure 4B). Among them, five genes (*HSPB1*, *MID1*, *ID4*, *FGFR3*, and *BEX3*) showed consistent changes in GPC across four fAD cerebral organoids compared to the corresponding control organoids (Figure 4B). The increased expression of small heat shock protein (*HSPB1*) in astrocytes has been thought to be related to the external stimulation stress of astrocytes, and some studies have shown a certain interaction between *HSPB1* and Aβ (Nafar et al., 2016; Wilhelmus et al., 2006). Meanwhile, other four decreased genes (*MID1*, *ID4*, *FGFR3*, and *BEX3*) also showed the same tendency in ASC of D60 fAD organoids. Notably, several mitochondrial genes, including *MT-ND3* and *MT-ATP6*, were upregulated in YN, MN, and ASC of D60 fAD organoids, consistent with the results of a recent scRNA-seq analysis of a large cohort of AD patients, whose mitochondrial gene expression was significantly upregulated in excitatory neurons, astrocytes, and microglia (Mathys et al., 2023).

We calculated the module scores for the DEGs in YN, MN, or ASC of fAD organoids in specific cell types of control samples and detected the difference of these module scores between control samples and AD patients (Figure 4C; see methods). We found that the module scores of DEGs in YN and MN showed significant difference in excitatory neurons (EX) between control samples and AD patients, whereas only that of "Down in ASC" showed consistent differences in the astrocytes of two patient datasets (Figure 4C). Furthermore, the module scores for "Down in YN" and "Down in MN" showed high score in excitatory neurons but the module scores for "UP in YN" showed high score in endothelial cells (END) (Figure 4C). This result implies that the genes downregulated in the neurons of fAD organoids are highly expressed in the neurons of healthy brains, whereas the genes upregulated in fAD organoids are expressed in non-ectodermal cells of the brain. We also analyzed the module scores of state marker genes of

Figure 3. AD-like features in glial linage cells in fAD cerebral organoids
(A) The differential expression of the DAA gene signatures in astrocytes of fAD cerebral organoids.
(B) The DAA-enriched GO terms in astrocytes of each fAD cerebral organoid.
(C) Heatmap for the expression of differential expressed genes, enriched in Alzheimer's disease pathway, in astrocytes of D30 and D60 organoids. The red color means that the gene exhibits a consistent upregulation in its differential expression within APP2 and HVRD organoids. The blue color means that the gene exhibits a consistent downregulation within APP2 and HVRD organoids.
(D) The top enriched KEGG pathways in astrocytes of each fAD cerebral organoid.
(E and F) The differentiation pathways labeled by pseudotime (E) or cell states (F) in glia progenitor cells and astrocytes.
(G) The proportions of cell states identified from glia progenitor cells and astrocytes in fAD and control cerebral organoids.
(H) Module scores for DAA (*GFAP*, *CSTB*, *VIM*, *OSMR*, *GSN*), $GFAP^{high}$ (*GFAP*, *ID3*, *AQP4*, *MYOC*, *ID1*, *FABP7*) and $GFAP^{low}$ (*LUZP2*, *SLC7A10*, *MFGE8*) gene signatures averaged for nuclei in each of the 25 trajectory bins. Solid color lines represent LOESS regressions for each signature, and the gray outlines represent 95% CIs.
(I) Heatmap for differential expressed genes identified in astrocytes and glia progenitor cells.

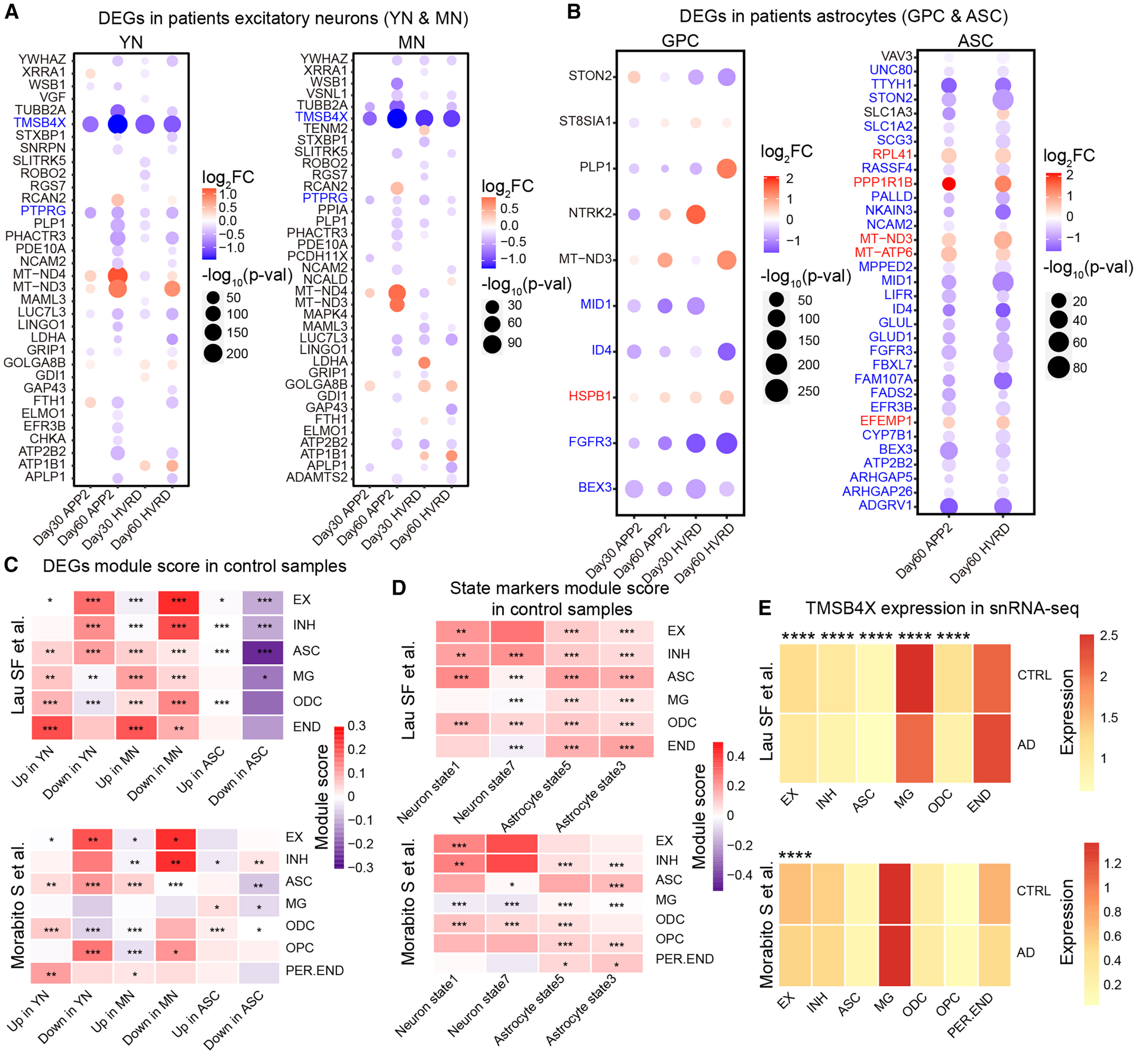

Figure 4. Conjoint analysis of fAD cerebral organoids and patients' data

(A) Differential expressed genes identified in excitatory neurons of AD patients, in young neuron (left) and mature neuron (right) of fAD cerebral organoids.

(B) Differential expressed genes identified in astrocytes of AD patients, in glia progenitor cell (left) and astrocyte (right) of fAD cerebral organoids.

(C and D) Heatmap showing the module scores of differential expressed genes identified from young neurons, mature neurons, and astrocyte (C) or markers identified in pseudotime trajectory analysis (D) of fAD cerebral organoids in major cell types of control brain samples. EX, excitatory neuron; INH, inhibitory neuron; ASC, astrocyte; MG, microglia; ODC, oligodendrocyte; OPC, oligodendrocyte progenitor; END, endothelial; PER.END, pericytes endothelial. Differences of module scores between control and AD patients were tested by Wilcoxon rank test, and p values were overlaid on the heatmap (*$p < 0.05$, **$p < 0.005$, ***$p < 0.0005$).

(E) Heatmap showing the expression of *TMSB4X* in cell types of control and AD patients. ****p.adj <0.0001. See also Figure S4.

neurons and astrocytes detected in trajectory analysis of cell fates in fAD organoids (Figure 4D). We found that the module scores for marker genes of AD-prone state 1 neurons showed significant difference in EX, inhibitory neurons (INH), and oligodendrocytes (ODC) in AD patients versus controls (Figure 4D). And marker genes of

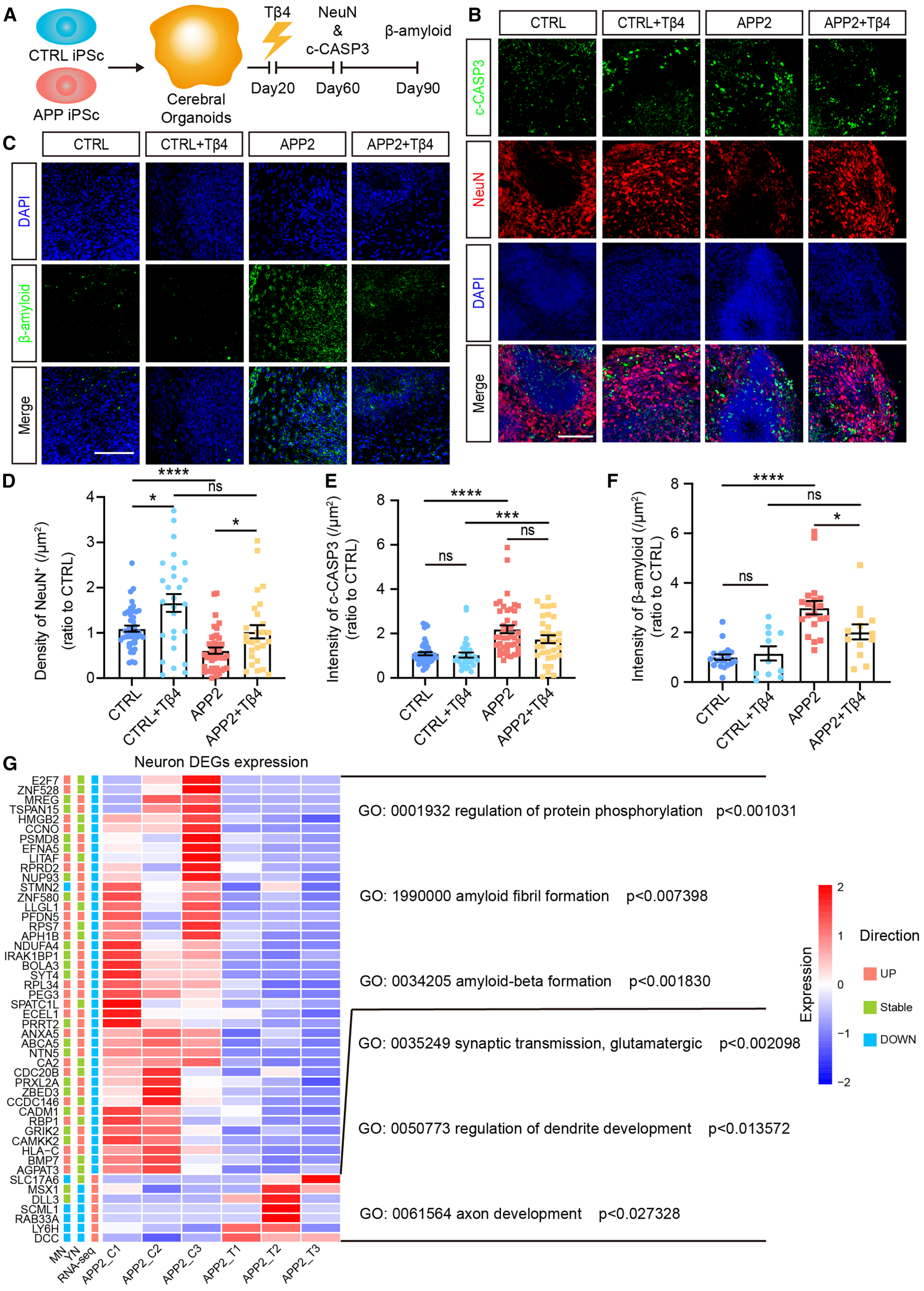

(legend on next page)

normal-prone state 3 in organoid ASC also showed marked differences in the astrocytes from AD patients (Figure 4D). Thus, alterations in cell state trajectory observed in fAD organoids recapitulate to some extent the real cellular changes in AD patients.

The two snRNA-seq datasets of AD patients showed that *TMSB4X* was consistently downregulated in excitatory neurons (Figure 4E). Next, we determined the *TMSB4X* expression levels in organoids. Both fAD organoids (APP2 and HVRD2) exhibited marked downregulation of *TMSB4X* compared to controls (CTRL and CTRL2) (Figure S4E). Thymosin β4 (Tβ4), the product of *TMSB4X*, has been used to treat diverse diseases, such as pressure ulcers (Francis Godschalk, 2007) and dry eye syndrome (Sosne et al., 2012). A recent study has shown that Tβ4 reverses cognitive impairment in mouse model of AD via regulation of microglia polarization and inflammatory response (Wang et al., 2021). Analysis of STRING database (string-db.org) has revealed a set of *TMSB4X* interacting genes (Figure S4F). The top KEGG pathways and GO terms were associated with apoptosis, AD, cytoskeleton, and neurotrophin signaling (Figures S4G and S4H).

To validate the association between *TMSB4X* and AD, control organoids were treated with 5 μM Aβ at day 20 of differentiation, with sampling at day 60 to assess *TMSB4X* expression (Figure S4I). Quantitative analysis revealed a significant downregulation of *TMSB4X* in Aβ-treated organoids (Figure S4J), recapitulating the expression pattern observed in disease contexts. These findings highlight a functional link between *TMSB4X* and AD pathology.

Tβ4 rescues the neurodevelopmental defects and reduces Aβ in fAD cerebral organoids

To determine the effects of Tβ4, control and APP2 cerebral organoids were treated with Tβ4 (0.5 μg/mL) at D20 followed by examination at D60 and D90 (Figure 5A). We found that Tβ4 treatment markedly increased the density of neurons in both control and APP2 D60 organoids (Figures 5B and 5D). APP2 organoids exhibited increased intensity of c-CASP3 signals and TUNEL signals, suggesting the elevation of cell death (Figures 5B, 5E, S5A, and S5B). Notably, Tβ4 treatment had no effect on c-CASP3 signal in APP2 or control organoids (Figure 5E), but significantly inhibited TUNEL intensity in APP2 D60 cerebral organoids (Figure S5B). Furthermore, Tβ4 significantly decreased the level of Aβ in APP2 D90 cerebral organoids (Figures 5C and 5F) and increased the ratio of Aβ1-42/Aβ1-40 in APP2 D60 cerebral organoids (Figures S5C–S5E). These results suggest that Tβ4 treatment attenuates AD-specific features in fAD cerebral organoids.

To investigate the mechanisms of Tβ4 function in fAD cerebral organoids, we performed bulk RNA-seq analysis for D30 APP2 cerebral organoids treated with and without Tβ4, respectively. We found that Tβ4-treated fAD organoids exhibited 911 upregulated genes and 1,395 downregulated genes (Figure S6A). The upregulated genes were enriched in the GO terms related to neurodevelopmental pathways such as “central nervous system neuron axonogenesis” and “detection of calcium ion” (Figure S6B). And the downregulated genes were enriched in some immune response and protein phosphorylation terms like “somatic recombination of immunoglobulin genes involved in immune response” and “negative regulation of phosphorylation” (Figure S6C).

To corroborate the role of Tβ4 in regulating AD-associated signaling, we performed a joint analysis of bulk RNA-seq data of Tβ4-treated APP2 organoids and scRNA-seq data of APP2 organoids (Figures 5G and S6D). Among the DEGs of fAD organoid neurons (YN and MN), 41 genes were downregulated and 7 genes were upregulated in Tβ4-treated organoids (Figure 5G). Several downregulated genes, such as *APH1B* and *NDUFA4*, were involved in “Alzheimer’s disease” pathway. *APH1B* gene encodes Aph-1b, one of the four subunits (Aph-1, nicastrin, presenilin, and Pen-2) of γ-secretase (Gertsik et al., 2014). The decrease of *APH1B* might inhibit γ-secretase-mediated processing of APP. The downregulated genes were related to amyloid-beta formation, while Tβ4 upregulated genes focused on neuron and synapse development (Figure 5G). We also analyzed the effects of Tβ4 on gene expression of GPC

Figure 5. Thymosin β4 protects neurons and attenuates Aβ production in fAD cerebral organoids

(A) Schematic representation of thymosin β4 treatment of fAD cerebral organoids and timeline of the analysis.

(B) Immunofluorescence for mature neuron (NeuN) and apoptosis signal (c-CASP3) in D60 cerebral organoids. Scale bars, 100 μm.

(C) Immunofluorescence for Aβ in D90 cerebral organoids. Scale bars, 100 μm.

(D and E) Quantification of the density of NeuN-positive cells (D) and the intensity of c-CASP3 signal (E) in D60 cerebral organoids. Data are presented as mean ± SEM of at least nine organoids (three fields per organoid) per group from at least three independent experiments, with the value of control group normalized as 1.0. Mann-Whitney test. $^*p < 0.05$, $^{***}p < 0.001$, $^{****}p < 0.0001$.

(F) Quantification of the intensity of β-amyloid in D90 cerebral organoids. Data are presented as mean ± SEM of at least 11 organoids per group from at least three independent experiments, with the value of control group normalized as 1.0. Mann-Whitney test. $^*p < 0.05$, $^{****}p < 0.0001$.

(G) Heatmap showing the differential expressed genes identified in young neurons and mature neurons of Tβ4-treated (T1, T2, and T3) D30 APP2 cerebral organoids with specific GO terms listed. Vehicle treatments were used as controls (C1, C2, and C3). See also Figures S5 and S6.

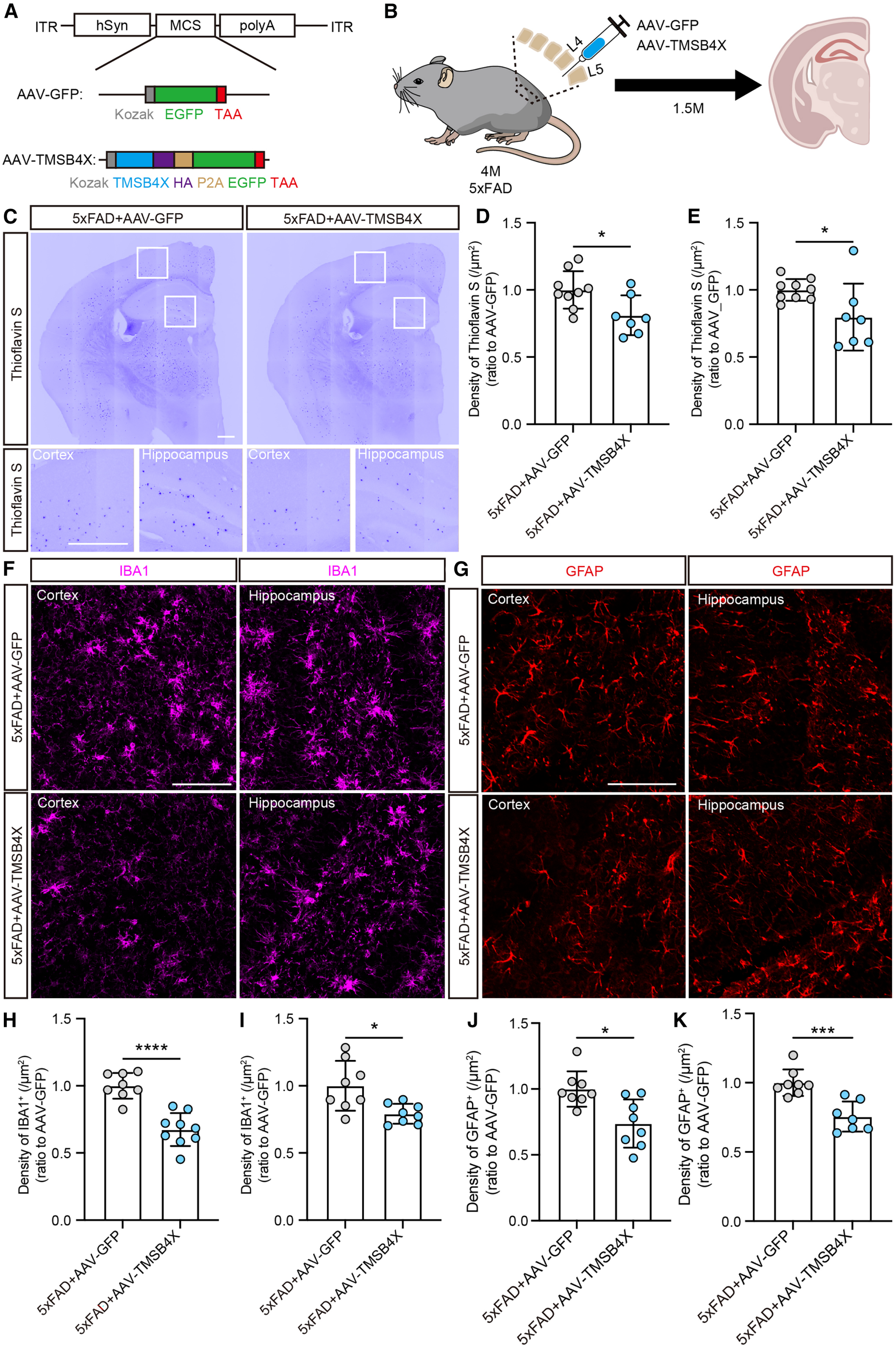

(legend on next page)

and ASC DEGs (Figure S6D). The treatment with Tβ4 was found to reverse the upregulation of *TMEM132C* and *PEG3*, and the downregulation of *DHCR7* observed in the ASC and GPC of D30 APP2 organoids.

Administration of AAV-TMSB4X alleviates pathological changes and neuronal excitability in 5xFAD mice

Finally, we determined the expression and roles of *TMSB4X* in animal models *in vivo*. Notably, *Tmsb4x* expression was upregulated in the hippocampus of 5xFAD mice compared to wild-type (WT) controls (Figure S7A), consistent with prior RNA-seq data from 6- and 11-month-old 5xFAD mice (Database: GSE142633) (Figures S7B and S7C). The difference with human samples may reflect species- or cell-type-specific transcriptional adaptations to neurodegenerative stress. We constructed a plasmid with the human synapsin (hSyn) promoter to achieve overexpression of *TMSB4X* in neurons (Figure 6A) and used the AAV-PhP. eB capsid to encapsulate the plasmid and infect brain neurons of 5xFAD mice by intrathecal injection (Figure 6B). *TMSB4X* was overexpressed in the cortex and hippocampus of 5xFAD mice after virus infection for 1.5 months (Figures S7D–S7F). Consistent with the phenotypes in Tβ4-treated fAD organoids and *TMSB4X*-overexpressed APP/PS1 mice (Wang et al., 2021), overexpression of *TMSB4X* effectively inhibited the accumulation of amyloid plaques in 5xFAD mice (Figures 6C–6E, S7G, and S7H). For the soluble Aβ, injection of AAV-TMSB4X resulted in a reduction of Aβ1-40 levels and an increase in the Aβ1-42/Aβ1-40 ratio (Figures S7I–S7K). Meanwhile, the proliferation of microglia and astrocyte in AD mice was also markedly inhibited by AAV-TMSB4X injection (Figures 6F–6K). In addition to attenuating gliosis in 5xFAD brain, injection of AAV-TMSB4X also suppressed the expression of *Tnf-α* and *Il6* in the hippocampus (Figures S7L and S7M). These results indicate that overexpressing *TMSB4X* in neurons of AD mice can effectively alleviate AD pathology.

In addition to common AD pathological changes, neurons in AD usually exhibit hyperexcitability (Siskova et al., 2014). In our previous results of DEGs analysis of Tβ4-treated organoids, Tβ4 was observed to affect genes related to synapse formation and neural signal transmission (Figures 5G and S6B). Thus, we determined whether overexpression of *TMSB4X* had any effects on the hyperexcitability of neurons in AD. By analyzing the action potentials (AP) of cortical excitatory neurons in 6- to 7-month-old WT and 5xFAD mice (Figure 7A), we found that excitatory neurons in 5xFAD mice showed obvious hyperexcitability characteristics including an increase in half-width, a decrease in threshold potential, and an increase in amplitude (Figures 7C–7E). Interestingly, this hyperexcitability was markedly alleviated in AD mice with *TMSB4X* overexpression (Figures 7B–7E).

To investigate the mechanism of AAV-TMSB4X in 5xFAD mice, we performed RNA-seq for 5xFAD mice injected with AAV-GFP and AAV-TMSB4X, respectively (Figure 7F). The exogenous TMSB4X-HA was detected exclusively in the AAV-TMSB4X group (Figure S7N). Differential gene expression analysis between AAV-GFP- and AAV-TMSB4X-injected 5xFAD mice identified 627 upregulated and 827 downregulated genes (Figure S7O). Principal-component analysis (PCA) further revealed distinct transcriptional profiles between AAV-GFP and AAV-TMSB4X groups (Figure S7P). GO enrichment analysis of upregulated gens revealed significant clustering in neural signaling pathways such as "membrane depolarization" and "regulation of ion transmembrane transport" (Figure 7G), potentially uncovering the mechanism by which AAV-TMSB4X reduces neuronal hyperexcitability. Conversely, downregulated genes were primarily enriched in small GTPase and Ras protein signal transduction pathways (Figure 7H). It has been shown that H-Ras deletion improves memory, reduces amyloid plaques, and protects dendrites in AD mice (Qu et al., 2023). These suggest that AAV-TMSB4X may reduce amyloid plaques production in 5xFAD mice by inhibiting the

Figure 6. Administration of AAV-TMSB4X alleviates amyloid plaques and gliosis
(A) Construct design of AAV-GFP and AAV-TMSB4X vectors, with promoter and expression elements inserted between ITRs.
(B) Schematic diagram of the AAV injection in 5xFAD mice.
(C) Representative images for amyloid plaques (Thioflavin S) in AAV-GFP- and AAV-TMSB4X-injected 5xFAD mice. Scale bar, 100 μm.
(D and E) Quantification of the density of amyloid plaques (Thioflavin S) in the cortex (D) or hippocampus (E) of AAV-GFP- ($n = 9$ mice) and AAV-TMSB4X ($n = 7$ mice)-injected 5xFAD mice. Data are presented as mean ± SEM with the value of AAV-GFP-injected mice normalized as 1.0. Mann-Whitney test. $^*p < 0.05$.
(F) Immunofluorescence for IBA1-labeled microglial cells in AAV-GFP- and AAV-TMSB4X-injected 5xFAD mice. Scale bars, 100 μm.
(G) Immunofluorescence for GFAP-labeled activated astrocytes in AAV-GFP- and AAV-TMSB4X-injected 5xFAD mice. Scale bars, 100 μm.
(H and I) Quantification of the density of IBA1-positive cells in the cortex (H) or hippocampus (I) of AAV-GFP- and AAV-TMSB4X-injected 5xFAD mice. Data are presented as mean ± SEM of at least eight mice in each group with the value of AAV-GFP group normalized as 1.0. Mann-Whitney test. $^*p < 0.05$, $^{****}p < 0.0001$.
(J and K) Quantification of the density of GFAP-positive cells in the cortex (J) or hippocampus (K) of AAV-GFP- and AAV-TMSB4X-injected 5xFAD mice. Data are presented as mean ± SEM of at least seven mice in each group with the value of AAV-GFP group normalized as 1.0. Mann-Whitney test. $^*p < 0.05$, $^{***}p < 0.001$. See also Figure S7.

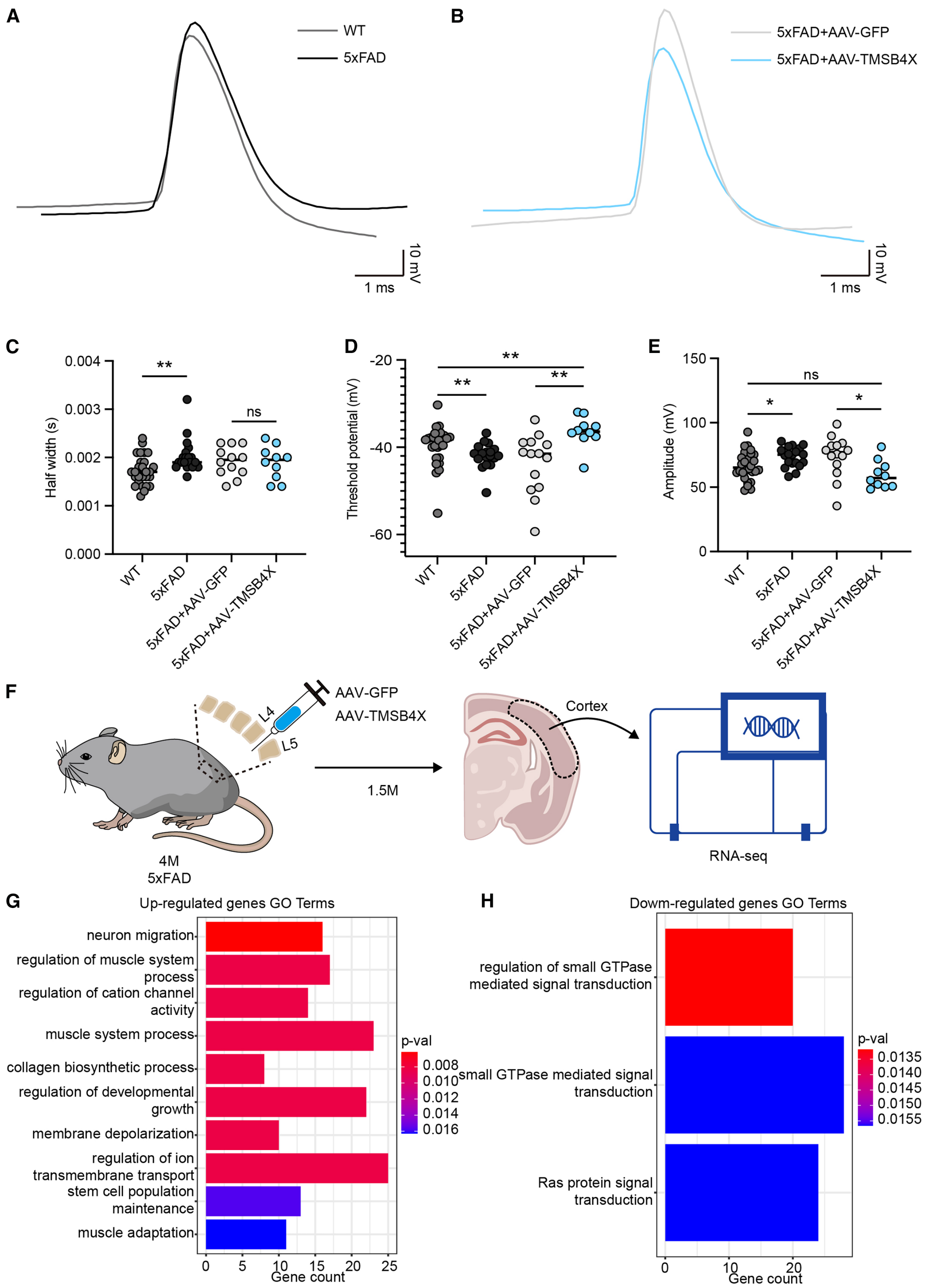

(legend on next page)

expression of Ras proteins. Taken together, *TMSB4X* has exhibited effects on AD pathology both in AD brain organoids and model mice.

DISCUSSION

In this study, we have used cerebral organoids to understand the cellular and molecular alterations during early neurogenesis of AD subjects. We found increased levels of Aβ in both fAD cerebral organoids at a late stage, attenuated genesis of mature neurons, and increased cell apoptosis at early stages. Furthermore, fAD organoids showed changes in the transcriptomes and developmental trajectories in a cell-type-specific manner. Additionally, we have identified *TMSB4X* as a target gene, whose downregulation contributes to impaired neurogenesis and the production of Aβ in fAD organoids. Intriguingly, overexpressing *TMSB4X* in the neurons of 5xFAD mice mitigated AD pathology and neuronal hyperexcitability.

Previous studies involving AD organoids utilizing serum samples from AD patients and iPSCs from patients with familial PSEN mutations and sporadic APOE mutations have all observed increased levels of Aβ (Chen et al., 2021; Vanova et al., 2023; Zhao et al., 2020). However, unlike these studies, we employed scRNA-seq to understand any changes in the neurogenesis and neuronal maturation of fAD organoids. After annotating the cell types of cells, mature neurons were observed to be reduced in fAD cerebral organoids. In line with this result, a previous study has shown a reduction of neurons in the cerebral organoids treated with serum from AD patients (Chen et al., 2021). The increased apoptotic signaling in AD cerebral organoids has also been observed in APOE-mutated organoids (Zhao et al., 2020).

Furthermore, we combined our single-cell RNA data with single-nuclear RNA data of AD patients (Lau et al., 2020; Morabito et al., 2021) and focused on *TMSB4X*, which showed consistent downregulation in both neurons of fAD cerebral organoids and excitatory neurons of AD patients. We found Tβ4 treatment mitigated reduced mature neurons and increased Aβ in fAD cerebral organoids. It has been shown that overexpression of Tβ4 reverses cognitive impairment in mouse model of AD via regulation of microglia polarization and inflammatory response (Wang et al., 2021). However, our result suggests that Tβ4 treatment can reduce Aβ production independent of microglia cells. This discrepancy might reflect multifaceted roles of Tβ4. Previous studies have shown that AD neurons exhibit hyperexcitability (Siskova et al., 2014), which is accompanied by abnormalities in calcium ion channels (Targa Dias Anastacio et al., 2022) and closely related to neuronal death (Sukumaran et al., 2021). Our sequencing analysis of fAD organoids treated with Tβ4 indicates that Tβ4 regulates the expression of genes related to synapse and nerve signal transmission. And overexpression of *TMSB4X* in neurons of the cortex in 5xFAD mice effectively alleviated neuronal hyperexcitability. The beneficial effects of Tβ4 in fAD organoids and animal models point to the potential as an intervention target against AD pathology.

Despite challenges in modeling irreversible neurodegenerative impacts with cerebral organoids, our comparative analysis revealed cell-type-specific early developmental alterations, which is unattainable with postmortem brain samples. Like other brain organoid studies, this study has limitations, in particular batch-to-batch and intra-batch heterogeneity. To address this issue, we applied within-batch corrections to control groups. Another limitation is the difference in cell types between organoids and human brains. To address this issue, we focused on cell types shared between organoids and the developing human brain. The third limitation is the absence of non-ectodermal cells in current cerebral organoid models, such as microglia and vascular cells, which are believed to influence disease progression (Nortley et al., 2019; Sun et al., 2023). Recently, we have established vascularized brain organoids that contain extensive amount of microglia and brain-blood barrier structures (Sun et al., 2022). This system will enable us to investigate the changes and roles of these cell types and intercellular interactions during AD progression.

METHODS

Animals

All animal experiments, including mouse rearing, breeding, and surgical operations, were executed in compliance with the ethical guidelines of the Institutional Animal Care and

Figure 7. Administration of AAV-TMSB4X alleviates neuronal hyperexcitability in 5xFAD mice

(A) Sample traces of action potential from excitatory neurons in the cortex of WT and 5xFAD mice.

(B) Sample traces of action potential from excitatory neurons in the cortex of 5xFAD mice injected with AAV-GFP and AAV-TMSB4X, respectively.

(C–E) Quantification of half-width (C), threshold potential (D), and the amplitude (E) of the action potential from excitatory neurons in indicated mice. Data are presented as mean ± SEM of at least 10 neurons from at least three mice per group. Mann-Whitney test. $^*p < 0.05$, $^{**}p < 0.01$.

(F) Schematic diagram of the bulk RNA-seq for AAV-injected 5xFAD mice.

(G and H) GO terms enriched by the upregulated genes (G) and downregulated genes (H) in AAV-injected 5xFAD mice. See also Figure S7.

Use Committee of ShanghaiTech University. For additional information on mouse husbandry and mouse strains, see supplemental methods.

Cerebral organoids culture, treatments with Tβ4 and Aβ

Cerebral organoids were cultured following instructions of the commercial STEMdiffTM Cerebral Organoid Kit (STEMCELL #08570). For additional information on organoid generation, see supplemental methods. For Tβ4-treated cerebral organoids, the medium was replaced by Maturation Medium with the addition of Tβ4 (0.5 μg/mL, Abcam, #ab245823) at day 20 and replaced every 5 days. For Aβ-treated cerebral organoids, the medium was replaced by maturation medium with the addition of amyloid β peptide (1–42) human (5 μM, Beyotime, #P9001-1mg) at day 20 and replaced every 5 days.

Single-cell RNA-seq

Organoids were dissociated using the methods described in a previous study (Thomsen et al., 2016). For additional information on single-cell RNA-seq and analysis, see supplemental methods.

Bulk RNA-seq, quality control, and DEG analysis

Tβ4-treated fAD cerebral organoids and cortex from 5xFAD mice with AAV-GFP and AAV-TMSB4X injected, respectively, were sequenced on illumine Novaseq 6000 pipeline. Raw reads were mapped to the human reference genome GRCh38 (v107) by Hisat2 using default parameter. Gene expression level was calculated as count number by htseq-count software. DEG analyses were performed by edgeR (v3.38.4 R package) with p value <0.05 and log2 fold change >1 (Robinson et al., 2010).

Intrathecal injection

The specific procedure for intrathecal injection follows established protocols as described previously (Cheng et al., 2023). The fur from the tail to the thoracic vertebra was shaved using a razor blade on mice anesthetized with avertin (500 mg/kg, i.p.). The AAV virus (5×10^{11} vg per mouse, 50 μL) was then injected into the intrathecal space between either the L4 and L5 or L3 and L4 vertebrae using a 30 G needle. The needle was removed 30 s post-injection, ensuring no fluid leakage occurred. After the injection, the mice were placed in a recovery cage and observed until normal activities resumed.

Statistical analysis

Statistical analyses for immunostaining signals and action potentials were performed using the GraphPad Prism software v.9.5.1. Significant differences between the two groups were calculated using a two-tailed Mann-Whitney test or Student's t test. Statistical analyses for sequencing data were performed using R software. Statistical significance was set at $p < 0.05$. Appropriate methods are indicated within the legends, and significant differences are marked in all figures. Values and error bars are presented as mean ± SEM as noted in the figure legends.

RESOURCE AVAILABILITY

Lead contact

Requests for further information may be directed to the lead contact, Zhen-Ge Luo (luozhg@shanghaitech.edu.cn).

Materials availability

Reagents reported in this paper is available from the lead contact upon request.

Data and code availability

The accession numbers for single-cell RNA sequencing transcriptome data and bulk RNA-seq data supporting this study are: SRP510960 (scRNA-seq), SRP514285 (bulk RNA-seq for organoids), and SRP593290 (bulk RNA-seq for 5xFAD mice), which can be accessed at https://www.ncbi.nlm.nih.gov/sra. This paper does not report original code.

ACKNOWLEDGMENTS

This work was partially supported by National Key Research and Development Program of China (2024YFA1108000, 2021ZD0202500), National Natural Science Foundation of China (32130035 and 92168107), The Joint Project of the Yangtze River Delta Science and Technology Innovation Community (2024CSJZN0600), Central Guidance on Local Science and Technology Development Fund (YDZX20233100001002), Shanghai Clinical Research and Trail Center, and Shanghai Frontiers Science Center for Biomacromolecules and Precision Medicine at ShanghaiTech University. We thank the MultiOmics Core Facility, Molecular Imaging Core Facility, Molecular and Cell Biology Core Facility, and model animal facility at the School of Life Science and Technology, ShanghaiTech University, for providing technical support.

AUTHOR CONTRIBUTIONS

P.-M.Z., X.-Y.S., X.-C.J., and Z.-G.L. designed the research. P.-M.Z. performed most of the experiments and prepared the figures. X.-Y.S., Y.L., and W.-D.W. provided assistance for organoids experiments. J.H. and P.-J.Q. provided assistance for mouse experiments. D.D.C. performed electrophysiology recoding. P.-M.Z. and Z.-G.L. wrote the manuscript. Z.-G.L. supervised the whole project.

DECLARATION OF INTERESTS

A related patent pertaining to the application of TMSB4X on AD treatment has been filed (patent applicant: ShanghaiTech University, inventors: Z.-G.L. and P.-M.Z., status: pending).

SUPPLEMENTAL INFORMATION

Supplemental information can be found online at https://doi.org/10.1016/j.stemcr.2025.102601.

Received: February 7, 2025
Revised: July 17, 2025
Accepted: July 18, 2025
Published: August 14, 2025

REFERENCES

Busche, M.A., and Konnerth, A. (2016). Impairments of neural circuit function in Alzheimer's disease. Philos. Trans. R. Soc. Lond. B Biol. Sci. *371*, 20150429. https://doi.org/10.1098/rstb.2015.0429.

Chen, X., Sun, G., Tian, E., Zhang, M., Davtyan, H., Beach, T.G., Reiman, E.M., Blurton-Jones, M., Holtzman, D.M., and Shi, Y. (2021). Modeling Sporadic Alzheimer's Disease in Human Brain Organoids under Serum Exposure. Adv. Sci. *8*, e2101462. https://doi.org/10.1002/advs.202101462.

Cheng, W., Huang, J., Fu, X.Q., Tian, W.Y., Zeng, P.M., Li, Y., and Luo, Z.G. (2023). Intrathecal delivery of AAV-NDNF ameliorates disease progression of ALS mice. Mol. Ther. *31*, 3277–3289. https://doi.org/10.1016/j.ymthe.2023.09.018.

Crews, L., Adame, A., Patrick, C., Delaney, A., Pham, E., Rockenstein, E., Hansen, L., and Masliah, E. (2010). Increased BMP6 levels in the brains of Alzheimer's disease patients and APP transgenic mice are accompanied by impaired neurogenesis. J. Neurosci. *30*, 12252–12262. https://doi.org/10.1523/JNEUROSCI.1305-10.2010.

Delaby, C., Estelles, T., Zhu, N., Arranz, J., Barroeta, I., Carmona-Iragui, M., Illan-Gala, I., Santos-Santos, M.A., Altuna, M., Sala, I., et al. (2022). The Abeta1-42/Abeta1-40 ratio in CSF is more strongly associated to tau markers and clinical progression than Abeta1-42 alone. Alzheimers Res. Ther. *14*, 20. https://doi.org/10.1186/s13195-022-00967-z.

Francis Godschalk, M. (2007). Pressure ulcers: a role for thymosin beta4. Ann. N. Y. Acad. Sci. *1112*, 413–417. https://doi.org/10.1196/annals.1415.049.

Gertsik, N., Chiu, D., and Li, Y.M. (2014). Complex regulation of gamma-secretase: from obligatory to modulatory subunits. Front. Aging Neurosci. *6*, 342. https://doi.org/10.3389/fnagi.2014.00342.

Ghatak, S., Dolatabadi, N., Gao, R., Wu, Y., Scott, H., Trudler, D., Sultan, A., Ambasudhan, R., Nakamura, T., Masliah, E., et al. (2021). NitroSynapsin ameliorates hypersynchronous neural network activity in Alzheimer hiPSC models. Mol. Psychiatry *26*, 5751–5765. https://doi.org/10.1038/s41380-020-0776-7.

Giandomenico, S.L., and Lancaster, M.A. (2017). Probing human brain evolution and development in organoids. Curr. Opin. Cell Biol. *44*, 36–43. https://doi.org/10.1016/j.ceb.2017.01.001.

Habib, N., McCabe, C., Medina, S., Varshavsky, M., Kitsberg, D., Dvir-Szternfeld, R., Green, G., Dionne, D., Nguyen, L., Marshall, J.L., et al. (2020). Disease-associated astrocytes in Alzheimer's disease and aging. Nat. Neurosci. *23*, 701–706. https://doi.org/10.1038/s41593-020-0624-8.

Hamilton, L.K., Aumont, A., Julien, C., Vadnais, A., Calon, F., and Fernandes, K.J.L. (2010). Widespread deficits in adult neurogenesis precede plaque and tangle formation in the 3xTg mouse model of Alzheimer's disease. Eur. J. Neurosci. *32*, 905–920. https://doi.org/10.1111/j.1460-9568.2010.07379.x.

Hardy, J., and Selkoe, D.J. (2002). The amyloid hypothesis of Alzheimer's disease: progress and problems on the road to therapeutics. Science *297*, 353–356. https://doi.org/10.1126/science.1072994.

Haughey, N.J., Nath, A., Chan, S.L., Borchard, A.C., Rao, M.S., and Mattson, M.P. (2002). Disruption of neurogenesis by amyloid beta-peptide, and perturbed neural progenitor cell homeostasis, in models of Alzheimer's disease. J. Neurochem. *83*, 1509–1524. https://doi.org/10.1046/j.1471-4159.2002.01267.x.

Herold, C., Hooli, B.V., Mullin, K., Liu, T., Roehr, J.T., Mattheisen, M., Parrado, A.R., Bertram, L., Lange, C., and Tanzi, R.E. (2016). Family-based association analyses of imputed genotypes reveal genome-wide significant association of Alzheimer's disease with OSBPL6, PTPRG, and PDCL3. Mol. Psychiatry *21*, 1608–1612. https://doi.org/10.1038/mp.2015.218.

Israel, M.A., Yuan, S.H., Bardy, C., Reyna, S.M., Mu, Y., Herrera, C., Hefferan, M.P., Van Gorp, S., Nazor, K.L., Boscolo, F.S., et al. (2012). Probing sporadic and familial Alzheimer's disease using induced pluripotent stem cells. Nature *482*, 216–220. https://doi.org/10.1038/nature10821.

Lancaster, M.A., and Knoblich, J.A. (2014). Generation of cerebral organoids from human pluripotent stem cells. Nat. Protoc. *9*, 2329–2340. https://doi.org/10.1038/nprot.2014.158.

Lau, S.F., Cao, H., Fu, A.K.Y., and Ip, N.Y. (2020). Single-nucleus transcriptome analysis reveals dysregulation of angiogenic endothelial cells and neuroprotective glia in Alzheimer's disease. Proc. Natl. Acad. Sci. USA *117*, 25800–25809. https://doi.org/10.1073/pnas.2008762117.

Mathys, H., Peng, Z., Boix, C.A., Victor, M.B., Leary, N., Babu, S., Abdelhady, G., Jiang, X., Ng, A.P., Ghafari, K., et al. (2023). Single-cell atlas reveals correlates of high cognitive function, dementia, and resilience to Alzheimer's disease pathology. Cell *186*, 4365–4385.e27. https://doi.org/10.1016/j.cell.2023.08.039.

Morabito, S., Miyoshi, E., Michael, N., Shahin, S., Martini, A.C., Head, E., Silva, J., Leavy, K., Perez-Rosendahl, M., and Swarup, V. (2021). Single-nucleus chromatin accessibility and transcriptomic characterization of Alzheimer's disease. Nat. Genet. *53*, 1143–1155. https://doi.org/10.1038/s41588-021-00894-z.

Muratore, C.R., Rice, H.C., Srikanth, P., Callahan, D.G., Shin, T., Benjamin, L.N.P., Walsh, D.M., Selkoe, D.J., and Young-Pearse, T.L. (2014). The familial Alzheimer's disease APPV717I mutation alters APP processing and Tau expression in iPSC-derived neurons. Hum. Mol. Genet. *23*, 3523–3536. https://doi.org/10.1093/hmg/ddu064.

Murayama, K.S., Kametani, F., Saito, S., Kume, H., Akiyama, H., and Araki, W. (2006). Reticulons RTN3 and RTN4-B/C interact with BACE1 and inhibit its ability to produce amyloid beta-protein. Eur. J. Neurosci. *24*, 1237–1244. https://doi.org/10.1111/j.1460-9568.2006.05005.x.

Nafar, F., Williams, J.B., and Mearow, K.M. (2016). Astrocytes release HspB1 in response to amyloid-beta exposure in vitro. J. Alzheimers Dis. *49*, 251–263. https://doi.org/10.3233/JAD-150317.

Nortley, R., Korte, N., Izquierdo, P., Hirunpattarasilp, C., Mishra, A., Jaunmuktane, Z., Kyrargyri, V., Pfeiffer, T., Khennouf, L., Madry, C., et al. (2019). Amyloid beta oligomers constrict human capillaries in Alzheimer's disease via signaling to pericytes. Science *365*, eaav9518. https://doi.org/10.1126/science.aav9518.

Pang, Q.M., Zhang, Q., Wu, X.C., Yang, R.L., Fu, S.P., Fan, Z.H., Liu, J., Yu, L.M., Peng, J.C., and Zhang, T. (2023). Mechanism of M2 macrophages modulating astrocyte polarization through the TGF-beta/PI3K/Akt pathway. Immunol. Lett. *259*, 1–8. https://doi.org/10.1016/j.imlet.2023.05.009.

Qu, W., Jeong, A., Zhong, R., Thieschafer, J.S., Gram, A., and Li, L. (2023). Deletion of Small GTPase H-Ras Rescues Memory Deficits and Reduces Amyloid Plaque-Associated Dendritic Spine Loss in Transgenic Alzheimer's Mice. Mol. Neurobiol. *60*, 495–511. https://doi.org/10.1007/s12035-022-03082-0.

Robinson, M.D., McCarthy, D.J., and Smyth, G.K. (2010). edgeR: a Bioconductor package for differential expression analysis of digital gene expression data. Bioinformatics *26*, 139–140. https://doi.org/10.1093/bioinformatics/btp616.

Sato, C., Barthelemy, N.R., Mawuenyega, K.G., Patterson, B.W., Gordon, B.A., Jockel-Balsarotti, J., Sullivan, M., Crisp, M.J., Kasten, T., Kirmess, K.M., et al. (2018). Tau Kinetics in Neurons and the Human Central Nervous System. Neuron *97*, 1284–1298.e7. https://doi.org/10.1016/j.neuron.2018.02.015.

Shomali, N., Baradaran, B., Deljavanghodrati, M., Akbari, M., Hemmatzadeh, M., Mohammadi, H., Jang, Y., Xu, H., and Sandoghchian Shotorbani, S. (2020). A new insight into thymosin beta4, a promising therapeutic approach for neurodegenerative disorders. J. Cell. Physiol. *235*, 3270–3279. https://doi.org/10.1002/jcp.29293.

Siskova, Z., Justus, D., Kaneko, H., Friedrichs, D., Henneberg, N., Beutel, T., Pitsch, J., Schoch, S., Becker, A., von der Kammer, H., and Remy, S. (2014). Dendritic structural degeneration is functionally linked to cellular hyperexcitability in a mouse model of Alzheimer's disease. Neuron *84*, 1023–1033. https://doi.org/10.1016/j.neuron.2014.10.024.

Sosne, G., Qiu, P., Ousler, G.W., 3rd, Dunn, S.P., and Crockford, D. (2012). Thymosin beta4: a potential novel dry eye therapy. Ann. N. Y. Acad. Sci. *1270*, 45–50. https://doi.org/10.1111/j.1749-6632.2012.06682.x.

Sukumaran, P., Nascimento Da Conceicao, V., Sun, Y., Ahamad, N., Saraiva, L.R., Selvaraj, S., and Singh, B.B. (2021). Calcium Signaling Regulates Autophagy and Apoptosis. Cells *10*, 2125. https://doi.org/10.3390/cells10082125.

Sun, N., Victor, M.B., Park, Y.P., Xiong, X., Scannail, A.N., Leary, N., Prosper, S., Viswanathan, S., Luna, X., Boix, C.A., et al. (2023). Human microglial state dynamics in Alzheimer's disease progression. Cell *186*, 4386–4403.e29. https://doi.org/10.1016/j.cell.2023.08.037.

Sun, X.Y., Ju, X.C., Li, Y., Zeng, P.M., Wu, J., Zhou, Y.Y., Shen, L.B., Dong, J., Chen, Y.J., and Luo, Z.G. (2022). Generation of vascularized brain organoids to study neurovascular interactions. eLife *11*, e76707. https://doi.org/10.7554/eLife.76707.

Targa Dias Anastacio, H., Matosin, N., and Ooi, L. (2022). Neuronal hyperexcitability in Alzheimer's disease: what are the drivers behind this aberrant phenotype? Transl. Psychiatr. *12*, 257. https://doi.org/10.1038/s41398-022-02024-7.

Thomsen, E.R., Mich, J.K., Yao, Z., Hodge, R.D., Doyle, A.M., Jang, S., Shehata, S.I., Nelson, A.M., Shapovalova, N.V., Levi, B.P., and Ramanathan, S. (2016). Fixed single-cell transcriptomic characterization of human radial glial diversity. Nat. Methods *13*, 87–93. https://doi.org/10.1038/nmeth.3629.

Vanova, T., Sedmik, J., Raska, J., Amruz Cerna, K., Taus, P., Pospisilova, V., Nezvedova, M., Fedorova, V., Kadakova, S., Klimova, H., et al. (2023). Cerebral organoids derived from patients with Alzheimer's disease with PSEN1/2 mutations have defective tissue patterning and altered development. Cell Rep. *42*, 113310. https://doi.org/10.1016/j.celrep.2023.113310.

Wang, M., Feng, L.R., Li, Z.L., Ma, K.G., Chang, K.W., Chen, X.L., Yang, P.B., Ji, S.F., Ma, Y.B., Han, H., et al. (2021). Thymosin beta4 reverses phenotypic polarization of glial cells and cognitive impairment via negative regulation of NF-kappaB signaling axis in APP/PS1 mice. J. Neuroinflammation *18*, 146. https://doi.org/10.1186/s12974-021-02166-3.

Wang, X., Wang, Z., Chen, Y., Huang, X., Hu, Y., Zhang, R., Ho, M. S., and Xue, L. (2014). FoxO mediates APP-induced AICD-dependent cell death. Cell Death Dis. *5*, e1233. https://doi.org/10.1038/cddis.2014.196.

Wen, P.H., Hof, P.R., Chen, X., Gluck, K., Austin, G., Younkin, S.G., Younkin, L.H., DeGasperi, R., Gama Sosa, M.A., Robakis, N.K., et al. (2004). The presenilin-1 familial Alzheimer disease mutant P117L impairs neurogenesis in the hippocampus of adult mice. Exp. Neurol. *188*, 224–237. https://doi.org/10.1016/j.expneurol.2004.04.002.

Wilhelmus, M.M.M., Boelens, W.C., Otte-Höller, I., Kamps, B., de Waal, R.M.W., and Verbeek, M.M. (2006). Small heat shock proteins inhibit amyloid-beta protein aggregation and cerebrovascular amyloid-beta protein toxicity. Brain Res. *1089*, 67–78. https://doi.org/10.1016/j.brainres.2006.03.058.

Zhao, H., Ji, Q., Wu, Z., Wang, S., Ren, J., Yan, K., Wang, Z., Hu, J., Chu, Q., Hu, H., et al. (2022). Destabilizing heterochromatin by APOE mediates senescence. Nat. Aging *2*, 303–316. https://doi.org/10.1038/s43587-022-00186-z.

Zhao, J., Fu, Y., Yamazaki, Y., Ren, Y., Davis, M.D., Liu, C.C., Lu, W., Wang, X., Chen, K., Cherukuri, Y., et al. (2020). APOE4 exacerbates synapse loss and neurodegeneration in Alzheimer's disease patient iPSC-derived cerebral organoids. Nat. Commun. *11*, 5540. https://doi.org/10.1038/s41467-020-19264-0.

Stem Cell Reports, Volume *20*

Supplemental Information

Thymosin beta 4 as an Alzheimer disease intervention target identified using human brain organoids

Peng-Ming Zeng, Xin-Yao Sun, Yang Li, Wen-di Wu, Jing Huang, Dong-Dong Cao, Pin-jue Qian, Xiang-Chun Ju, and Zhen-Ge Luo

Supplemental Figures and Legends

Figure S1

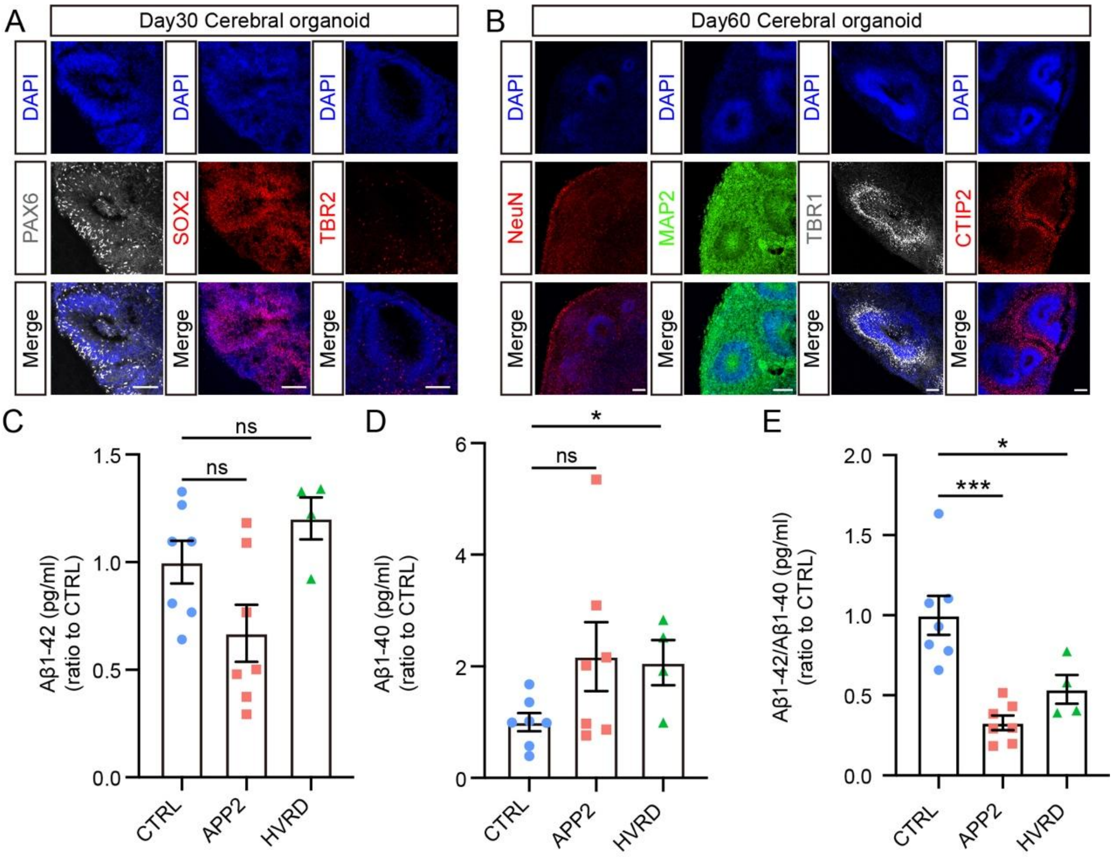

Figure S1. The analysis of iPSC-derived cerebral organoids, related to Figure 1. (A) Representative images of D30 organoids showing the expression of neural progenitor markers, including PAX6, SOX2, and TBR2. Scale bar, 100 μm. (B) Representative images of D60 organoids showing the expression of markers of differentiated neurons including NeuN, MAP2, TBR1, and CTIP2. Scale bar, 100 μm. (C) ELISA results of Aβ1-42 in the medium of D60 cerebral organoids. (D) ELISA results of Aβ1-40 in the medium of D60 cerebral organoids. (E) Ratios of Aβ1-42/Aβ1-40 from ELISA. Data are presented as mean ± SEM of 4-7 organoids per group. The value of the control group was normalized as 1.0. Mann-Whitney test. *P < 0.05, ***P < 0.001.

Figure S2

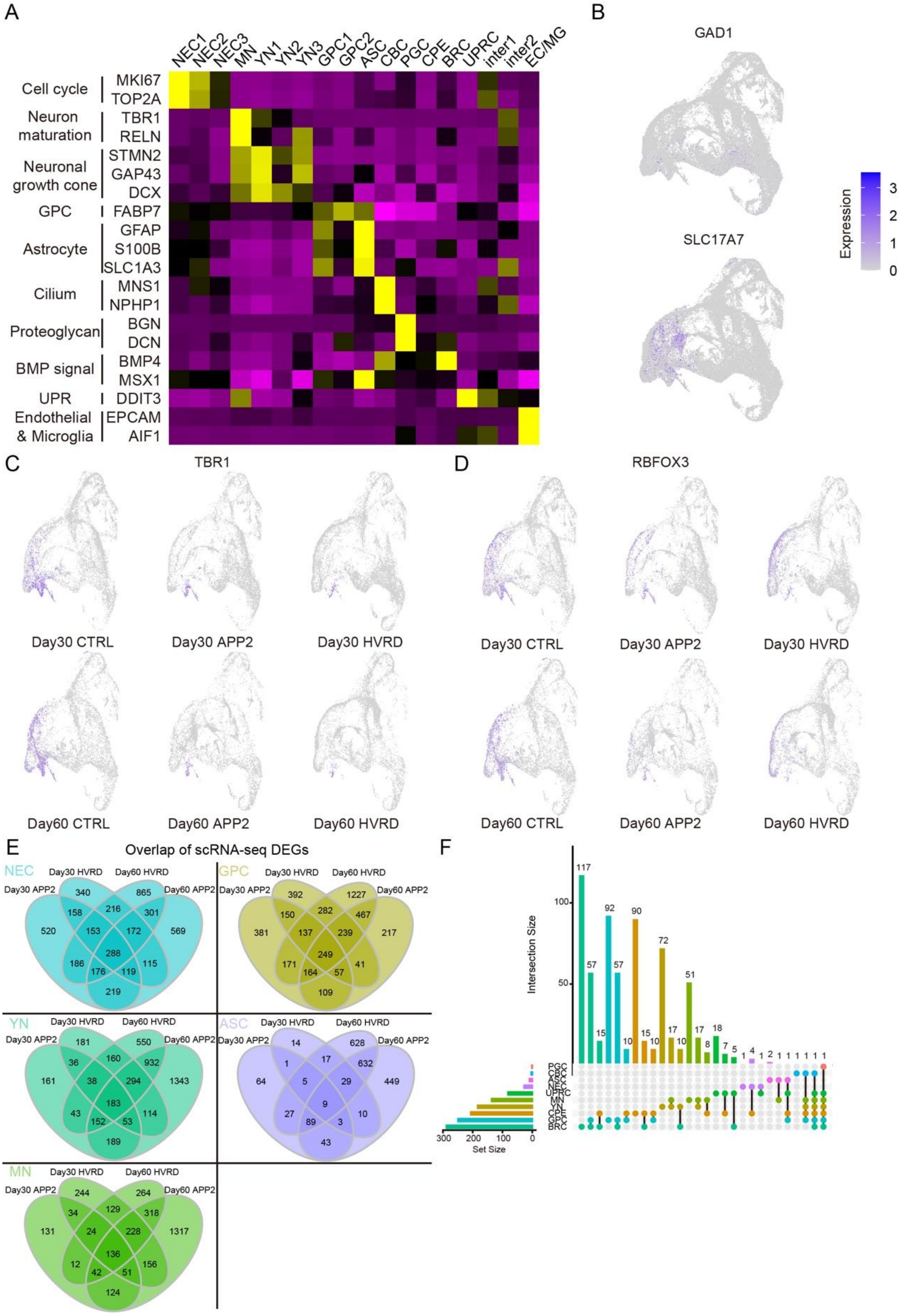

Figure S2. scRNA-seq analysis of cerebral organoids, related to Figure 1. (A)

Single-cell gene expression heat map of cell-type marker genes. (B) Expression patterns of markers for inhibitory neuron (*GAD1*) and excitatory neuron (*SLC17A7*). (C) Expression patterns of marker for sixth layer neuronal marker *TBR1* in indicated cerebral organoids. (D) Expression patterns of mature neuron marker NeuN (*RBFOX3*) in indicated organoids. (E) Venn diagrams for differential expressed genes detected in 5 major cell types (NEC: neuroepithelial cell; GPC: glia progenitor cell; YN: young neuron; ASC: astrocyte; MN: mature neuron) showing the overlaps between four fAD cerebral organoids (p.adj < 0.05). (F) Upset plot showing the overlaps between the set of differentially expressed genes identified in indicated cell types (p.adj < 0.05). CPE: choroid plexus epithelial; UPRC: unfolded-protein-response-related cell; BRC: BMP-related cell; CBC: Cilia-bearing cell; PGC: proteoglycan-expressing cell.

Figure S3

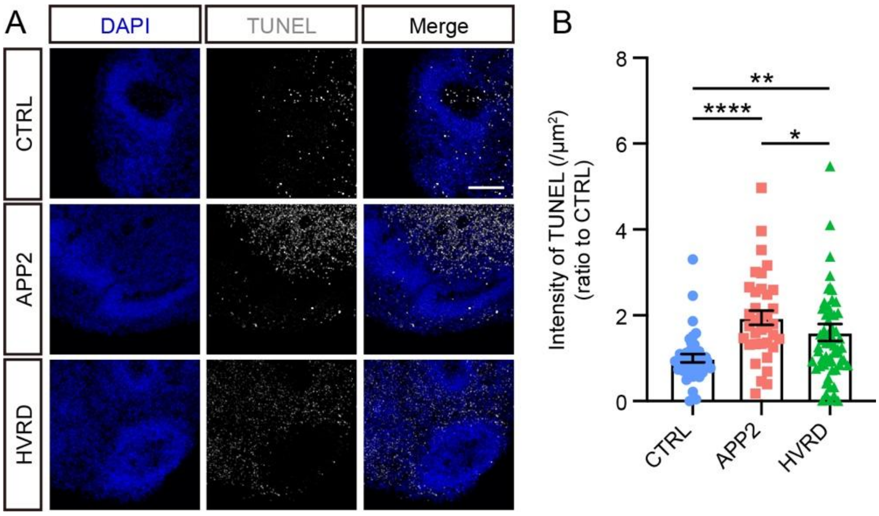

Figure S3. Cell death increases in fAD cerebral organoids, related to Figure 2. (A) Immunofluorescence for apoptosis signal (TUNEL) in D60 cerebaral organoids. Scale bar, 100 μm. (B) Quantification of the intensity of TUNEL signal in D60 cerebral organoids. Data are presented as mean ± SEM of at least 12 organoids (3 fields per organoid) per group from at least 2 independent experiments, with the value of control group normalized as 1.0. Mann-Whitney test. *P < 0.05, **P < 0.01, ****P < 0.0001.

Figure S4

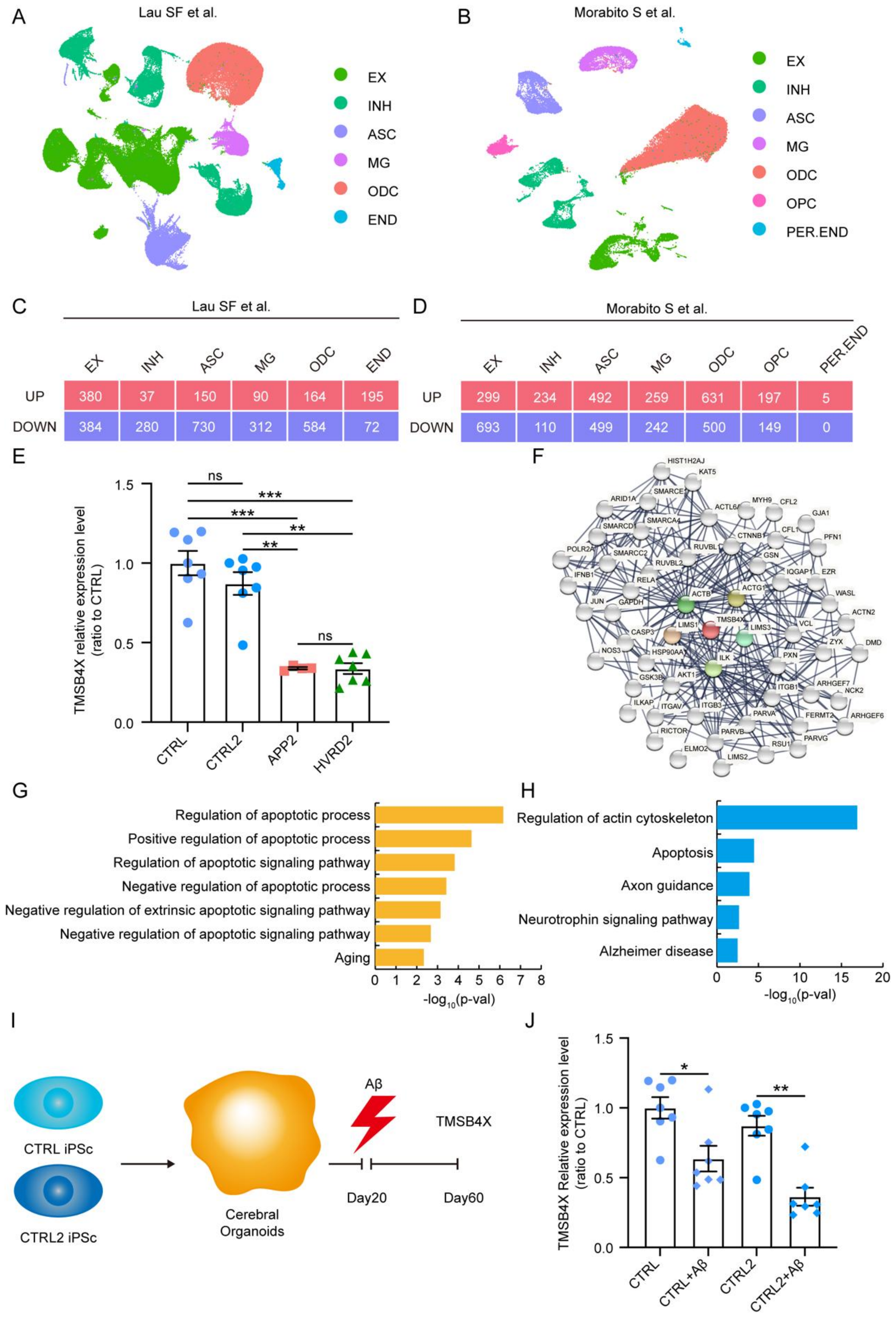

Figure S4. TMSB4X is related to aging and Alzheimer's disease pathways,

related to Figure 4. (A, B) UMAP visualization for major cell types identified by single-nuclei RNA-seq (snRNA-seq) analysis of AD brain samples. EX: excitatory neuron; INH: inhibitory neuron; ASC: astrocyte; MG: microglia; ODC: oligodendrocyte; OPC: oligodendrocyte progenitor; END: endothelial; PER.END: pericytes endothelial. (C, D) The number of differentially expressed genes identified in major cell types of AD brain samples. (p.adj <0.05). (E) The expression level of *TMSB4X* in D60 cerebral organoids. Data are presented as mean ± SEM of 4-7 organoids per group. The value of the control group was normalized as 1.0. Student's t-test. $^{**}P < 0.01$, $^{***}P < 0.001$. (F) Protein-protein interaction network for *TMSB4X* analyzed using the data from STRING database. (G) The top 7 GO terms enriched by *TMSB4X* interacting genes. (H) The top 5 KEGG pathways enriched by *TMSB4X* interacting genes. (I) Schematic representation of Aβ treatment of cerebral organoids and timeline of the analysis. (J) The expression level of *TMSB4X* in Aβ-treated cerebral organoids at D60. Data are presented as mean ± SEM of 4-7 organoids per group. The value of the control group was normalized as 1.0. Student's t-test. $^{*}P < 0.05$, $^{**}P < 0.01$.

Figure S5

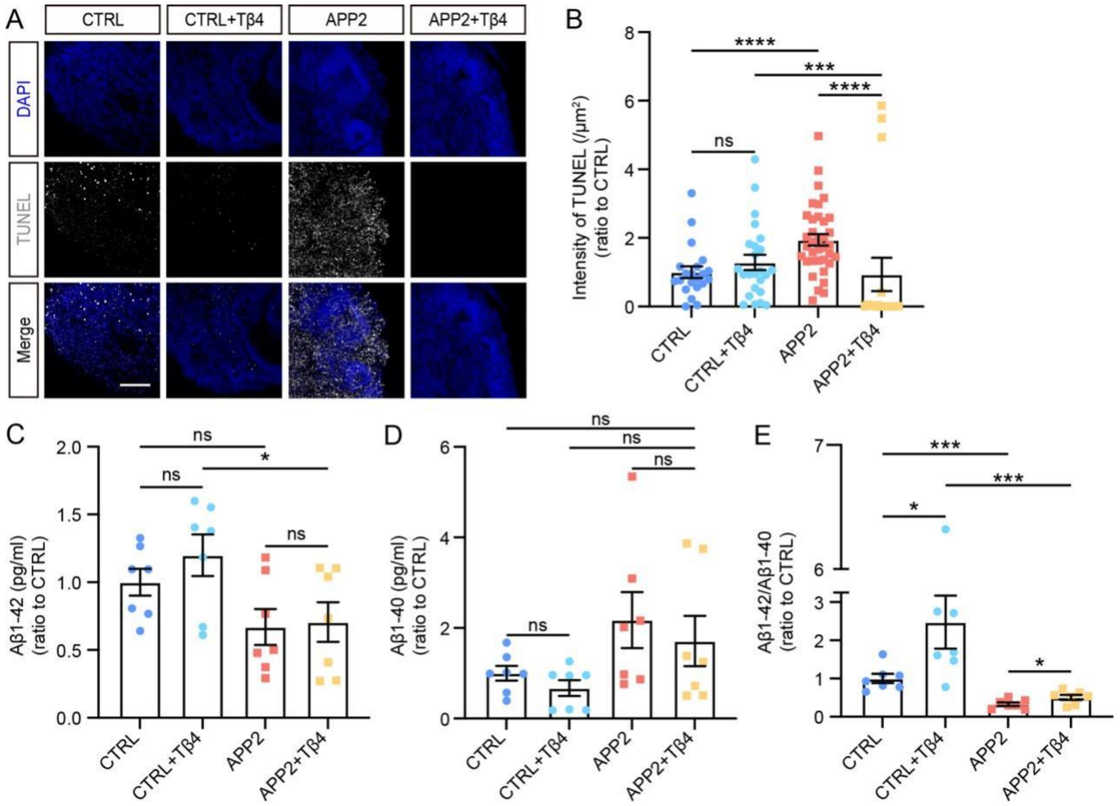

Figure S5. Thymosin β4 increases the ratios of Aβ1-42/Aβ1-40 and decreases cell death in fAD cerebral organoids, related to Figure 5. (A) Immunofluorescence for apoptosis signal (TUNEL) in D60 cerebral organoids. Scale bar, 100 μm. (B) Quantification of the intensity of TUNEL signal in D60 cerebral organoids. Data are presented as mean ± SEM of at least 6 organoids (3 fields per organoid) per group, with the value of control group normalized as 1.0. Mann-Whitney test. ***P < 0.001, ****P < 0.0001. (C) ELISA results of Aβ1-42 in the medium of D60 cerebral organoids. (D) ELISA results of Aβ1-40 in the medium of D60 cerebral organoids. (E) Ratios of Aβ1-42/Aβ1-40 from ELISA. Data are presented as mean ± SEM of 7 organoids per group. The value of the control group was normalized as 1.0. Mann-Whitney test. *P < 0.05, ***P < 0.001.

Figure S6

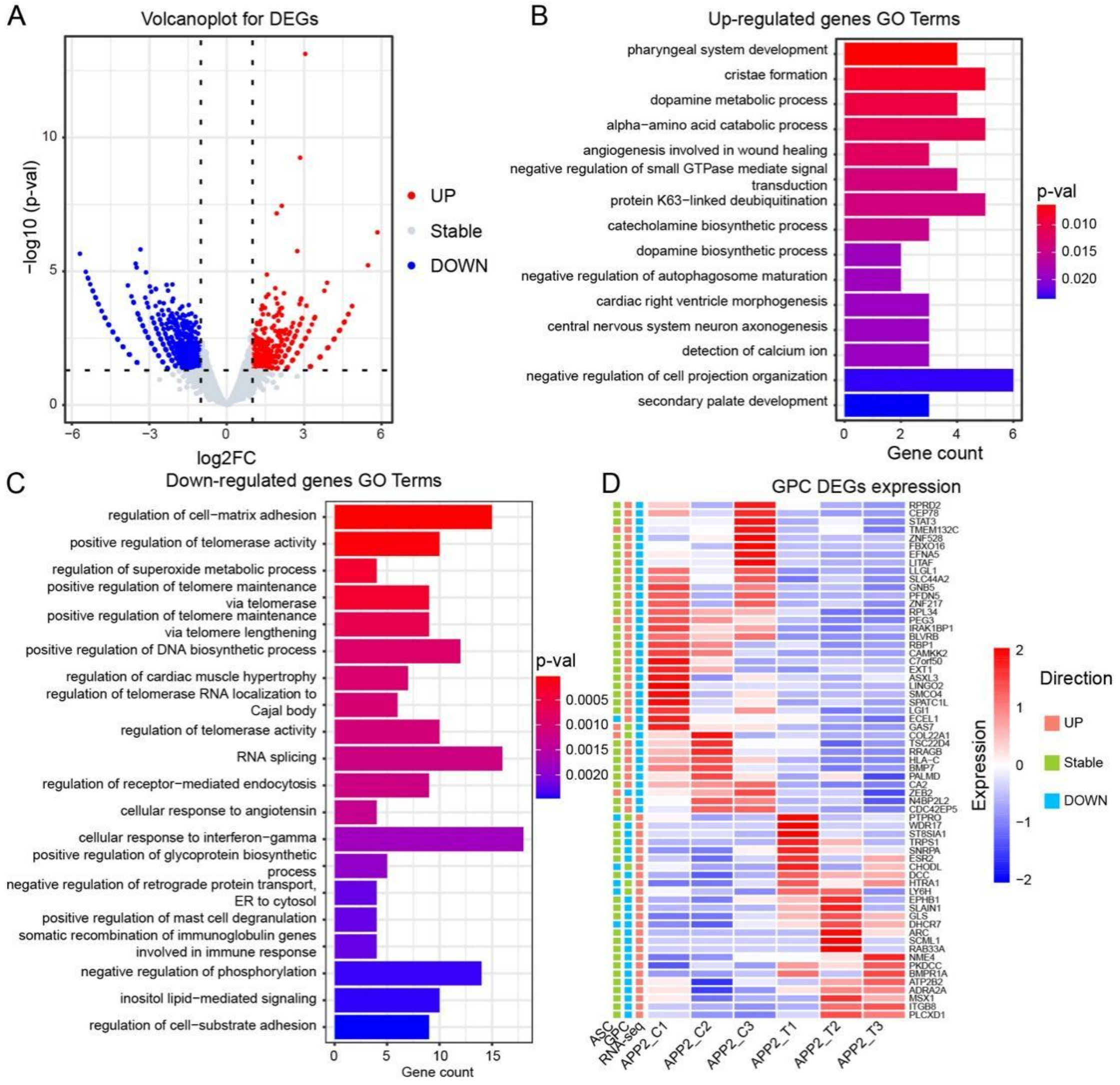

Figure S6. Bulk RNA-seq for thymosin β4-treated fAD cerebral organoids, related to Figure 5. (A) Volcano plot showing the number of differential expressed genes identified in thymosin β4 treated APP2 cerebral organoids. (B) The top 15 GO terms enriched by the up-regulated genes in thymosin β4 treated APP2 cerebral organoids. (C) The top 20 GO terms enriched by the down-regulated genes in thymosin β4 treated APP2 cerebral organoids. (D) Heatmap showing the differential expressed genes identified in thymosin β4 treated D30 APP2 cerebral organoids with different alternations in astrocyte and glia progenitor.

Figure S7

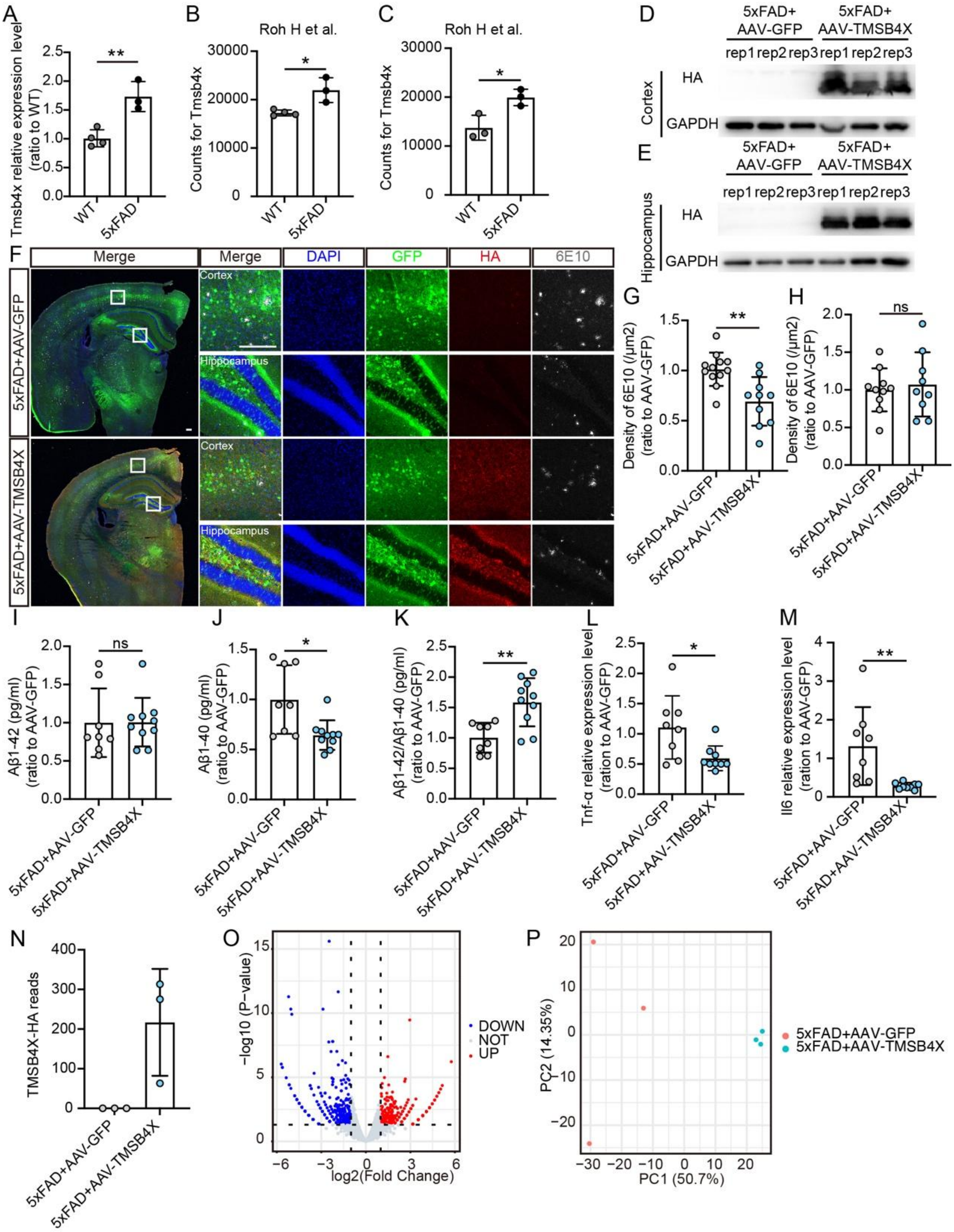

Figure S7. Administration of AAV-TMSB4X reduces amyloid plaques and neuroinflammation in 5xFAD mice, related to Figure 6 and Figure 7. (A) The expression level of *Tmsb4x* in hippocampus of WT and 5xFAD mice. Data are presented as mean ± SEM of at least 3 mice in each group with the value of WT mice

normalized as 1.0. Student's t-test. **P < 0.01. (B, C) The expression of *Tmsb4x* in bulk RNA-seq from 5xFAD mice at 6 months (B) or 11 months (C). Data are presented as mean ± SEM of at least 3 mice in each group with the value of WT mice normalized as 1.0. t test. *P < 0.05. (D, E) The expression of TMSB4X-HA in the cortex (D) or hippocampus (E) of AAV injected 5xFAD mice. Three mice were analyzed in each group (rep 1-3). (F) Immunofluorescence for GFP, HA and amyloid plaques (6E10) in AAV-GFP and AAV-TMSB4X injected 5xFAD mice. Scale bar, 100 μm. (G, H) Quantification of the density of amyloid plaques (6E10) in the cortex (G) or hippocampus (H) of AAV-GFP and AAV-TMSB4X injected 5xFAD mice. Data are presented as mean ± SEM of at least 9 mice in each group with the value of AAV-GFP group normalized as 1.0. Mann-Whitney test. **P < 0.01. (I) ELISA results of Aβ1-42 in in AAV-GFP and AAV-TMSB4X injected 5xFAD mice. (J) ELISA results of Aβ1-40 in AAV-GFP and AAV-TMSB4X injected 5xFAD mice. (K) Ratios of Aβ1-42/Aβ1-40 from ELISA. Data are presented as mean ± SEM of 7 mice per group. The value of AAV-GFP group was normalized as 1.0. Mann-Whitney test. *P < 0.05, **P < 0.01. (L) The expression level of *Tnf-α* in hippocampus of AAV-GFP and AAV-TMSB4X injected 5xFAD mice. Data are presented as mean ± SEM of at least 8 mice in each group with the value of AAV-GFP group normalized as 1.0. Student's t test. *P < 0.05. (M) The expression level of *Il6* in hippocampus of AAV-GFP and AAV-TMSB4X injected 5xFAD mice. Data are presented as mean ± SEM of at least 8 mice in each group with the value of AAV-GFP group normalized as 1.0. Student's t test. **P < 0.01. (N) The number of TMSB4X-HA reads in AAV-GFP and AAV-TMSB4X injected 5xFAD mice. (O) Volcano plot showing the number of differential expressed genes identified in AAV-TMSB4X injected 5xFAD mice compared to AAV-GFP injected 5xFAD mice. (P) PCA plot for AAV-GFP and AAV-TMSB4X injected 5xFAD mice.

Supplemental Table

Table S1. Information about the iPSCs used in this study, related to Figure 1 and Figure S4.

Cell Line	Cell Type	Disease	Gender	Age	Mutation	APOE genotype	Label
UCSD0931i-1-11	Human iPSC	None reported	Female	34	normal	APOE3/APOE3	CTRL
UCSD224i-NDC1-2	Human iPSC	None reported	Male	86	normal	APOE3/APOE3	CTRL2
HVRDi002-A-1	Human iPSC	Familial Alzheimer's disease	Female	33	APPV717I/-mutation	APOE3/APOE3	HVRD
HVRDi001-A-1	Human iPSC	Familial Alzheimer's disease	Male	57	APPV717I/-mutation	APOE3/APOE3	HVRD2
UCSD239i-App2-1	Human iPSC	Familial Alzheimer's disease	Female	60	APP (duplication)	APOE3/APOE3	APP2

Supplemental Methods

Animals

The mice were group-housed (3 to 5 mice per cage) in the institutional animal care facility, with a stable environmental temperature of 21 °C ± 1 °C, a humidity level controlled at 50% ± 10%, and a regular 12-hr light-dark cycle implemented. The 5xFAD mice (B6SJL-Tg [APP-SwFlLon, PS1*M146L*L286V] 6799Vas/J, JAX stock number: 034840) were crossed with C57 mice (C57BL/6, JAX stock number: 000664) to generate offsprings, with the day of birth being defined as postnatal day 0 (P0). Both female and male mice were incorporated into the experiments and involved in the result-recording processes.

iPSC culture

The iPSCs used in this study were purchased from WiCell company and were tested previously (Israel et al., 2012). The detailed information was showed in Supplementary Table S1. Cells were cultured on hESC-Matrigel (BD-Biocoat, #354277) coated dishes in mTeSR Plus (STEMCELL, #85850) medium with the addition of bFGF (STEMCELL, #78003) (4 μg/ml). The culture medium was half-replaced every day and then cells were passaged every 5 days by passage reagent ReLeSR (STEMCELL, #05872). The cells were tested for mycoplasma and sterility before each passage.

Cerebral organoid culture

Cerebral organoids were cultured following instructions of the commercial STEMdiffTM Cerebral Organoid Kit (STEMCELL #08570). On day 0, iPSCs colonies were dissociated into single-cell suspension using Accutase and around 9000 cells were seeded into Lipidure (#CM5206)-coated V-bottom well of ultra-low-attachment 96-well plate by 100 μl EB Formation Medium containing 1 μM Y27632 (STEMCELL, #72304). On day 2 and day 4, 100 μl EB Formation Medium without Y27632 was added into each well, respectively. On day 5, 200 μl Induction

Medium after aspirating the EB Formation Medium was added. On day 7, each EB (embryonic body) was embedded into 15 μl of Matrigel (BD-Biocoat, #354230) and cultured in 10-cm dishes with 5 ml Expansion Medium for 3 days for organoid formation. On day 10, cerebral organoids were moved into T25 flask with 15 ml Maturation Medium on a shaker. The medium was replaced every 3-4 days.

Single-cell RNA-seq

Organoids were dissociated using the methods as described in previous study (Thomsen et al., 2016). Briefly, 8-10 organoids were pooled and washed in DPBS (Life/Invitrogen, #14190144), then cut into small pieces, which were then incubated with 2 ml trypsin solution (Ca_2^+/Mg_2^+-free HBSS with 10 mM HEPES, 2 mM $MgCl_2$, 10 μg/ml DNase I, 0.25 mg/ml trypsin) for 30 min at 37°C, quenched with 4 ml Quenching Buffer. Following is the formulation of Quenching Buffer: 440ml Leibovitz L-15 medium (Thermo, #11415064), 50 ml ddH_2O, 5 ml 1M HEPES (PH 7.3-7.4), 10 μg/ml DNase I, 100 nM TTX (TOCRIS, #1069), 20 μM DNQX (TOCRIS, #0189), 50 μM DL-AP5 (TOCRIS, #3693), 5 ml 100x Anti-Anti, 2 mg/ml BSA, 100 μg/ml trypsin inhibitor (Sigma-Aldrich, #T6522). The dissociated cells were precipitated by centrifugation (220×g for 4 min at 4°C), resuspended with 2 ml Staining Medium (440ml Leibovitz L-15 medium with 50 ml ddH_2O, 5ml 1M HEPES (PH 7.3-7.4), 1g BSA, 100 nM TTX, 20 μM DNQX, and 50 μM DL-AP5, 5 ml 100 x Anti-Anti, 20 ml 77.7 mM EDTA (PH 8.0)), filtered through a 40-micron cell filter, and centrifuged again (220×g for 4 min at 4°C). Then the cell pellets were resuspended in 5 ml DPBS with 1% BSA. Dissociated cells were resuspended at a concentration of 500 cells/μl. cDNA libraries were generated by Novogene company using Single Cell Reagent Kits (10x GENOMICS), with individual cell and transcript barcoded.

Single-cell RNA sequence processing and integration

Raw data of gene expression obtained with 10x Genomics was processed using CellRanger software (v6.1.2), with human genome data version GRCh38 (v107) as

reference, generating datasets including matrix, features and barcodes information. Seurat (v4.2.0 R package) was used for quality control and integrating the single-cell transcriptome data (Macosko et al., 2015). The function called "Read10X" was used to load 10x Genomics data to R environment. For quality control, the cells with less than 300 or more than 7500 detected genes and with mitochondrial gene proportion higher than 20% were excluded. Filtered data were normalized with LogNormalize using "NormalizeData" function with default parameters and combined by "IntegratedData" function using top 20 anchors based on 2000 genes with the highest residual variance. Finally, we obtained 17644 features and 54130 cells.

Cell type dimensionality reduction and cluster analysis

For the combined data, we scaled the data by "ScaleData" function with default parameters and performed principal component analysis (PCA) based on 2000 genes with the highest residual variance. The clustering and dimensional reduction for each cell were conducted with the Seurat functions with default parameters using the top 20 principal components and 0.3 resolution. The marker genes for each cluster were identified with >0.25 average log2 fold change and < 0.1 adjusted p-value. Over-represented GO terms for these marker genes were identified by clusterProfiler (v4.4.4 R package) and org.Hs.eg.db (v3.15.0 R package) (Kuleshov et al., 2016; Wu et al., 2021).

Clusters were classified following the rules as described (Tanaka et al., 2020). Firstly, the clusters were separated by general neural genes (*SIMN2* and *DCX*) and early neurogenesis markers (*VIM, HES1, SOX2*) into neuronal and non-neuronal group. The neuronal groups were classified into mature neuron (MN) and young neuron (YN) by *YBR1*. For the non-neuronal groups, proteoglycan-expressing cell (PGC) was identified by *BGN* and *DCN*, and Cilia-bearing cell (CBC), BMP-related cell (BRC) and unfolded-protein-response-related cell (UPRC) were identified by GO terms "cilium assembly (GO:0044458)", "response to BMP (GO:0030509)" and "endoplasmic reticulum unfolded protein response (GO:0030968)". Neuroepithelial

cell (NEC) was identified by cell cycle-related genes such as *MKI67*. Glial progenitor cells were classified by “glial cell differentiation (GO:0010001)”. Astrocyte (ASC) were identified by *GFAP* and *SLC1A3*. Choroid plexus epithelial (CPE) was identified by TTR. Endothelia cell/microglia (EC/MG) were identified by EPCAM and AIF1. The remaining cells were distinguished as intermediate (inter).

Single-cell data comparison of control and fAD cerebral organoids

To analyze the cell-type-specific DEGs between control and fAD cerebral organoids, we calculated the significance of difference by wilcox rank sum testing. The genes with p-value < 0.05 and log2 fold change > 0.1 were identified as cell-type-specific DEGs. To identify biological processes associated with these DEGs, we used the enrichR (v3.2 R package) to query enriched GO terms and KEGG pathways for these cell-type-specific DEGs in the GO Biological Processes 2021, GO Cellular Component 2021, GO Molecular Function 2021 and KEGG 2019 Human.

Trajectory analysis

The pseudotime trajectory analysis followed the protocol as described in monocle (v2.24.1 R package) website (Cao et al., 2019; Qiu et al., 2017; Trapnell et al., 2014). Briefly, the data were extracted from Seurat analysis processing and monocle object was built by feature data, expression data and meta data. After calculating size factors and dispersions, 2000 genes with the highest residual variance were used for downstream analysis. Finally, the results were obtained by running “reduceDiemsion” and “orderCells”

Gene module score analysis

Gene module scores for particular gene sets were computed by Seurat “AddModuleScore” function. The particular gene lists used in our study include: GFAP ^{high} signature (*GFAP, ID3, AQP4, MYOC, ID1, FABP7*); GFAP^{low} signature (*LUZP2, SLC7A10, MFGE8*); Disease associated astrocyte (DAA) signature (*GFAP, CSTB, VIM, OSMR, GSN*); Up in YN signature (upregulated DEGs in young neurons

of four fAD organoids); Down in YN signature (downregulated DEGs in young neurons of four fAD organoids); Up in MN signature (upregulated DEGs in mature neurons of four fAD organoids); Down in MN signature (downregulated DEGs in mature neurons of four fAD organoids); Up in ASC signature (upregulated DEGs in astrocytes of four fAD organoids); Down in ASC signature (downregulated DEGs in astrocytes of four fAD organoids); Neuron state 1 signature (marker genes of state 1 in trajectory analysis of young neuron and mature neuron in cerebral organoids); Neuron state 7 signature (marker genes of state 7 in trajectory analysis of young neuron and mature neuron in cerebral organoids); Astrocyte state 5 signature (marker genes of state 1 in trajectory analysis of astrocyte and glia progenitor cell in cerebral organoids); Astrocyte state 3 signature (marker genes of state 3 in trajectory analysis of astrocyte and glia progenitor cell in cerebral organoids).

Immunofluorescence

The cerebral organoids were fixed in 4% paraformaldehyde (PFA) for 30-60 min after 3 washes with PBS, dehydrated with 30% sucrose in PBS overnight at 4 °C, embedded into optical cutting temperature (OCT) (Sakura, #4583) and frozen in -20 °C and cryosectioned into 35 μm-thick slides, which were then permeabilized in 0.5% Triton (Sigma-Aldrich, #T8787) and blocked with 5% BSA (Sigma-Aldrich, #V900933) in 0.1% Triton for 1 hr. Then, slides were incubated with primary antibodies in 5% BSA for two nights at 4 °C, washed with PBS three times, and incubated with Fluor-conjugated secondary antibodies overnight at 4 °C. Stained slides sections were mounted with mounting medium after washing three times with PBS. For mouse brain sections, the following procedures were carried out. Firstly, the animals were perfused with PBS. Then, the brains were fixed in 4% PFA at 4 °C for two nights, followed by dehydration in 30% sucrose dissolved in PBS at 4 °C for another two nights. The dehydrated brains were embedded in OCT compound and then frozen at -20 °C, and then cryosectioned with a thickness of 50 μm. After permeabilization in 0.5% Triton for 1 hr and blocking with 5% BSA in 0.1% Triton for 1 hr, brain sections were incubated with primary antibodies in 5% BSA over-night

at 4 °C and incubated with Fluor-conjugated secondary antibodies for 2 hr after washing three times with PBS. After three additional washes with PBS, the stained sections were mounted using mounting medium. Fluorescence signals were taken using laser scanning confocal microscopy and analyzed by Image J software. The primary antibodies include: β-amyloid (Cell Signaling, #8243T, 1:1000); PAX6 (R&D, #AF8150, 1:500); SOX2 (Santa Cruz, #sc-17320, 1:400); DCX (Santa Cruz, #sc-8006, 1:1000); TBR2 (R&D, #AF6166, 1:1000); TBR1 (Abcam, #ab31940, 1:1000); CTIP2 (Abcam, #ab18465, 1:1000); MAP2 (Sigma-Aldrich, #AB5622, 1:1000); NEUN (Millipore, #MAB377, 1:1000); c-CASP3 (Cell Signaling, #9661L, 1:1000); 6E10 (BioLegend, #803004, 1:1000); IBA1 (Oasis, #OB-PGP049-02, 1:1000); HA (Cell Signaling, #3724S, 1:1000); GFP (Abcam, #ab31970, 1:1000); GFAP (Oasis, #OB-PGP055, 1:1000). The secondary antibodies were Alexa Fluor 488, 555, 594, or 647-conjugated donkey anti-mouse, -rabbit, -rat, -pig or -chicken IgG (Invitrogen, all used at 1:1000 dilution).

TUNEL staining

Frozen cerebral organoids sections were washed three times with PBS for 5 min each, and then incubated with DAPI (Beyotime, #C1002, 1:1000) in 5% BSA over-night at 4 °C. Next, the sections were washed three times with PBS for 5 min each. Subsequently, the sections were stained for TUNEL using TUNEL apoptosis detection kit (Alexa Fluor 640) (Yeasen, #40308ES60).

Thioflavin S staining

Frozen mice brain sections were washed three times with PBS for 5 min each, and then incubated with 1 mg/ml Thioflavin S (Sigma-Aldrich, #T1892) for 7 min. The sections were then sequentially washed with 90%, 80% and 70% alcohol for 7 min each. Subsequently, they were washed three more times with PBS for 5 min each to remove any remaining alcohol. Finally, the sections were mounted and imaged.

ELISA for Aβ1-42 and Aβ1-40

The total amount of protein in cerebral organoids' culture medium and mouse brain tissue was measured by BCA protein quantification kit (YEASEN, #B2419081), and the protein amount was normalized based on the BCA detected results. The normalized proteins were then used to detect the Aβ1-42 and Aβ1-40 levels by the ELISA kits. Detection kits used were as follows: human Aβ1-40 (share-bio, #SB-FY6715-48T), human Aβ1-42 (share-bio, #SB-FY6716-48T), mouse Aβ1-40 (share-bio, #SB-FY6378-48T), mouse Aβ1-42(share-bio, #SB-FY6379-48T).

Quantitative PCR (qPCR)

Quantitative PCR was performed by using the Agilent Mx3000P qPCR system with the 2xSYBR Green qPCR Master Mix (Bimake). Relative mRNA expression was determined by the delta cycle time with human 18S as the internal control in data normalization for cerebral organoids and mouse β-actin as the internal control in data normalization for mouse tissue. Primer sequences were as follows:

18S: forward, 5'- ATCACCATTATGCAGAATCCACG-3', reverse, 5'-GACCTGGCTGTATTTTCCATCC-3';

TMSB4X: forward, 5'-CGAATCGTAATGAGGCGTGC-3', reverse, 5'-TCCCTGCCAGCCAGATAGAT-3';

β-actin: forward, 5'-GAGACCTTCAACACCCCAGC-3', reverse, 5'-ATGTCACGCACGATTTCCC-3';

Tmsb4x: forward, 5'-CGTCCTTAAAGCCAAGTCCAAG-3', reverse, 5'-TACAGTGCATATTGGCGGCG-3';

Tnf-α: forward, 5'-ACCCTCACACTCACAAACCA-3', reverse, 5'-ATAGCAAATCGGCTGACGGT-3';

Il6: forward, 5'-GCCTTCTTGGGACTGATGCT-3', reverse, 5'-GTGACTCCAGCTTATCTCTTGGT-3'.

Electrophysiological recording

Transverse cortical slices were prepared from 5.5–7-month-old WT, 5xFAD, AAV-GFP or AAV-TMSB4X injected 5xFAD mice. The mice were anesthetized with

avertin (625 mg/kg, i.p.) and then perfused with cold, oxygenated NMDG ACSF solution (93 mM NMDG, 93 mM HCl, 2.5 mM KCl, 1.25 mM NaH_2PO_4, 10 mM $MgSO_4·7H2O$, 30 mM $NaHCO_3$, 25 mM glucose, 20 mM HEPES, 5 mM sodium ascorbate, 3 mM sodium pyruvate, and 2 mM thiourea). After perfusion, the brain was rapidly dissected and transferred to a cold NMDG ACSF solution, and then sectioned coronally using a vibratome (VT1200 S, Leica) in the same buffer at a thickness of 300 μm. The cortical slices were incubated in oxygenated NMDG ACSF at 32 °C for 10-15 min and subsequently transferred to a normal oxygenated ACSF solution (126 mM NaCl, 2.5 mM KCl, 1.25 mM NaH_2PO_4, 2 mM $MgSO_4·7H_2O$, 10 mM glucose, 26 mM $NaHCO_3$, 2 mM $CaCl_2$) at room temperature for one hour. All chemicals used in the preparation of the slices were obtained from Sigma-Aldrich (St. Louis, MO, USA).

The brain slices were carefully transferred to a recording chamber that was completely submerged and continuously perfused with ACSF solution at a flow rate of 3 mL/min, and maintained at 28 °C. The neurons for recoding were visualized by differential interference contrast optics (DIC; Olympus BX61WI). The recording pipettes with 3 to 4 MΩ resistance were fabricated using a micropipette puller (P2000, Sutter Instrument; USA). For whole-cell recordings, the pipettes were filled with an ACSF solution composed of 133 mM potassium gluconate, 18 mM NaCl, 0.6 mM EGTA, 10 mM HEPES, 2 mM Mg·ATP, and 0.3 mM $NA_3·GTP$ (pH 7.2, 280 mOsm). Once a whole-cell configuration was successfully established, the neurons were voltage-clamped at -70 mV. To evoke action potentials (AP), a current-step protocol was repeatedly applied, with the current ranging from -20 to +400 pA in increments of 20 pA. Detection and analysis of AP were performed by Clampfit Program.

References

Cao, J., Spielmann, M., Qiu, X., Huang, X., Ibrahim, D.M., Hill, A.J., Zhang, F., Mundlos, S., Christiansen, L., Steemers, F.J., et al. (2019). The single-cell

transcriptional landscape of mammalian organogenesis. Nature *566*, 496-502. 10.1038/s41586-019-0969-x.

Israel, M.A., Yuan, S.H., Bardy, C., Reyna, S.M., Mu, Y., Herrera, C., Hefferan, M.P., Van Gorp, S., Nazor, K.L., Boscolo, F.S., et al. (2012). Probing sporadic and familial Alzheimer's disease using induced pluripotent stem cells. Nature *482*, 216-220. 10.1038/nature10821.

Kuleshov, M.V., Jones, M.R., Rouillard, A.D., Fernandez, N.F., Duan, Q., Wang, Z., Koplev, S., Jenkins, S.L., Jagodnik, K.M., Lachmann, A., et al. (2016). Enrichr: a comprehensive gene set enrichment analysis web server 2016 update. Nucleic Acids Res *44*, W90-97. 10.1093/nar/gkw377.

Macosko, E.Z., Basu, A., Satija, R., Nemesh, J., Shekhar, K., Goldman, M., Tirosh, I., Bialas, A.R., Kamitaki, N., Martersteck, E.M., et al. (2015). Highly Parallel Genome-wide Expression Profiling of Individual Cells Using Nanoliter Droplets. Cell *161*, 1202-1214. 10.1016/j.cell.2015.05.002.

Qiu, X., Mao, Q., Tang, Y., Wang, L., Chawla, R., Pliner, H.A., and Trapnell, C. (2017). Reversed graph embedding resolves complex single-cell trajectories. Nat Methods *14*, 979-982. 10.1038/nmeth.4402.

Tanaka, Y., Cakir, B., Xiang, Y., Sullivan, G.J., and Park, I.H. (2020). Synthetic Analyses of Single-Cell Transcriptomes from Multiple Brain Organoids and Fetal Brain. Cell Rep *30*, 1682-1689 e1683. 10.1016/j.celrep.2020.01.038.

Thomsen, E.R., Mich, J.K., Yao, Z., Hodge, R.D., Doyle, A.M., Jang, S., Shehata, S.I., Nelson, A.M., Shapovalova, N.V., Levi, B.P., and Ramanathan, S. (2016). Fixed single-cell transcriptomic characterization of human radial glial diversity. Nat Methods *13*, 87-93. 10.1038/nmeth.3629.

Trapnell, C., Cacchiarelli, D., Grimsby, J., Pokharel, P., Li, S., Morse, M., Lennon, N.J., Livak, K.J., Mikkelsen, T.S., and Rinn, J.L. (2014). The dynamics and regulators of cell fate decisions are revealed by pseudotemporal ordering of single cells. Nat Biotechnol *32*, 381-386. 10.1038/nbt.2859.

Wu, T., Hu, E., Xu, S., Chen, M., Guo, P., Dai, Z., Feng, T., Zhou, L., Tang, W., Zhan, L., et al. (2021). clusterProfiler 4.0: A universal enrichment tool for interpreting omics

data. Innovation (Camb) *2*, 100141. 10.1016/j.xinn.2021.100141.
